# Glioma-derived IL-33 orchestrates an inflammatory brain tumor microenvironment that accelerates glioma progression

**DOI:** 10.1038/s41467-020-18569-4

**Published:** 2020-10-05

**Authors:** Astrid De Boeck, Bo Young Ahn, Charlotte D’Mello, Xueqing Lun, Shyam V. Menon, Mana M. Alshehri, Frank Szulzewsky, Yaoqing Shen, Lubaba Khan, Ngoc Ha Dang, Elliott Reichardt, Kimberly-Ann Goring, Jennifer King, Cameron J. Grisdale, Natalie Grinshtein, Dolores Hambardzumyan, Karlyne M. Reilly, Michael D. Blough, J. Gregory Cairncross, V. Wee Yong, Marco A. Marra, Steven J. M. Jones, David R. Kaplan, Kathy D. McCoy, Eric C. Holland, Pinaki Bose, Jennifer A. Chan, Stephen M. Robbins, Donna L. Senger

**Affiliations:** 1 Clark Smith Brain Tumour Centre, Arnie Charbonneau Cancer Institute, Cumming School of Medicine, University of Calgary, Calgary, AB Canada; 2 Divison of Human Biology, Fred Hutchinson Cancer Research Center, Seattle, WA United States; 3 Canada’s Michael Smith Genome Sciences Centre, British Columbia Cancer Agency, Vancouver, BC Canada; 4 Department of Molecular Genetics, University of Toronto and Program in Neurosciences and Mental Health, Hospital for Sick Children, Toronto, ON Canada; 5 Department of Oncological Sciences, The Tisch Cancer Institute and the Department of Neurosurgery, Icahn School of Medicine at Mount Sinai, New York, New York United States; 6 Center for Cancer Research, National Cancer Institute, Bethesda, MD United States; 7 Department of Clinical Neurosciences, Cumming School of Medicine, University of Calgary, Calgary, AB Canada; 8 Department of Physiology and Pharmacology, Cumming School of Medicine, University of Calgary, Calgary, AB Canada; 9 Department of Pathology, Cumming School of Medicine, University of Calgary, Calgary, AB Canada; 10 Department of Oncology, Cumming School of Medicine, University of Calgary, Calgary, AB Canada; 11 Present Address: King Abdullah International Medical Research Center, King Saud Bin Abdulaziz University for Health Sciences, King Abdulaziz Medical City, Ministry of National Guard Health Affairs, Riyadh, Saudi Arabia

**Keywords:** Cancer microenvironment, CNS cancer

## Abstract

Despite a deeper molecular understanding, human glioblastoma remains one of the most treatment refractory and fatal cancers. It is known that the presence of macrophages and microglia impact glioblastoma tumorigenesis and prevent durable response. Herein we identify the dual function cytokine IL-33 as an orchestrator of the glioblastoma microenvironment that contributes to tumorigenesis. We find that IL-33 expression in a large subset of human glioma specimens and murine models correlates with increased tumor-associated macrophages/monocytes/microglia. In addition, nuclear and secreted functions of IL-33 regulate chemokines that collectively recruit and activate circulating and resident innate immune cells creating a pro-tumorigenic environment. Conversely, loss of nuclear IL-33 cripples recruitment, dramatically suppresses glioma growth, and increases survival. Our data supports the paradigm that recruitment and activation of immune cells, when instructed appropriately, offer a therapeutic strategy that switches the focus from the cancer cell alone to one that includes the normal host environment.

## Abstract (web summary)

Elevated levels of interleukin-33 have been associated with poor prognosis in patients with glioma. Here the authors show that glioma-derived IL-33 modulates a pro-tumorigenic immune microenvironment by activating resident and recruiting peripheral innate immune cells.

## Introduction

Glioblastoma (GBM) is a high-grade glioma that despite a deep molecular understanding of its genomic mutational landscape, median overall survival is limited to 14.6 months^1,2^. While there is progress in the treatment of GBM by combining maximal surgical resection with radiotherapy, and concurrent and adjuvant temozolomide chemotherapy, there are very few long-term survivors with 2 and 5-year survival rates remaining at 25% and 10%, respectively^1,2^. The complex cellular composition, diffuse invasiveness, and the presence of a population of highly invasive and chemo- and radio-resistant brain tumor-initiating cells (BTICs)^3–9^ represent major obstacles in the development of effective treatments. In addition, difficulties in drug delivery across the blood–brain barrier and cellular adaptation within the tumor environment further contribute to their refractory behavior.

It is now clear that the presence of diverse cell types within tumors with different activated states can dramatically impact therapeutic response^10^ and together with the complex interaction with the extracellular matrix, tumor vasculature, and the normal stromal compartment, including immune cells and fuel tumor progression^11–15^. Specifically, the brain’s unique composition of cell types, including neuronal and glial progenitors, neurons, astrocytes, oligodendrocytes, microglia/macrophages, and brain endothelial cells all have capacity to impact glioma growth and invasion^16^, with tumor-associated macrophages (TAMs) being linked to high tumor grade and poor prognosis in many cancers, including glioma^17–20^. While the relative importance of the resident microglia and the blood-borne monocytes and macrophages in glioma tumorigenesis is under debate, there is substantial literature on the interactions of these cells within glioma^21,22^, including their ability to suppress immune surveillance functions^23,24^. Depending on the context, macrophage are functionally defined as classically activated (M1) anti-tumorigenic^25–32^ or alternatively activated (M2) pro-tumorigenic^33–37^. While these states are described as binary based on in vitro data, the in vivo characterization remains more mixed and is more consistent with the M1 and M2 phenotypes present on the opposite ends of a dynamic spectrum^38^. Utilizing these traits, we previously tailored conditions where TAMs inhibited glioma growth by programming microglia, and tissue-infiltrated macrophage with amphotericin B to suppress tumor growth and improve the overall survival in patient-derived intracranial xenograft models^39^. Similarly, a study by Joyce and colleagues^40^ found that the inhibition of the CSF-1R pathway can alter macrophage polarization and convert pro-tumorigenic to an anti-tumorigenic phenotype that results in suppression of glioma growth.

To identify factors that are important in driving tumor progression, we utilized orthotopic xenograft models established from patient-derived BTICs that are highly tumorigenic^41^, have the capacity to self-renew, differentiate^12,42,43^, harbor the spectrum of molecular genetic alterations that occur in human GBM (e.g., mutations in p53, PTEN, IDH1, EGFR, and MSH6 (refs. ^13,44–46^)) and model the human disease in vivo^43,45,47^. We found that glioma cells secrete IL-33, a member of the IL-1 cytokine family that can exert its effects by binding and activating the cell surface receptor ST2 (ref. ^48^), and its downstream signaling pathways or by translocation to the nucleus to bind chromatin to regulate the gene transcription^49,50^. Consistent with the complex regulation and localization of this protein, IL-33 can induce numerous biological functions in multiple types of innate and adaptive immune cells, including acting as a potent inducer of type 2 immune response, inducing maturation of mast cells, mediating survival, expansion, and recruitment of eosinophils, and modulating polarization of macrophages for antimicrobial activity^48,51–54^. In the brain, IL-33 is expressed by astrocytes and oligodendrocytes^52,55^, and in models of CNS damage, where it promotes microglial and macrophage alternative activation, a process that limits glial scarring^56,57^. Astrocyte-derived IL-33 also promote synapse homeostasis by microglia during early CNS development^58^. In addition, there is emerging data to suggest a pro-tumorigenic role for IL-33 in various cancers^59^, including glioma, where IL-33 expression correlates with poor prognosis^60,61^ and increased tumor growth with observed macrophage infiltration in a rat glioma model^62^.

Herein, we reinforce a role for IL-33 as a major regulator of the brain tumor environment and provide mechanistic insight into how this environment promotes tumorigenesis. We find that ~50% of surgical samples that cover the molecular and clinical heterogeneity of GBM patients, and their corresponding BTIC cultures, express and secrete IL-33 in vivo, which in turn alters resident microglia, and the recruitment and activation of bone-marrow-derived macrophages (BMDMs), natural killer cells, and T cells. Moreover, we find that the expression of IL-33 by glioma cells is sufficient to drive tumor progression and reduce overall survival, a function that requires both its secreted and nuclear functions. IL-33 is a major orchestrator of the GBM cellular microenvironment and highlights the possibility of altering or co-targeting the host environment, as an additional therapeutic strategy to overcome resistance and improve outcomes for patients with GBM.

## Results

### Presence of IL-33 in inflammatory GBM

**Fig. 1:**
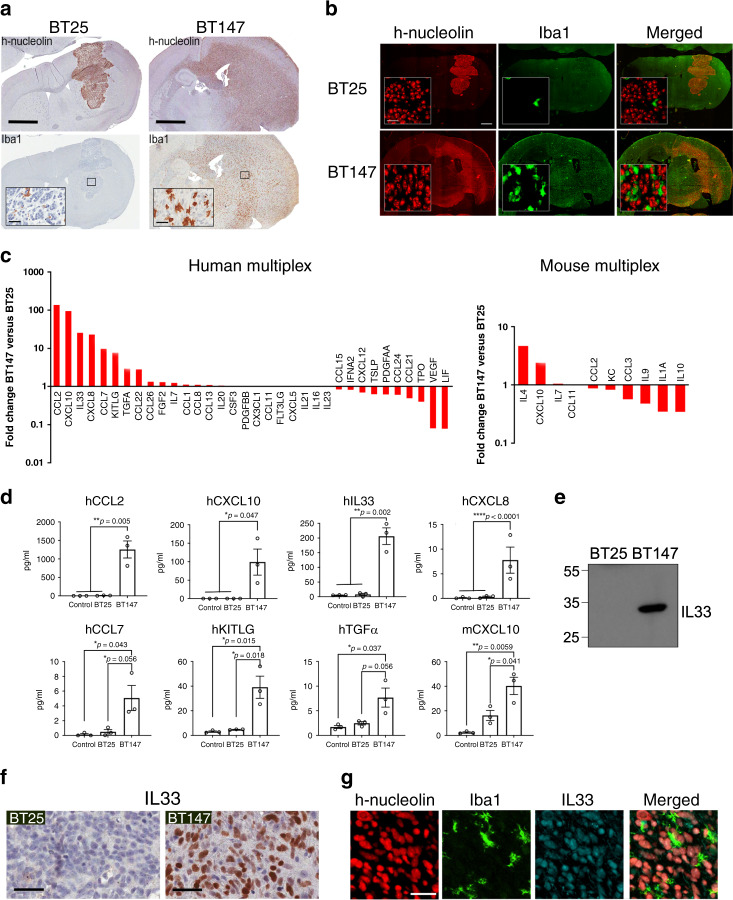
IL-33 is a component of the inflammatory secretome within glioblastoma. **a** Representative images of immunohistochemical staining of human-specific nucleolin (h-nucleolin) or the microglia/macrophage marker, Iba1 (brown), in intracerebral xenografts derived from BT25 and BT147 (*N* = 5). Scale bar, 2 mm; inset scale bar, 50 µm. **b** Representative images of immunofluorescent (IF) double staining of h-nucleolin (red) and Iba1 (green) in tumors derived from BT25 or BT147 (*N* = 3). Scale bar, 2 mm; inset scale bar, 30 µm. **c** Graph shows differential cytokine expression in the tumor interstitial fluid (TIF) from noninflammatory BT25 and inflammatory BT147 xenografts, measured using human 65-plex (left panel) and mouse 32-plex (right panel) Luminex arrays. Results are expressed as fold change in BT147 vs. BT25 xenografts determined from the mean of three independent experiments. **d** Graphs show cytokine levels of significantly upregulated human or mouse cytokines in the interstitial fluid of control (sham-injected), BT25, or BT147 intracranial xenografts. Data are the mean ± SEM from three independent experiments; *p ≤ 0.05, **p ≤ 0.01, ****p ≤ 0.0001 by one-way ANOVA with Tukey’s post hoc test. **e** Representative western blot of TIF from BT25 and BT147 intracranial xenografts for IL-33 identifies a 33 kDa immunoreactive band in BT147. *N* = 3 experiments. **f** Representative images of IHC for IL-33 (brown) in BT25 and BT147 intracranial xenografts. Sections were counterstained with hematoxylin (blue; *N* = 5). Scale bar, 50 µm. **g** Representative Multiplex IF image of h-nucleolin (red), IL-33 (blue), and Iba1 (green) in BT147 xenograft (*N* = 3). Scale bar, 30 µm. See also Supplementary Fig. 1. Source data are available as a Source data file.

One potential reason that many promising in vitro studies fail at the preclinical/clinical level with respect to brain tumor therapies is that the tumor microenvironment, and in particular the immune component, plays a direct role in contributing to therapeutic resistance. To begin to identify and assess the role of secreted factors present within the inflammatory brain tumor environment, we utilized multiplex cytokine/chemokine bead arrays on interstitial fluid containing the secretome from the tumor microenvironment (glioma and host-derived interstitial fluid) from two distinct patient-derived BTIC orthotopic animal models (BT25 and BT147) that differ in abundance of TAM. BT147 model is a highly invasive, rapidly growing tumor with significant infiltration of Iba1^+^ TAMs, while BT25 forms poorly invasive, immunologically quiescent tumors (Fig. 1a, b). Assessment of the interstitial fluid from these phenotypically diverse tumors identified both common and differentially secreted factors (Fig. 1c) that were identified as glioma-derived (left panel) or host-derived (right panel) proteins based on human or mouse-specific antibodies. The multiplex analysis revealed abundant expression of human IL-33, CXCL10 (IP10), CCL2 (MCP1), CXCL8 (IL-8), CCL7 (MCP3), TGFα, KITLG (SCF), and mouse IL-4 and CXCL10 in the secretome of the inflammatory BT147 xenografts, as compared to BT25 xenografts (Fig. 1c, d). In addition, there was a decrease in a number of cytokines that included hVEGF, hLIF, hTSLP, hPDGFAA, mIL10, mIL1α, mIL9, and mCCL3 (MIP-1α; Fig. 1c). All other secreted factors were either not changed or below detectable levels. Importantly, a subset of these human-derived cytokines that included CXCL10, IL-33, CXCL8, KITLG, CCL2, and TGFα were not secreted or secreted at very low levels in vitro by BT147 cells (Supplementary Fig. 1a), suggesting that the increased secretion was the result of a glioma–host cell interaction within the brain.

We observed that IL-33 was one of the most abundantly secreted factors, and based on its ability to act on a range of innate and adaptive immune cells^51,63,64^, we prioritized it for further investigation. Western blot analysis of the interstitial fluid from BT147 xenografts confirmed the presence of a 33-kDa IL-33-immunoreactive band (Fig. 1e) and immunohistochemical assessment validated the presence of IL-33 in vivo, including its localization within the nucleus (h-nucleolin; Fig. 1f). Using multiplex immunofluorescence (IF) for IL-33, the macrophage/microglial marker Iba1, and h-nucleolin (Fig. 1g), we confirmed that the major cellular source of IL-33 was the human glioma cells (cells positive for h-nucleolin) with a small number of Olig-2 expressing oligodendrocytes in both normal and tumor-bearing animals (Supplementary Fig. 1b).

### IL-33 glioma associate with TAM infiltration

**Fig. 2:**
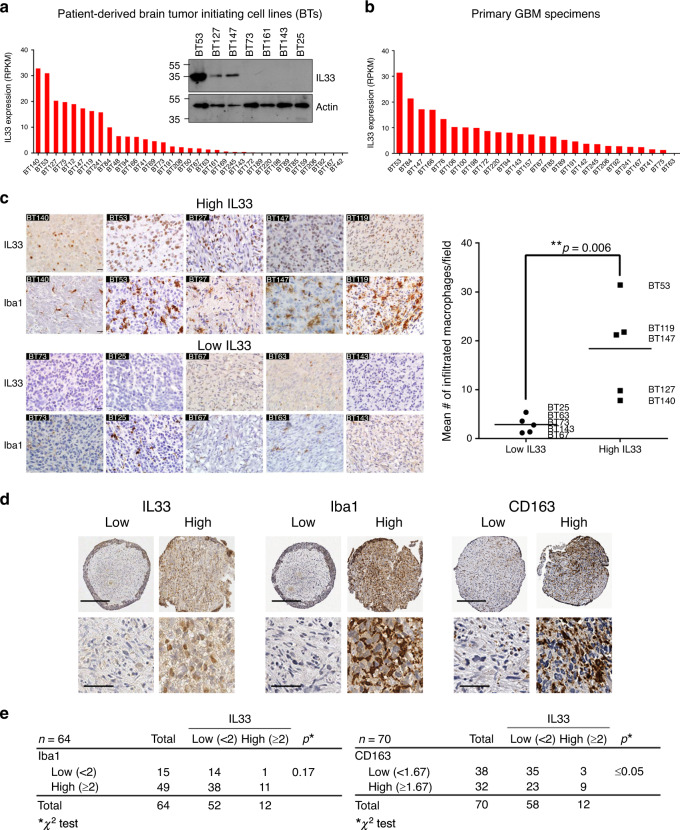
Human glioblastoma expressing IL-33 correlate with TAM density. **a** RNA-seq was performed on 35 patient-derived BTICs and 26 primary GBM specimens. Graph shows RNA expression (per million mapped reads; RPKM) of IL-33 in the panel of patient-derived BTIC lines. Insert shows a western blot for IL-33 (upper panel) in a subset of the patient-derived BTICs. Actin was used as a loading control (lower panel). **b** Graph shows RNA expression (RPKM) of IL-33 in the corresponding primary GBM specimens from which the BTICs were isolated. **c** IHC of IL-33 (rows 1 and 3; brown) and Iba1 (rows 2 and 4; brown) expression in intracranial xenografts derived from five BTIC lines with high IL-33 expression (BT140, BT53, BT127, BT147, and BT119; upper 2 rows) and five BTIC lines with low/absent IL-33 expression (BT73, BT25 BT67, BT63, and BT143; lower two rows; *N* = 3). Graph shows quantitation of the number of Iba1^+^ macrophage per field of view (right panel). Results are expressed as the mean of Iba1^+^ macrophage in five independent fields of view (3 mice/group) per BTIC; **p = 0.006 by two-sided unpaired Student’s *t*-test. Scale bar, 20 µm. **d** IHC of a tissue microarray (TMA) containing 70 glioblastoma patient specimens was assessed for IL-33 (left panel; brown), Iba1 (middle panel; brown), and CD163 (right panel; brown). TMA was counterstained using hematoxylin (blue). Shown are representative images of high and low expression of IL-33, Iba1, and CD163. Scale bar, 200 µm (upper panels) and 50 µm (lower panels). **e** TMA was scored and assessed for an association between IL-33 expression and Iba1 or CD163 expression. The *p*-value was based on the chi-squared (*χ*^2^) test. See also Supplementary Fig. 2. Source data are available as a Source data file.

To determine the prevalence and levels of IL-33 expression in a larger set of BTICs and primary GBM specimens, RNA-seq was performed on a panel of 35 BTIC cultures and 26 primary GBM specimens^65^. Of this cohort of samples, >50% of samples expressed IL-33 (Fig. 2a, b) with 8/35 BTICs (22.9%) and 8/26 (30.7%) GBM specimens expressing high levels of IL-33 (≥10 Reads Per Kilobase of transcript per Million mapped reads (RPKM)). Western blot analysis confirmed IL-33 protein in the BTICs (Fig. 2a inset), and gene expression established a significant correlation between IL-33 in the BTIC and the matched primary GBM specimen from which they were derived (21 matched pairs; *r* = 0.60; *p* = 0.0043; see Supplementary Fig. 2a). To determine if IL-33 correlates with TAM infiltration, we selected five IL-33 high (Fig. 2c, upper 2 rows) and five IL-33 low/absent (Fig. 2c, lower 2 rows) BTICs based on the RNA-seq data, and implanted them into the brains of immunocompromised (SCID) mice. Mice were sacrificed when they displayed signs of morbidity. Immunostaining confirmed IL-33 protein, and as predicted by the initial screen (Fig. 1), showed that high IL-33^+^ BITC xenografts contained a significantly higher density of Iba1^+^ TAMs (*p* ≤ 0.01, Fig. 2c graph). In addition, multiplex cytokine analysis of the interstitial fluid isolated from a second pair of inflammatory (BT53) and noninflammatory (BT73) xenografts, showed significantly higher cytokine levels of IL-33, CCL2, and CXCL10 in the inflammatory xenograft, (Supplementary Fig. 2b). To extend these results, we used an independent cohort of GBM specimens (tissue microarray (TMA) containing 70 patient specimens) and found that 12/64 (18.8%) had high IL-33 expression that was predominately localized within the nucleus of glioma cells (Fig. 2d, left panel). When this cohort of patient specimens was also assessed for Iba1, a trend toward a significant correlation between IL-33 and Iba1 expression was observed (*p* = 0.17; Fig. 2d, middle panel and Fig. 2e, left panel), suggesting that IL-33 may be one factor by which TAM are recruited. Moreover, when assessed for the presence of a pro-tumorigenic M2 macrophage as indicated by CD163, a significant correlation was established between IL-33^+^ patient tumors and the presence of M2 macrophage phenotype (*p* ≤ 0.05; Fig. 2d, e, right panel) supporting the posit that glioma-derived IL-33 recruits pro-tumorigenic macrophage. This is consistent with reports showing both macrophage and microglia express the IL-33-receptor ST2 (refs. ^66,67^) and that IL-33 can polarize macrophage to an M2 phenotype^68,69^. In addition, RNA-seq and western blot analysis of the glioma cells show little or no detectable expression of ST2 (Supplementary Fig. 2c, d) further supporting that the secretion of IL-33 by the glioma cells is acting on other components of the tumor microenvironment. Moreover, while some correlative data exists for distinct mutational landscapes influencing differences in cellular environment, analysis of the TCGA database validating elevated levels of IL-33 in the GBM patient cohort (Supplementary Fig. 2e) did not find a correlation with any obvious genetic descriptor, including molecular subtype, MGMT methylation status, or common mutations (e.g., p53, PTEN EGFRVIII). Likewise, although recent data suggests that male-specific expression of IL-33 regulates sex-dimorphic susceptibility to experimental autoimmune encephalomyelitis^70^, no sex differences regarding the presence of IL-33 in human glioma were found, and expression in both sexes correlates with worse overall survival.

### Secreted and nuclear IL-33 accelerates tumor progression

**Fig. 3:**
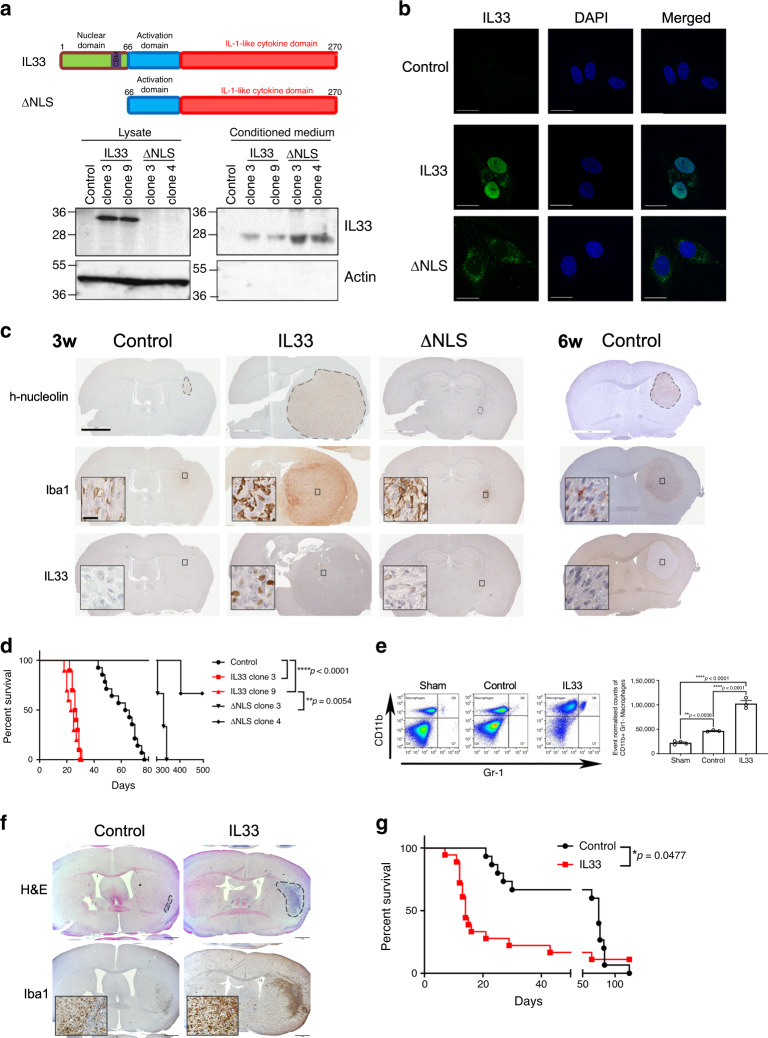
Nuclear IL-33 is required for tumor growth and macrophage infiltration. **a** Diagram of full-length and mutant IL-33 constructs. Full-length IL-33 consists of a nuclear localization domain (green), chromatin-binding motif (CBM; purple), an activation domain (blue), and an IL-1 like cytokine domain (red). Deletion mutant (ΔNLS) lacks the nuclear domain. IL-33 western blot of U87-IL-33 (clones 3 and 9), ΔNLS (clones 3 and 4), or pcDNA3.1 vector (control) detect full-length IL-33 (33 kDa) in cell lysates and a 25 kDa secreted form in the conditioned medium. Actin was used as a loading control. *N* = 3 experiments. **b** Immunofluorescence for nuclear IL-33 (green) in control, IL-33, or ΔNLS transfected U87 cells 24 h after treatment with leptomycin B (50 nM). Scale bar, 20 µm. **c** Mice implanted with pcDNA (control), IL-33, and ΔNLS-expressing U87 were sacrificed at 3 weeks, when IL-33 mice became symptomatic. Second cohort was sacrificed at 6 weeks postinjection. Shown are representative images of IHC (brown) for h-nucleolin, Iba1, and IL-33. Experiment was repeated five times (*N* = 5 mice per group/experiment). Sections were counterstained with hematoxylin (blue). Scale bar, 2 mm; inset scale bar, 25 µm. **d** Kaplan–Meier survival curve of animals bearing U87-control (*N* = 14), IL-33 (clone 3, *N* = 10; clone 9, *N* = 10), or ΔNLS (clone 3, *N* = 3; clone 4, *N* = 3) tumors from three independent experiments. The *p*-value was calculated using log-rank Mantel–Cox test. Control vs. IL-33 clones 3 or 9, ****p ≤ 0.0001; IL-33 clone 9 vs. ΔNLS clone 3, **p = 0.0054. **e** Quantification of CD11b^+^Gr1^−^ macrophage in control (*N* = 3) and IL-33 (*N* = 3) tumors using flow cytometry. PBS-injected mice were used as sham control (*N* = 4). Graph shows the normalized counts of CD11b^+^Gr1^−^ cells per million total events. Data are mean ± SEM; **p ≤ 0.01, ****p ≤ 0.0001 by one-way ANOVA with Tukey’s post hoc test. **f** Syngeneic K1492 glioma cells expressing IL-33 or empty vector (control) were allowed to grow for 10 days when IL-33^+^ mice became symptomatic. Representative H&E and Iba1 (brown) IHC images are shown. Experiment was repeated five times (*N* = 5 per group/experiment). Sections were counterstained with hematoxylin (blue). Scale bar,  1 mm; inset scale bar, 50 µm. **g** Kaplan–Meier survival curve of animals bearing control (*N* = 15) or IL-33 (*N* = 18) tumors from three independent experiments. The *p*-value was calculated using log-rank Mantel–Cox test (control vs. IL-33, *p* = 0.0477). See also Supplementary Fig. 3. Source data are available as a Source data file.

Since IL-33 functions as a soluble cytokine through activation of the ST2 receptor complex and as a nuclear factor with transcriptional regulatory properties^71^, we examined the effects of secreted and/or nuclear IL-33 on TAM recruitment and tumor growth. To accomplish this, we generated plasmid vectors containing either the 270 amino acid full-length human IL-33 or a mutant form of IL-33, in which the nuclear localization signal (amino acids 1–65) was deleted (IL-33 ΔNLS; Fig. 3a)^49^. Constructs were then expressed in two genetically distinct human glioma cell lines that lack IL-33 expression, and are extensively characterized for their tumor growth and associated features in vivo (U87, U251N)^72–74^. Western blot analysis confirmed the expression of both the full length (33 kDa band) and the IL-33 ΔNLS (25 kDa band; Fig. 3a and Supplementary Fig. 3a), and revealed that in the absence of nuclear sequestration (ΔNLS), IL-33 is not retained within the cell (cell lysate), but instead is secreted at high levels into the media (conditioned media; CM), as observed by others^75^. The secretion of the smaller isoform is consistent with the observation that diverse proteases cleave IL-33 into shorter bioactive forms that can still bind ST2 (refs. ^76,77^). Glioma cells expressing the pcDNA vector were used as a control. We validated that the full-length IL-33, and not the IL-33 ΔNLS, resulted in nuclear accumulation of IL-33 that was further enhanced when treated with the nuclear transport inhibitor leptomycin B (Fig. 3b and Supplementary Fig. 3b), a result that supports the shuttling of IL-33 between the cytoplasm and nucleus, similar to other known transcriptional regulators^78^. Next, to assay the in vivo effects of glioma-derived IL-33, tumor cells expressing IL-33, or the nuclear localization-deficient IL-33 were implanted into the brains of SCID mice and monitored for tumor burden. Three weeks after implantation, when the mice bearing IL-33^+^ tumors became symptomatic, all mice were sacrificed and the brains were removed, fixed in formalin, and paraffin-embedded. Whole brain sections were immunostained with antibodies to IL-33, Iba1, and h-nucleolin (Fig. 3c and Supplementary Fig. 3c). Similarly, the second cohort of mice, bearing control (pcDNA) or ΔNLS IL-33 glioma cells, were monitored until the control mice became symptomatic, ~6 weeks postinjection, at which point all remaining mice were sacrificed and prepared for IHC. As shown in Fig. 3c and Supplementary Fig. 3c, ectopic expression of the full-length IL-33 in both glioma lines resulted in a dramatic increase in tumor growth with a concomitant increase in Iba1^+^ TAMs and a significant decrease in the overall survival (*p* < 0.0001; Fig. 3d and Supplementary Fig. 3d). Flow cytometry confirmed a significant increase in CD11b^+^/Gr1^−^ macrophage in IL-33^+^ tumors as compared to sham-injected controls or vector control tumor-bearing mice (Fig. 3e). In addition, loss of nuclear localization abrogates IL-33-induced tumor growth and significantly prolonged survival (Fig. 3d and Supplementary Fig. 3d). Unexpectedly, although small tumors were detected at 3 weeks in the ΔNLS xenografts (Fig. 3c and Supplementary Fig. 3c), no detectable ΔNLS tumors were present at 6 weeks postinjection in either genetically diverse glioma suggesting that these tumors either underwent cell death and/or were cleared by the innate immune system. In addition, and as observed in vitro, IL-33 was predominantly localized within the nucleus of the glioma cells, while IL-33 was undetectable in the nucleus of the ΔNLS glioma cells in vivo (Fig. 3c and Supplementary Fig. 3c). Moreover, as no significant difference in cell proliferation was detected in IL-33 or ΔNLS IL-33 in vitro (Supplementary Fig. 3e), these data further support that the in vivo effects are the result of glioma–host interactions. In addition, treatment with recombinant IL-33 (rIL-33) or CM from glioma cells expressing IL-33 or ΔNLS IL-33 were capable of recruiting BMDM in vitro (Supplementary Fig. 3f, g). These data support the idea that the secretion of IL-33 is sufficient to recruit TAM, but that the nuclear function of IL-33 is required to facilitate a pro-tumorigenic macrophage phenotype.

Since the above studies rely on human xenograft models that require the use of immune incompetent animals, and depend on the ability of human factors to target murine host cells, we performed a similar line of investigation in a murine syngeneic model. Assessment of glioma cell lines generated from the NF1^*−/+*^p53^*−/+*^ murine glioma model^79^ showed a range of IL-33 expression with at least one line endogenously expressing IL-33 (K1491), while other lines including K1492 showed no detectable expression (Supplementary Fig. 3h). To evaluate the function of IL-33 in this immune competent model, we generated a variant line of K1492 engineered to express murine IL-33. As in the case of the human glioma cells, the murine glioma cells also express IL-33 in two locales, the nucleus and secreted (Supplementary Fig. 3h). Importantly, and similar to the human xenografts, the mere expression of IL-33 resulted in enhanced tumor growth, increased infiltrating TAMs, and decreased the overall survival (Fig. 3f, g). Moreover, although the tumor burden was substantially increased in the IL-33-expressing glioma compared to the IL-33-negative tumors, the actual size of the tumor suggests that the clinical criteria for endpoint were not reached exclusively due to tumor burden. Rather, these data suggest that other contributing factors, including the establishment of a highly inflammatory cytokine environment contributed, supporting a role for IL-33 in modulating the TME.

### Nuclear IL-33 contributes to the pro-tumorigenic milieu

**Fig. 4:**
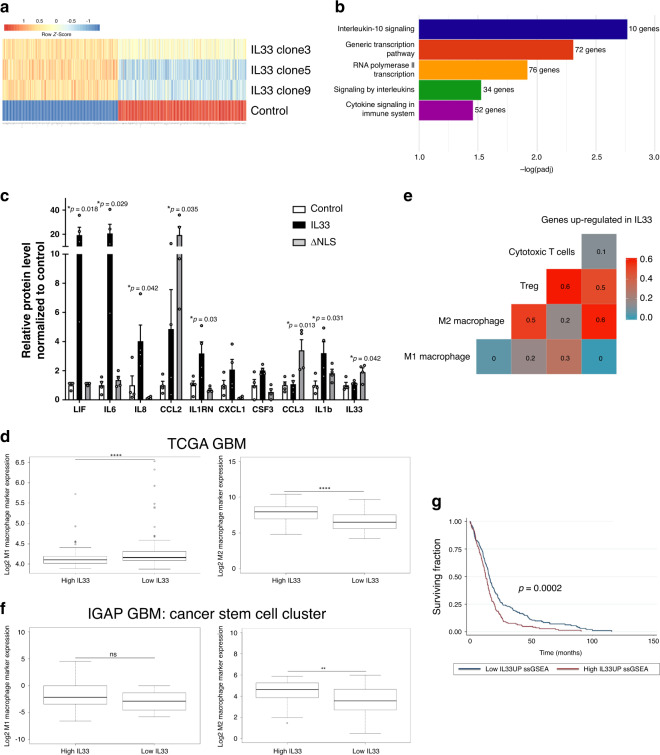
Nuclear IL-33 mediates release of inflammatory cytokines in glioma cells. **a** Heatmaps visualize *z*-scores from Affymetrix microarray data of differentially expressed genes in three independent IL-33-expressing U87 human glioma clones (IL-33; clones 3, 5, and 9), and one IL-33^−^ (pcDNA; control) cell line (upregulated genes are red; downregulated genes are blue). **b** Functional annotation using DAVID shows overrepresentation of Gene Ontology terms in genes upregulated in IL-33^+^ cells as compared to control (https://david.ncifcrf.gov). *X*-axis shows adjusted *p*-values corrected for multiple testing for each Gene Ontology term. **c** Cytokine levels in the conditioned media (CM) of IL-33^−^ (control), IL-33^+^ (IL-33), or ΔNLS U87 cells measured using a human 65-plex Luminex array. Results are expressed as mean ± SEM for each protein after normalization to control. *N* = 4 independent experiments. *p ≤ 0.05 compared to control by one-way ANOVA with Tukey’s post hoc test. Control vs. IL-33 (LIF, *p* = 0.018; IL-6, *p* = 0.029; IL-8, *p* = 0.042; IL-1RN, *p* = 0.03; and IL-1b *p* = 0.031); control vs. ΔNLS (CCL2, *p* = 0.035; CCL3, *p* = 0.013; and IL-33, *p* = 0.042). **d** Box plots show the quantification of M1 and M2 macrophage markers in high versus low *IL-33*-expressing TCGA GBM patient samples. High *IL-33* is defined as the top 25^th^ percentile of samples in terms of *IL-33* expression and low *IL-33* is defined as the bottom 25^th^ percentile. M1 marker expression in high *IL-33* expressing tumors: minima: 3.90; maxima: 5.72; centre: 4.11; first quartile: 4.02; third quartile: 4.20, M1 marker expression in low *IL-33* expressing tumors: minima: 3.88; maxima: 6.53; centre: 4.17; first quartile: 4.09; third quartile: 4.31, M2 marker expression in high *IL-33* expressing patients: minima: 4.78; maxima: 10.34; centre: 7.95; first quartile: 7.16; third quartile: 8.49 and M2 marker expression in low *IL-33* expressing tumors: minima: 4.54; maxima: 9.42; centre: 6.71; first quartile: 5.91; third quartile: 7.73. **e** Correlation matrix shows a correlation between *IL-33* expression and immune cells (summarized using ssGSEA scores; see “Methods” section for markers used) in TCGA GBM patient cohort. **f** Box plots show the quantification of M1 and M2 macrophage markers in high (top 25^th^ percentile) versus low (bottom 25^th^ percentile) *IL33*-expressing cancer stem cells from the IVY GBM Atlas project (IGAP). M1 marker expression in high *IL-33* expressing tumors: minima: −6.56; maxima: 4.50; centre: −2.16; first quartile: −3.47; third quartile: 0, M1 marker expression in low *IL-33* expressing samples: minima: −5.75; maxima: 0; centre: −2.87; first quartile: −4.64; third quartile: −1.02, M2 marker expression in high *IL-33* expressing samples: minima: 1.47; maxima: 5.90; centre: 4.63; first quartile: 3.73; third quartile: 5.26 and M2 marker expression in low *IL-33* expressing samples: minima: 0.52; maxima: 5.98; centre: 3.56; first quartile: 2.61; third quartile: 4.71. **g** Unadjusted Kaplan–Meier curves showing the association between *IL-33* expression and survival in the TCGA GBM patient cohort. The *p*-value was calculated using log-rank Mantel–Cox test. In all boxplots, the lower bound of the whisker represents the minimum value (minima); the upper bound of the whisker represents the maximum values (maxima), centre of the box represents the median value, lower and upper bounds of the box represent the first and the third quartiles, respectively; outliers are represented by hollow dots. See also Supplementary Fig. 4. Source data are available as a Source data file.

Since IL-33 is known to associate with chromatin and regulate transcriptional activity^49^, and that nuclear expression of IL-33 increases glioma progression (Fig. 3), we performed global gene expression analysis on three independent IL-33 ectopically expressing glioma cell clones as compared to control (empty vector) cells (Fig. 4a). Thresholds for differentially expressed genes were the fold change of greater than or equal to 2.0 with a false detection rate (FDR) of 0.01. Using these parameters, 340 genes were induced by the ectopic expression of IL-33 and an additional 377 genes were downregulated. Gene Ontology (GO) terms overrepresented in the genes induced by IL-33 include three major clusters that associate with cytokine activity and inflammation (Fig. 4b). Among the top 50 genes induced by IL-33 in at least two clones were the inflammatory genes *IL1β*, *IL6*, *G-CSF*, *LIF*, *CXCL3*, *CXCL8*, *IL1RA*, *MIP*, and *CCL2* (Supplementary Fig. 4a). To validate the modulation of genes by ectopic IL-33 in our expression data and to determine if nuclear IL-33 is involved in the regulation of these cytokines, CM from control (pcDNA), IL-33, and IL-33-ΔNLS-expressing cells, were analyzed by multiplex cytokine/chemokine analysis. We confirmed the expression and secretion of a large number of inflammatory cytokines in IL-33^+^ glioma cells (Fig. 4c) and, with the exception of CCL2, indicate regulation of these genes by nuclear IL-33. Furthermore, analysis of gene expression data from The Cancer Genome Atlas (TCGA) and our own cohort of GBMs (TFRI GBM^65^), validated a positive correlation between IL-33 mRNA expression and pro-tumorigenic M2 macrophage markers, with a concomitant negative correlation with M1 macrophage markers (Fig. 4d and Supplementary Fig. 4b). Upon summarization using single-sample gene set enrichment analysis (ssGSEA), genes induced by IL-33 showed a positive correlation with M2 macrophage markers and T-regulatory cell markers in the TCGA GBM cohort (see correlation matrix Fig. 4e). We also observed elevated *IL-33* expression in the cancer stem cell cluster when compared to other (non-stem cell) microdissected anatomical structures from the Ivy Glioblastoma Atlas Project (Supplementary Fig. 4c). Restricting our analysis to either the stem cell cluster or the non-stem cell anatomical structures alone, a positive correlation between *IL-33* expression and M2 macrophage markers was observed in both cases (Fig. 4f and Supplementary Fig. 4d). These data corroborate our previous observations that IL-33 promotes an immunosuppressive phenotype in GBM. Lastly, and notably, when ssGSEA scores summarizing the expression of IL-33 upregulated genes were stratified, high ssGSEA scores were associated with poor disease-specific survival (*p* = 0.0002; Fig. 4g), as would be predicted by the pro-tumorigenic nature of an IL-33-driven microenvironment.

**Fig. 5:**
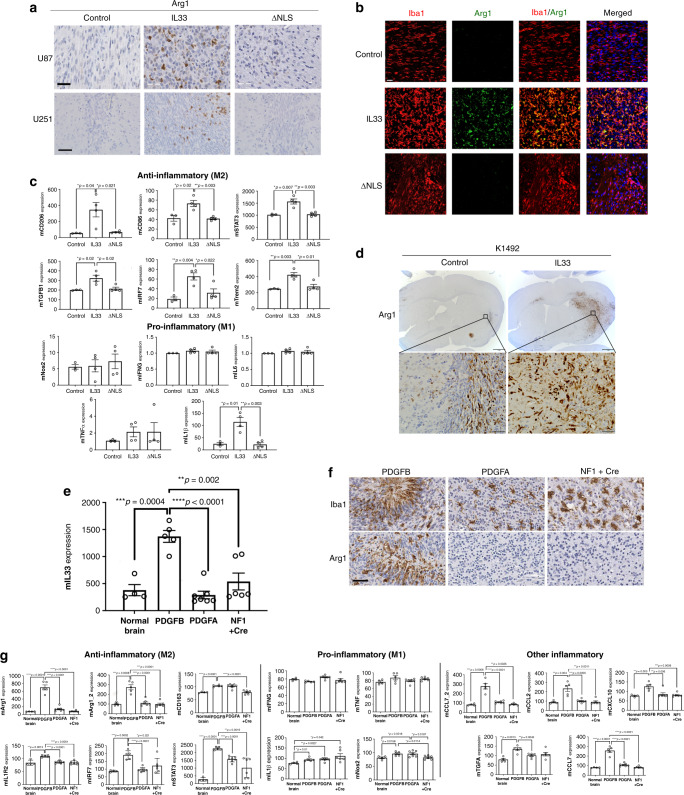
Nuclear IL-33 is required to recruit M2 pro-tumorigenic macrophage. **a** Representative IHC images of xenografts from IL-33^−^ (pcDNA; control), IL-33^+^ (IL-33), or ΔNLS U87 or U251 assessed for arginase 1 (Arg1; brown) and counterstained with hematoxylin (blue; *N* = 5). Scale bar: 50 µm. **b** Immunofluorescent images double stained for Arg1 (green) and Iba1 (red) in xenografts from IL-33^−^ (pcDNA; control), IL-33^+^ (IL-33) or ΔNLS U87 tumor-bearing mice (*N* = 3). Scale bar: 30 µm. **c** Mouse inflammatory gene expression detected by Nanostring nCounter platform. Graphs show expression of inflammatory mediators in xenografts from IL-33^−^ (control; *N* = 3), IL-33^+^ (IL-33; *N* = 4), or ΔNLS (*N* = 4) U87 tumor-bearing mice, 1 week after tumor implantation. Data are mean ± SEM; *p ≤ 0.05, **p ≤ 0.01, ***p ≤ 0.001, by one-way ANOVA with Tukey’s post hoc test. **d** IHC images for Arg1 (brown) in K1492-control and IL-33-expressing glioma. Scale bar: 1 mm (upper panel); 50 µm (lower panel). **e** Illumina beadchip expression array data from normal mouse brain (control; *N* = 4) and tumor tissue from PDGFB (*N* = 5), PDGFA (*N* = 7), or shNF-1/Cre-induced (*N* = 6) RCAS glioma. Data are mean ± SEM. **p ≤ 0.01, ***p ≤ 0.001, ****p ≤ 0.0001 by one-way ANOVA with Tukey’s post hoc test. **f** Representative IHC (brown) images for Iba1 and Arg1 in PDGFB, PDGFA, and shNF-1/Cre-driven glioma. Sections counterstained with hematoxylin (blue). Scale bar: 50 µm. **g** Illumina beadchip expression array of PDGFB, PDGFA, and shNF-1/Cre-driven glioma. Data are mean ± SEM; *p ≤ 0.05, **p ≤ 0.01, ***p ≤ 0.001, ****p ≤ 0.0001 by one-way ANOVA with Tukey’s post hoc test. See also Supplementary Fig. 5. Source data are available as a Source data file.

**Fig. 6:**
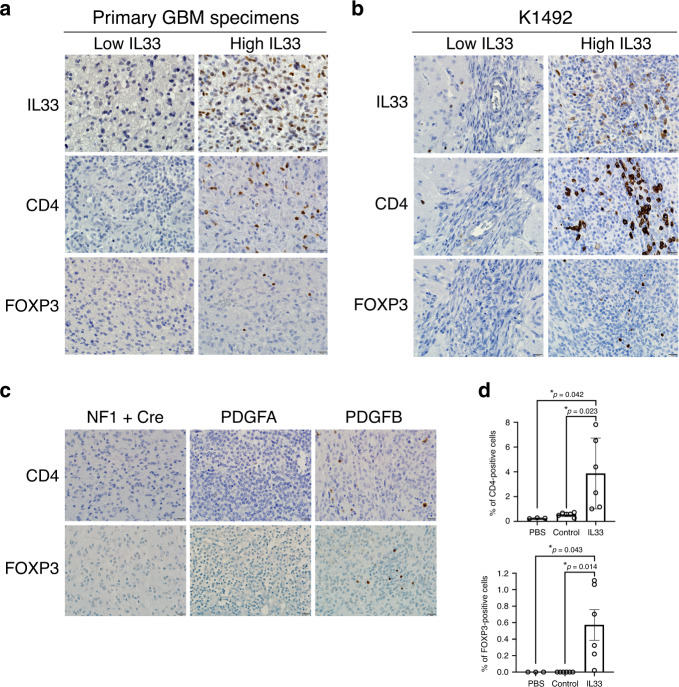
FOXP3^+^ T cells in IL-33-expressing tumors. Shown are representative IHC (brown) for IL-33, CD4, and FOXP3 in **a** primary GBM specimens (CD4 *N* = 5; FOXP3 *N* = 5), **b** syngeneic K1492-control and IL-33^+^ mouse glioma (CD4 *N* = 6; FOXP3 *N* = 6), and **c** PDGFB, PDGFA, and shNF-1/Cre-driven glioma (CD4 *N* = 3, FOXP3 *N* = 3). Sections were counterstained with hematoxylin (blue). Scale bars: 20 µm. **d** Graph shows the quantitation of CD4^+^ (upper panel) and FOXP3^+^ (lower panel) cells present in K1492-control (*N* = 6) or IL-33^+^ (*N* = 6) xenografts. Mice injected with PBS were used as sham controls (*N* = 3). Results are expressed as the mean ± SEM of the percentage of positive cells/whole brain section (K1492-control and IL-33^+^ 6 mice/group; sham control 3 mice/group); *p ≤ 0.05 by one-way ANOVA with Tukey’s post hoc test. Source data are available as a Source data file.

Consistent with the primary patient data (Figs. 2 and 4d–f), the expression of the glioma-derived IL-33 in vivo resulted in an increase in the abundance of arginase 1 (Arg1)-positive cells, indicative of a pro-tumorigenic macrophage population (Fig. 5a, b), while the Iba1^+^ cells in the absence of the nuclear localization (ΔNLS) did not express Arg1 implying that the macrophage recruited by glioma cells expressing this mutant have an M1 anti-tumorigenic phenotype that may mediate tumor cell clearance and regression of the ΔNLS tumors in vivo (Fig. 3c and Supplementary Fig. 3c). Gene expression analysis using the nCounter Mouse v2 Inflammation Panel validated a significant increase in a number of anti-inflammatory cytokines in the IL-33^+^ xenografts that were absent in the ΔNLS xenografts (Fig. 5c and Supplementary Fig. 5). Similarly, evaluation of the NF^*−/+*^p53^*−/+*^ K1492 syngeneic model found that expression of IL-33 also resulted in increased infiltration of Arg1^+^ TAMs within the tumor (Fig. 5d). Moreover, and consistent with IL-33 driving this pro-tumorigenic environment, assessment of other genetically engineered immunocompetent glioma models identified elevated levels of IL-33 in the neonatal *N/tv-a;Cdkn2a*^−^^*/−*^*;Ptenfl/fl mice* (Xfm) PDGFB-driven murine glioma model^80^ (Fig. 5e), with a corresponding infiltration of Arg1^+^ TAMs (Fig. 5f). This observation was in contrast to the PDGFA or shNF1-driven murine glioma models, where both IL-33 and Arg1^+^ microglia/macrophage were detected at significantly lower levels (Fig. 5e, f). In addition, microarray analysis revealed that the PDGFB-driven glioma had a significant increase in other anti-inflammatory (M2) mediators, including IL1R2, IRF7, and STAT3 (Fig. 5g). Furthermore, the use of these immunocompetent models allowed the examination of other immune cell populations, and since IL-33 has been shown to play a role in T cell activation^81–83^, we first examined the T cell repertoire in these tumors. While it is generally thought that there are very few T cells in human glioma, we observed a positive correlation with T-regulatory cell markers in the TCGA GBM cohort (see correlation matrix Fig. 4e). Immunohistochemical evaluation further validated the presence of FOXP3^+^ T-regulatory cells within the IL-33-expressing *NF1*^*−/+*^*p53*^*−/+*^ K1492, the *N/tv-a;Cdkn2a*^*−/−*^*;Ptenfl/fl mice* (Xfm) PDGFB-driven glioma, and primary GBM specimens albeit at fairly low but reproducible levels (Fig. 6a–d). Reevaluation of the TCGA data verified a positive correlation with the expression of IL-33 and T-regulatory markers, and strengthened the linkage to human glioma biology; data consistent with a study showing ST2^+^ T-regulatory cells are mobilized in response to IL-33 (ref. ^84^). Together these data suggest that IL-33 expressing glioma cells establish a pro-tumorigenic yet anti-inflammatory environment that mediates a dramatic increase in tumorigenesis.

### Functional role for IL-33 in brain tumors

**Fig. 7:**
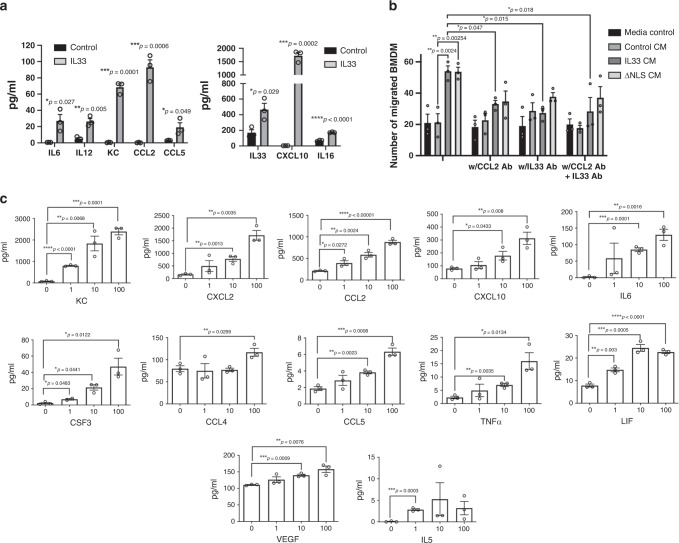
A functional role for an IL-33-orchestrated tumor microenvironment. **a** Cytokine levels in the tumor interstitial fluid (TIF) from IL-33^−^ (control) or IL-33^+^ (IL-33) K1492 cells. *N* = 3 independent experiments expressed as the mean ± SEM; *p ≤ 0.05, **p ≤ 0.01, *** *p* ≤ 0.001, ****p ≤ 0.0001 by two-tailed unpaired Student’s *t*-test. **b** Macrophage were harvested from the femur of mice, differentiated for 7 days and plated in transwells with conditioned medium (CM) from control, IL-33, or ΔNLS-expressing U87 in the presence or absence of CCL2, and/or IL-33 neutralizing antibodies. Graph shows the number of BMDM that migrate in 4 h. Data are mean of three replicates ± SEM. *N* = 3 experiments with comparable results; *p ≤ 0.05, **p ≤ 0.01 by one-way ANOVA Dunnett’s post hoc test. **c** BMDM were treated with rIL-33 (0–100 pg/ml) and cytokine levels in the CM were measured by Luminex. Results from three independent experiments are expressed as the mean ± SEM; *p ≤ 0.05, **p ≤ 0.01, ***p ≤ 0.001, ****p ≤ 0.0001 compared to no treatment by one-way ANOVA with Tukey’s post hoc test. See also Supplementary Fig. 6. Source data are available as a Source data file.

Since the IL-33 environment enriches for the recruitment of BMDM, we initially investigated the direct impact of IL-33 (Fig. 7). To do this, we isolated and purified (>97% purity, see Supplementary Fig. 6a) BMDM from C57BL/6 mice, treated with rIL-33, and assessed the CM using multiplex cytokine/chemokine array. Exposure to rIL-33 resulted in a dose-dependent increase in a number of cytokines (KC, CXCL2, CCL2, CXCL10, IL-6, CSF3, CCL5, TNFα, and LIF; Fig. 7a, c). In addition, when human fetal microglia were exposed to the rIL-33, a similar increase in cytokine/chemokine production was observed (Supplementary Fig. 6b), suggesting that IL-33 may have a direct impact on both recruited and resident macrophage populations. Moreover, these data suggest that IL-33 regulates the secretion of a larger number of cytokines including several known to modulate the recruitment of innate immune cells^51,85^. Next, to functionally assess these IL-33-regulated factors, BMDM were exposed to glioma-derived CM from IL-33^+^, IL-33-ΔNLS, and IL-33^−^ glioma cells. CM from the IL-33^+^ cells resulted in an increase in migration and proliferation of the BMDM (Fig. 7b and Supplementary Fig. 6c), a result in contrast to CM from glioma cells that did not express IL-33. In addition, CM from ΔNLS-expressing glioma cells showed equal impact on the migratory behavior of the BMDM (Fig. 7b). Importantly, this increase in migration in both IL-33 and ΔNLS CM could be reduced by neutralizing antibodies to IL-33 or CCL2 (Fig. 7b), a chemokine upregulated by rIL-33 similar to upregulation of chemokines in IL-33-expressing glioma cells or present in the tumor interstitial fluid (TIF) of IL-33-expressing tumors (Figs. 1d, 4c and 7c). Finally, as mentioned, rIL-33 alone was sufficient to mediate this migratory behavior (Supplementary Fig. 3g), thus supporting both direct and indirect (e.g., CCL2 mediated) effects on the BMDM.

**Fig. 8:**
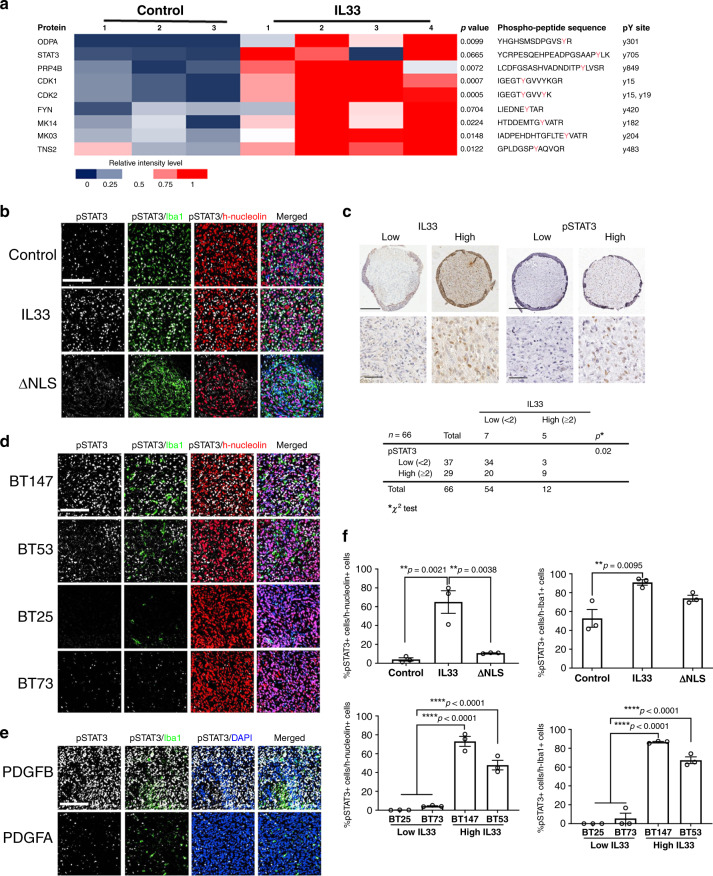
Phosphoproteomic characterization of IL-33-expressing xenograft. **a** Comprehensive protein-pY analysis was performed on intracranial tumors from mice bearing IL-33^−^ (control; *N* = 3) or IL-33^+^ (IL-33; *N* = 4) U87-tumors. Heat map shows pY-peptides enriched in the IL-33^+^ tumors. The *p*-values were obtained by two-sided unpaired Student’s *t*-test. **b** Shown are representative immunofluorescence images of pSTAT3 (white), Iba1 (green), and human nucleolin (red) in xenografts from IL-33^−^ (control), IL-33^+^ (IL-33), or ΔNLS U87 tumor-bearing mice. Scale bar: 30 µm. **c** IHC of a tissue microarray (TMA) containing 66 glioblastoma patient specimens was assessed for IL-33 (left panel; brown) and pSTAT3 (right panel; brown). TMA was counterstained using hematoxylin (blue). Shown are representative images of IL-33 high and low expressing tumors. Scale bar, 200 µm (upper panels) and 50 µm (lower panels). TMA was scored and assessed for an association between IL-33 and pSTAT3 expression. The *p*-value was based on the chi-squared (*χ*^2^) test. **d** Shown are representative images of Multiplex IF of pSTAT3 (white), Iba1 (green), and human nucleolin (red) in BT147, BT53, BT25, and BT73 xenografts. Scale bar 30 µm. **e** Shown are representative images of Multiplex IF of pSTAT3 (white), Iba1 (green), and DAPI (blue) in PDGFB, PDGFA, and shNF1-driven mouse glioma. Scale bar: 30 µm. **f** Quantification shows the percentage of pSTAT3^+^ cells as compared to the total number of human nucleolin or Iba1^+^ cells. Data are mean ± SEM from six independent fields of view from 3 mice/group; **p ≤ 0.01, ****p ≤ 0.0001 by one-way ANOVA with Tukey’s post hoc test. Source data are available as a Source data file.

In order to further understand how glioma-derived IL-33 is orchestrating the changes within the TME and based on the significant increase of anti-inflammatory mediators (Fig. 5), including a number of signaling molecules observed in IL-33^+^ tumors, we performed phosphoproteomic analysis using mass spectrometry on the brains of tumor-bearing mice. Tyrosine-specific phosphoproteomic analysis of IL-33^+^ tumors showed an increase in a number of phospho-peptides derived from STAT3, CDK1, CDK2, FYN, MAPK14, and MAPK3 (Fig. 8a), some that based on amino acid sequence could be attributed to either human glioma or mouse host origin. Of specific interest was the observation that the IL-33-driven environment activated Stat3 signaling, as indicated by an increase in phosphorylated (p)-STAT3 (pY705), a state that has been associated with glioma progression^86,87^. To validate the cellular origin of the p-STAT3, we used multiplex IHC with human (h-nucleolin) and mouse (Iba1) markers. We found a substantial increase in p-STAT3 in both the glioma cells expressing IL-33 and the Iba1^+^ cells, a result consistent with a bidirectional signaling between the glioma cells and the Iba1^+^ TAM (Fig. 8b, f). Although the specific driver(s) of this signaling have yet to be determined, the expression of IL-6 and LIF by the IL-33^+^ glioma cells (Fig. 4c) represent known STAT3-regulated cytokines^88^. In contrast, in ΔNLS tumors, p-STAT3 was confined to the Iba1^+^ cells (Fig. 8b, f). Consistent with this, direct assessment of patient tumors by immunohistochemistry of the TMA found that high levels of p-STAT3 significantly correlated with expression of IL-33 (Fig. 8c) and consistent with these observations, high levels of p-STAT3 were detected in IL-33-expressing BT147, BT53 and the PDGFB-driven glioma models in vivo (Fig. 8d–f).

### IL-33-expressing glioma recruit peripheral immune cells

**Fig. 9:**
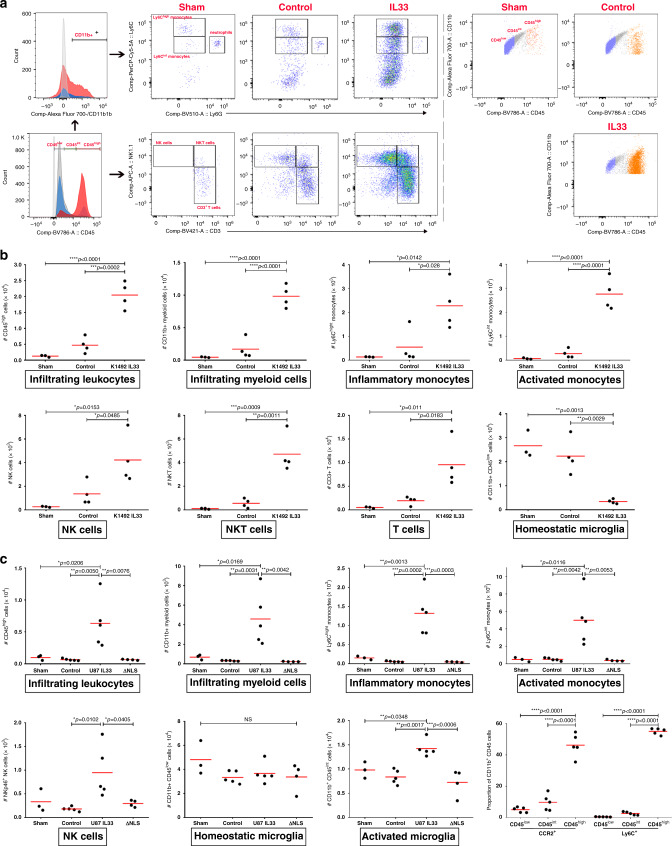
IL-33^+^ glioma associate with recruitment of peripheral immune cells. **a** Pseudocolor plots and histograms shown are from representative sham, K1492-control, and K1492-IL-33 tumor-bearing mice. For gating strategy see also Supplementary Fig. 7. Histogram: red represents K1492-IL-33, blue represents K1492-control, dark gray represents sham, and light gray represents isotype control for respective fluorochrome shown. Lower left histogram shows the distribution of live CD45^low^, CD45^int^, and CD45^high^ cells within the leukocyte gate. Upper left histogram shows the distribution of cells within the CD45^high^ population that are CD11b^+^ (i.e., myeloid cells). Upper panels show distribution of cells in sham, K1492-control, and K1492-IL-33 glioma within the CD45^high^CD11b^+^ gate that are Ly6G^+^ (neutrophils), Ly6G^−^Ly6C^high^ (inflammatory monocytes), and Ly6G^−^Ly6C^int^ activated monocytes. Lower panels show distribution of cells in sham, K1492-control, and K1492-IL-33 glioma, within the CD45^high^ gate that are CD3^+^NK1.1^−^ (CD3 T cells), CD3^+^ NK1.1^+^ (NKT cells), and CD3^−^NK1.1^+^ (NK cells). Plots on the far right show the distribution of CD45^+^CD11b^+^ cells that are CD45^low^ (microglia; blue), CD45^int^ (activated microglia; gray), and CD45^high^ (infiltrated myeloid cells; orange) in sham, K1492-control, and K1492-IL-33 glioma. Scatter plots show the number of cells as indicated on left axis, as isolated and quantified per tumor-bearing hemisphere in **b** sham, K1492-control, and K1492-IL-33 glioma, and **c** sham, U87-control, U87-IL-33, and U87-ΔNLS. In **c**, bottom far right scatter plot shows the proportion of cells that are CCR2^+^ or Ly6C^+^ amongst the CD11b^+^ cells that are CD45^low^, CD45^int^, or CD45^high^. Each point represents data from a single mouse. The experiment was repeated three times with comparable results. Red line indicates the mean. NS not significant; *p < 0.05, **p < 0.01, ***p < 0.001 by one-way ANOVA with Tukey’s post hoc test. See also Supplementary Fig. 7. Source data are available as a Source data file.

The above assessment of TIF from mice bearing IL-33^+^ vs. IL-33^−^ 1492 glioma showed that chemokines, such as CCL2, CCL5, KC, and CXCL10 are enriched within the IL-33^+^ tumor microenvironment (Fig. 7a). These chemokines are potent for driving recruitment of leukocytes, such as monocytes, NK cells, and T cells. Furthermore, our immunohistochemical data demonstrates that IL-33^+^ syngeneic glioma, and IL-33^+^ xenografts associate with an increase in Iba1^+^ cells in the tumor-bearing hemisphere (Fig. 3 and Supplementary Fig. 3). It is well known that TAMs within the glioma microenvironment comprise a mixture of microglia and infiltrating monocyte^89,90^. Since Iba1 is expressed by both microglia and infiltrating monocytes, it does not distinguish between the two cell types, and thus we assessed the ontogeny of the TAMs by characterizing leukocytes from the tumor-bearing hemisphere of IL-33^+^ and IL-33^−^ xenografts, using flow cytometry outlined in Fig. 9a. Here, we examined if the enrichment of chemokines and the increase in Iba1^+^ cells, observed in IL-33^+^ syngeneic and xenograft mouse brains were associated with recruitment of circulating immune cells into the IL-33^+^ tumor environment. First, leukocytes were isolated from the tumor-bearing hemisphere of IL-33^+^ and IL-33^−^ 1492 syngeneic mice, and characterized using flow cytometry. The gating strategy used to characterize the isolated cerebral leukocytes is shown in Supplementary Fig. 7a. The total number of isolated CD45^+^ leukocytes was two-fold higher in IL-33^+^ vs. IL-33^−^ 1492 tumor-bearing mice. Using differential levels of CD45 to distinguish between resident and infiltrating immune cells, we found a striking increase in the presence of CD45^high^ cells within the tumor-bearing hemisphere of IL-33^+^ vs. IL-33^−^ syngeneic mice. To determine which cells were infiltrating the IL-33^+^ brains, we used the following panel of markers: CD3 to identify T cells, Nk1.1 to detect NK cells, and a combination of CD11b, Ly6G, and Ly6C to distinguish myeloid cells (i.e., neutrophils and monocytes). There was a significant increase in the number of CD11b^+^ myeloid cells, T cells, NKT, and NK cells present in the tumor-bearing hemisphere of IL-33^+^ vs. IL-33^−^ 1492 syngeneic mice (Fig. 9b). When we further assessed if the increase in CD11b^+^ myeloid cells was the result of an increase in numbers of neutrophils and monocytes, we found no significant difference in the number of neutrophils present in IL-33^+^ glioma, however, there was a pronounced approximately four-fold increase in the number of inflammatory Ly6C^high^ monocytes (Fig. 9). Furthermore, using differential expression of CD45 on CD11b^+^ cells to distinguish between resident microglia (i.e., CD45^low^), activated microglia (i.e., CD45^intermediate^), and cerebral infiltrating leukocytes (i.e., CD45^high^) a significant increase in activated microglia was seen in the IL-33^+^ vs. IL-33^−^ 1492 syngeneic glioma, and this increase was associated with a decrease in the number of resting (homeostatic) microglia. Concomitant with this and as mentioned, there was also an increase in FOXP3^+^ T cells (Fig. 6).

Next, using the xenograft model where we can compare IL-33 expression with the expression of IL-33 crippled for its ability to enter the nucleus (ΔNLS), we observed a similar IL-33-mediated activation of microglia along with the recruitment of monocytes and NK cells, where increases of ~2.1- and ~4.6-fold were observed in IL-33-expressing tumors compared to tumors lacking nuclear localization (Fig. 9c). In addition, when the mean fluorescence intensity of Ly6C and CCR2 expression, markers expressed on monocytes but not microglia were also assessed, we found significantly higher expression on the CD11b^+^CD45^high^ cells vs. CD11b^+^CD45^low^ cells (Fig. 9c). Consistent with this, NanoString nCounter analysis using the nCounter Mouse v2 Inflammation Panel revealed a significant increase in CCR2 expression in the IL-33^+^ xenografts (Supplementary Fig. 7b). Furthermore, when we examined the presence of CCR2-expressing monocytes in PDGFA- or PDGFB-driven tumors in CX3CR1^GFP^/CCR2^RFP^ reporter mice where microglia (CX3CR1^high,^ CCR2^−^; green) are distinguished from monocyte-derived macrophage (CX3CR1^+^CCR2^+^; green and red), we again found an increase in monocytes in the IL-33^+^ PDGFB-driven tumors (Supplementary Fig. 7c).

**Fig. 10:**
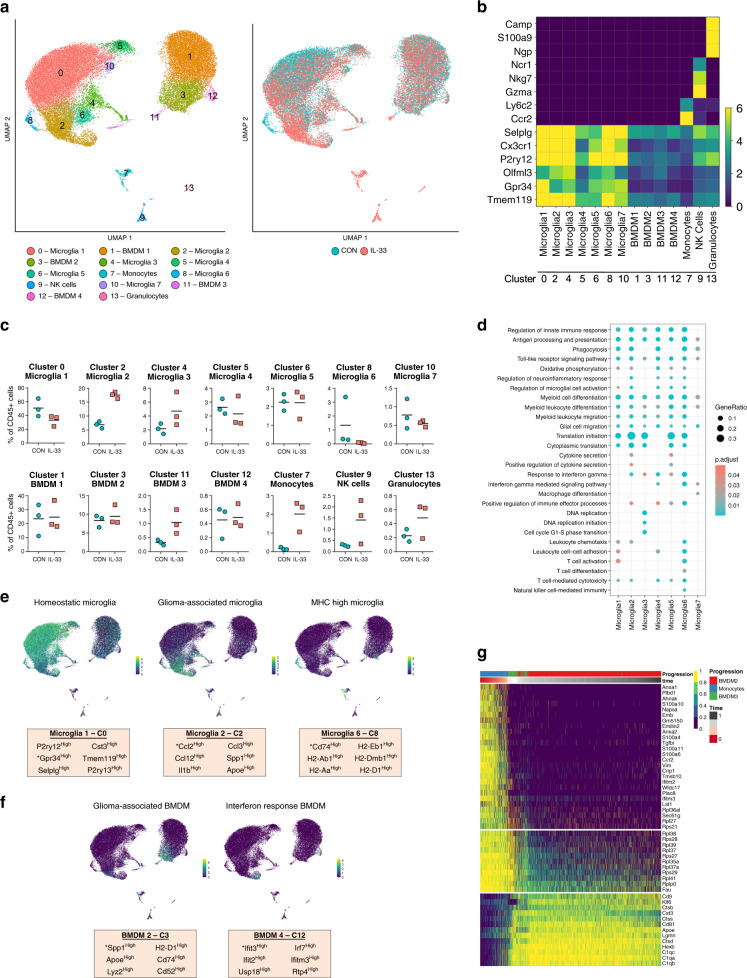
scRNAseq identities 14 distinct immune cell populations in glioma. **a** UMAP plot of 38,471 single cells from control (CON; *N* = 3) and IL-33^+^ (*N* = 3) intracranial U87 xenografts, displays the separation of cells into 14 clusters (left). UMAP plot on right shows overlay of cells from IL-33^−^ (blue) and IL-33^+^ (red) xenografts as distributed in the 14 clusters. **b** Heatmap shows the mean expression of lineage markers in each of the 14 clusters used for cluster annotation (see also Supplementary Table 1). **c** Dot plots show the percentage of cells out of the total CD45^+^ cells from IL-33^−^ and IL-33^+^ xenografts present in each cluster. Each data point, shown in blue (IL-33^−^) or red (IL-33^+^), represents an independent single-cell experiment performed on individual animals. **d** Dot plot shows selected enriched Gene Ontology terms in the seven microglial clusters determined using the R package clusterProfiler. Top differentially expressed genes (up to 200) were considered using the genesorteR package with default parameters. Size of each dot corresponds to the ratio of genes expressed in the cluster to the total number of genes related to the ontology term and color corresponds to the Benjamini–Hochberg-adjusted *p*-value for multiple comparisons for the specific term in each cluster. **e**, **f** UMAP plots show the expression of top differentially expressed genes in microglial and BMDM clusters. Asterisks indicate gene depicted in UMAP plot. **g** Heatmap of marker genes (ordered into modules) determined by SCORPIUS trajectory analysis of monocytes, differentiating BMDM (BMDM3), and glioma-associated BMDM (BMDM2). See also Supplementary Figs. 8 and 9. Source data are available as a Source data file.

Since the above results are consistent with IL-33-expressing GBM recruiting peripheral immune cells, we extended the characterization of CD45^+^ immune cells using single-cell RNA sequencing (scRNAseq). To maintain focus on the innate immune cell repertoire, we chose to perform these studies in IL-33^+^ and IL-33^−^ xenografts, a model that allows assessment of the differences in activation states within the tumor immune repertoire, as well as the recruited innate immune cells, such as monocytes and NK cells are reported to be enriched within a glioma tumor environment^91–95^. Cerebral leukocytes were isolated as described above from IL-33^+^ and IL-33^−^ tumor-bearing hemispheres, sorted for CD45, and RNA sequenced using the 10× Chromium platform. Following quality control and preprocessing, a total of 38,471 cells from three control (20,308 cells) and three IL-33-expressing (18,163 cells) xenografts were recovered for analysis (Supplementary Data 1). Unsupervised clustering of all cells delineated 14 distinct clusters shown by Uniform Manifold Approximation and Projection (UMAP) in Fig. 10a. To ascertain the identity of these clusters, we utilized a combination of lineage-defining genes and published immune cell signatures^92,93,96–103^ (Fig. 10b and Supplementary Table 1). Three clusters (i.e., clusters 7, 9, and 13) represent cells not normally present within the brain parenchyma^93^, and hence signify cells that infiltrate the glioma-bearing brain tissue. Of these, clusters 7 (monocytes) and 9 (NK cells) were predominantly present in the IL-33-expressing xenografts (Fig. 10c), observations consistent with the flow cytometry studies shown in Fig. 9. A summary of the top activated or inhibited canonical pathways in the monocytes and NK cells in the IL-33^+^ xenografts are shown in Supplementary Fig. 7d. The existence of these peripheral myeloid cells within the IL-33-expressing tumors supports the association of glioma-derived IL-33 with mediators that promote recruitment of cells from the circulation into the brain.

Recent studies indicate that microglia display considerable heterogeneity and diverse functional phenotypes attributed to age, gender, spatial distribution, and neurological disease states^104^. Consistent with this, we delineated seven discernable and identifiable microglia populations (microglia 1–7 (Fig. 10b)). We examined the diversity of these populations using GO enrichment (Fig. 10d) together with the alignment of the top differentially expressed genes within each cluster, when compared specifically among the seven microglial populations and gene signatures defined at single-cell resolution^105–107^ (Supplementary Fig. 8a, b and Supplementary Table 2). Differential gene expression comparison between the seven microglial populations (Supplementary Fig. 8a) showed that microglia 1 (cluster 0) comprises the largest population of microglia with high expression of homeostatic microglial genes^98,104,107^, including *P2ry12*, *Gpr34*, *Selplg*, *Cst3*, *Tmem119*, and *P2ry13* (Fig. 10e and Supplementary Fig. 8b, c), a cluster that is composed of considerably more cells from the IL-33^−^ xenografts (Fig. 10c). While demonstrating some differences, microglia 7 (cluster 10) also displayed a homeostatic signature (e.g., *P2ry12*, *Gpr34*, *Cx3cr1*, *Selplg*, and *P2ry13*); however, the presence of this population was independent of IL-33 status (Fig. 10c). In comparison, microglia 3 (cluster 4) contained a population of cycling and proliferating cells characterized by high expression of genes involved in cell cycle regulation and DNA replication^106,108^, with corresponding GO terms enriched in these biological processes (Fig. 10d and Supplementary Fig. 8a, b, d). In addition and consistent with emerging descriptions of activated myeloid cell signatures in the glioma microenvironment, we also observed a population of glioma-associated microglia^107,109,110^ (microglia 2 (cluster 2)) that express *Ccl2*, *Ccl12*, *Ifitm3*, and *Il1β* (Fig. 10e and Supplementary Fig. 8a, b, e) and when compared to the other microglial populations show enrichment for the pro-inflammatory cytokine osteopontin, *Spp1*, and the lipid metabolism gene, *Apoe* (Fig. 10e or Supplementary Fig. 8a, b, e). Of interest, we found that these glioma-associated microglia were nearly threefold higher in the IL-33-expressing xenografts than the control (Fig. 10c), an observation supporting IL-33 as a driver of an activated glioma microenvironment. These microglia also displayed considerable upregulation of the monocyte chemoattractant genes *Ccl2*, *Ccl3*, and *Ccl12*, linking them to the recruitment of infiltrative immune cells (Supplementary Fig. 8a, b). To delineate the specific effects of IL-33 in this population of microglia, we compared gene expression between IL-33^+^ and IL-33^−^ cells within microglia 2 (Supplementary Fig. 8f), and found suppression of homeostatic genes (*P2ry12*, *Gpr34*, *Tmem119*, and *Selplg*) together with an enhancement of *Spp1*, *Ifitm3*, and *Ccl2* in the IL-33^+^ tumors. A comparable glioma-associated signature was also observed in microglia 5 (cluster 6 (e.g., *Ccl3*, *Apoe*, and *Lpl*) Supplementary Fig. 8a, b), and within these two microglial clusters, we observed a similar enrichment of GO terms related to cytokine secretion^107^ and protein translation^110^ (Fig. 10d). In addition, we found that, microglia 6 (cluster 8) was uniquely enriched in the IL-33^−^ tumors (Fig. 10c) and expressed genes involved in antigen presentation^106,108^ (e.g., *Cd74*, *H2-Aa*, and *H2-Ab1* (Supplementary Fig. 8a, b, g)). GO analysis also showed enrichment of terms related to T cell function (Fig. 10d) and thus microglia 6 was identified as a MHC high microglial population^108^ (Fig. 10e and Supplementary Fig. 8g). The absence of this population of microglia in the IL-33^+^ tumors further suggests that IL-33 may, in part, drive a pro-tumorigenic environment via immune suppression. Lastly, microglia 4 (cluster 5) was not reminiscent of any published signature (Supplementary Fig. 8a, b) and remains to be defined, but is not unique to the IL-33^+^ environment.

The remaining immune cells in the glioma microenvironment were identified as peripheral myeloid cells, BMDM, which clustered separately from the microglia and also displayed similar heterogeneity (BMDM 1–4 (Fig. 10b and Supplementary Fig. 9a–c)). While it is clear that different populations of BMDM exist, they do not appear to conform with classical signatures derived by cell type algorithms^93,97^ (e.g., BMDM1 express core microglial genes, including *Selplg*, *P2yry12*, and *Tmem119* albeit at low levels (Fig. 10b)), an observation that may reflect the unique environment of the brain in which these cells reside^93^. Nevertheless, within these BMDM populations the following were noted: (1) BMDM 2 (cluster 3), similar to microglia 2, exhibited high expression of *Spp1* and *Apoe* genes^107,109^, and were therefore characterized as glioma-associated BMDM (Fig. 10f and Supplementary Fig. 9a, b, d); (2) BMDM 4 (cluster 12), shows substantial expression of genes involved in interferon signaling, including *Ifit2*, *Ifit3*, and *Irf7* (refs. ^105,106,108^; Fig. 10f and Supplementary Fig. 9a, b, e), and thus denotes interferon responsive BMDMs, and (3) BMDM 3 (cluster 11), which was selectively enriched in the IL-33^+^ xenografts, express a gene signature with features of both infiltrating monocytes and glioma-associated BMDM^111^ (Supplementary Data 1). Moreover, BMDM three expressed genes involved in protein translation signifying a population of actively differentiating cells^111^. Since we were particularly interested in the capacity of IL-33 to recruit monocytes, which differentiate into macrophages within the brain, we ordered monocytes, differentiating BMDM (BMDM 3), and glioma-associated BMDM (BMDM 2) within the IL-33^+^ xenograft in a pseudo-time trajectory to understand this transition (Fig. 10g). As predicted, we observed a transition from IL-33 enriched inflammatory monocytes^100^ (marked by expression of *Ccr2*, *Ifitm2*, and *Ifitm3*) to differentiating BMDM and then to glioma-associated BMDM^107,110,111^ (marked by expression of *Apoe*, *Hexb*, and *Cd81*) consistent with a pro-tumorigenic environment. In addition, a gradual down regulation of ribosomal genes (e.g., *Rpl* and *Rps*) is seen within the trajectory, a feature akin to the myeloid lineage differentiation pattern observed by Athanasiadis and colleagues^112^ and consistent with a recent study that has mapped the diversity of non-parenchymal brain macrophage^111^.

## Discussion

Our study has highlighted a central role for glioma-derived IL-33 as an orchestrator of the brain tumor microenvironment that promotes glioma progression and builds from previous studies, providing more mechanistic insight into the functions of IL-33 in glioma biology^60–62,113^. First, we found that both nuclear and soluble IL-33 are present within a subset of GBMs, and that expression of IL-33 correlates with increased TAM infiltration and decreased the overall survival in GBM patients. Second, we showed that expression of IL-33 promotes tumor growth and TAM infiltration in intracranial xenografts, and that nuclear IL-33 is essential for its augmented effect on tumor growth. Third, we determined that nuclear IL-33 mediates the secretion of a number of inflammatory cytokines from glioma cells. Collectively, this IL-33-driven tumor environment promotes both the activation of resident microglia and the recruitment of bone-marrow-derived cells that collectively enhance glioma growth and progression. The data presented in this study highlight a major mechanism by which glioma cells regulate the brain tumor environment to facilitate tumor progression and most likely contribute to therapeutic resistance.

In the past few years, the capacity of IL-33 to regulate both the innate and adaptive immune systems, including its contribution to tissue homeostasis and environmental stress response, has received considerable attention^64^. This range of effects is thought to attribute to the inherent ability of IL-33 to act as both a nuclear repressor and an extracellular ligand for ST2 (refs. ^114,115^). Moreover, IL-33 has been observed in a number of cancers where an elevated level of IL-33 has been detected in the serum and tumor tissue of patients with gastric cancer^116^, hepatocellular carcinoma^117^, uterine leiomyoma^118^, lung cancer^119^, colorectal cancer^120,121^, breast cancer^122^, endometrial cancer^123^, and recently in glioma where IL-33 expression was shown to correlate with glioma grade, Panofsky performance score, and survival^61^.

In this study, we illustrate that glioma-derived IL-33 has a dramatic effect on glioma growth in vivo through its ability to alter the cellular state of the tumor microenvironment. Initial characterization of the in vivo glioma secretome identified IL-33 as a major constituent of the inflammatory phenotype. Mining both the TCGA database and our own patient glioma RNA-seq database^65,124^ provided additional evidence for the expression of IL-33 in ~50% of high-grade glioma, with little or no expression in low-grade glioma, astrocytoma, or other brain tumors (www.cBioportal.org). The presence of IL-33 was not limited to human glioma as IL-33 was observed in specific murine models of GBM, including a syngeneic Nfl^*−/+*^/Trp53^*−/+*^ model^79,125,126^ and PDGFB-driven tumors that have a more mesenchymal stroma-like phenotype^80^. In both the human and mouse IL-33^+^ glioma models there was a correlation with high macrophage/microglial infiltration with tumor-promoting capacity. We found that the mere expression of IL-33 in non-expressing glioma cells is sufficient to promote rapid tumor growth and dramatically reduces the overall survival. Moreover, we observed that IL-33 had both direct and indirect effects on regulating the recruitment and activation of bone-marrow-derived cells, T cells, NK cells, and resident microglia. We also observed that glioma-derived IL-33 results in an increase in p-STAT3 in both glioma cells and Iba1^+^ stromal cells, with subsequent loss of p-STAT3 within glioma cells deficient for nuclear IL-33 (ΔNLS). This observation is consistent with bidirectional signaling between the glioma cells and the Iba1^+^ microglia/macrophage, a communication that may be mediated by the presence of IL-6 and LIF within the environment.

As mentioned, IL-33 has been shown to participate in tissue homeostasis by modulating cytokines; however, the exact mechanisms underlying this regulation remain largely unknown. Moreover, while it is well appreciated that IL-33 has distinct nuclear localization in a wide array of cell types, the exact biological function of nuclear IL-33 remains unclear and may be questionable^127^. Initial studies showing nuclear localization of IL-33 were performed in human endothelial cells^115^, where IL-33 was shown to associate with chromatin^49,50^ and act as a transcription factor by binding the transcription repressor histone methyltransferase (SUV39H1)^49^. In addition, IL-33 has been shown to regulate NF-kβ signaling by binding to the promoter of subunit p65 (ref. ^128^), or by physically interacting and sequestering NF-kβ (ref. ^129^). IL-33 contains an N-terminal chromatin-binding motif (amino acids 40–58)^50^ and a predicted bipartite nuclear localization signal (amino acids 61–78)^115^, and using a mutant that cripples both of these domains, we revealed the functional requirement for nuclear IL-33 in gliomagenesis. The nuclear capability of IL-33 drives the secretion of a number of cytokines, an observation not seen when nuclear localization was crippled. In fact, over time in the absence of nuclear IL-33 there is very little evidence of any tumor burden. This is in striking contrast to the presence of both secreted and nuclear IL-33 where expression drives gliomagenesis, an observation independent of whether the data is evaluated from human gene expression analysis, BTIC/glioma cell xenografts, or the PDGFΒ-driven murine glioma mouse model. This rapid growth is fueled by the recruitment of macrophage with a pro-tumorigenic phenotype with human IL-33^+^ GBM surgical specimens showing a positive correlation with M2 macrophage and T-regulatory cells, and a negative correlation with M1 macrophage and cytotoxic T cells, an environment that associates with poorer overall survival. Thus the vast changes in gene expression support a more global role for IL-33 in mediating changes in chromatin structure and reorganization^49,50^, an observation that warrants further investigation. The concept of the IL-33 paradox^130^ as to whether it can enhance or inhibit tumorigenesis may be explained at least in part by the relative functions of nuclear vs. secreted, a concept that should be pursued.

There remains considerable debate as to the mechanism(s) by which IL-33 is secreted from cells partly based on the lack of a conventional leader peptide to target the protein through the Golgi and endoplasmic reticulum secretory pathway^127^. Instead, the identification of IL-33 as an alarmin released from damaged cells has led to the growing consensus that the release of IL-33 occurs from areas of necrosis or tissue damage^131–133^. This is supported by the observation that full-length IL-33 is rendered biologically active via cleavage by inflammatory proteases to a mature, bioactive form (hIL-33_112-270_) that has up to 30-fold higher biological activity than full-length IL-33 (refs. ^76,77^). In addition, when cells are treated with a nuclear export inhibitor like leptomycin B there is a reduction in secretion, suggesting involvement of the nuclear pool of IL-33 (ref. ^134^). Although very little IL-33 is released from glioma cells in vitro, substantial quantities of IL-33 detected in the interstitial secretome in vivo. While human glioma can have necrotic regions where IL-33 could be released, many in vivo models utilized in this study show very little evidence for necrosis. Instead, we postulate that other mechanisms are employed for the secretion of IL-33 from living cells, posit for which there is supporting data^135,136^.

Taken together, these data support that, similar to IL-1α and HMGB1 (ref. ^71^), IL-33 has a dual function in glioma by acting as both a pro-inflammatory cytokine and an intracellular nuclear factor, functions required to drive glioma progression. Of note, glioma cells appear to lack the ST2 receptor and rIL-33 had no effect on in vitro glioma growth. This result contrasts colorectal cancer cells that express ST2 and respond to exogenous IL-33 (ref. ^137^). Instead, our data is consistent with glioma-derived IL-33 regulating the tumor environment by inducing the secretion of both glioma-derived and stromal-derived cytokines that activate resident microglia, and also recruit and activate pro-tumorigenic M2-like macrophages, similar to GBM that secrete periostin^138^. Assessment of the TCGA and our RNA-seq data, however, show no direct association between IL-33 and periostin, thus suggesting an alternative independent mechanism. Rather, our observations are consistent with a recent study showing the majority of TAMs recruited to PDGFB-driven GBM are bone marrow derived^89^; thus, providing at least one means by which this environment induces cellular recruitment.

We propose the following model that could have important therapeutic implications. Secretion of IL-33 from glioma cells acts to recruit monocytic cells from the circulation that contribute to the cellular composition within the brain tumor environment. The cells initially recruited to the tumor have a phenotype that is consistent with an M1-like anti-tumorigenic state, however, when nuclear IL-33 is present within the glioma cell, an environment is generated that favors the reprogramming of the TAMs and potentially resident microglia to a pro-tumorigenic M2-like phenotype that in turn fuels rapid glioma growth. Here, we found that the BMDM present in the IL-33^+^ glioma together with resident microglia, have a number of distinct transcriptional networks (Fig. 7) expressing both pro-inflammatory (M1) and alternatively activated (M2) genes simultaneously. This is consistent with studies showing that IL-33 acts directly on the resident microglia and contributes to their altered phenotype^58,139^, and that human glioma specimens contain macrophage with diverse states^140^. Independent it is clear by the data provided herein that the IL-33 environment educates TAMs to drive glioma progression and that the nuclear function of IL-33 is required. This idea is further supported by studies by Joyce and colleagues that suggest CSF-1 blockade prevents circulating recruitment of leukocytes, and thus may have therapeutic benefit^141^. While an appealing strategy, systemic CSF-1 blockade presents hurdles with respect to effects on the host immune response. We would instead postulate that modulating the phenotype of the macrophage within the glioma environment per se might warrant a more precision-based approach, a strategy consistent with our study using amphotericin B to reprogram the brain tumor environment^39^. While there are therapeutic strategies emerging to sequester secreted IL-33 using soluble ST2 receptors^142^, our data provides the provocative notion that targeting nuclear accumulation of IL-33 exclusively may have therapeutic benefit by maintaining a tumor environment with TAMs of a tumor-suppressive phenotype. In addition, as suggested by Quail and Joyce, the tumor microenvironment can be further modified during the course of treatment, and thus should be taken into consideration when examining responders and nonresponders to a specific therapeutic regimen^16^. Thus, in the era of immunotherapy and oncolytic viral therapy one may speculate whether an IL-33 orchestrated tumor environment may be beneficial, or inhibitory for these therapies and stratification of patients based on IL-33 or TAM status may need to be considered for the design of informative clinical trials. Furthermore, we would postulate that the cytokine storm that is orchestrated by IL-33 could bypass or overcome the therapeutic efficacy of small-molecule inhibitors, such as the tyrosine kinase inhibitors, which would contribute to their failed experience in the clinic.

## Methods

### Cell lines

The human U87, U251 glioma cell line, and U937 cell line were obtained from the American Type Culture Collection, and the murine K1491 and K1492 glioma cell lines were derived from C57Bl/6J *Trp53*^+/−^/*Nf1*^+/−^ mice^79,125^. U87, U251, K1491, and K1492 were maintained in complete medium (Dulbecco’s Modified Eagle’s Medium (DMEM)) supplemented with 10% heat-inactivated fetal bovine serum (FBS), 1% penicillin/streptomycin, 2 mM L-glutamine, and 1 mM sodium pyruvate at 37 °C in a humidified 5% CO_2_ incubator. Cells were passaged by harvesting with Puck’s EDTA when they reached 80–90% confluence. Stable transfectants of U87 or U251 cells were maintained in the same medium without antibiotics and with the addition of 400 μg/ml G418. Stable transfectants of K1492 cells were maintained in the same media without antibiotics and with the addition of 1 μg/ml puromycin. U937 cells were cultured in RPMI 1640 containing 10% FBS and 10 μM β-mercaptoethanol. The suspended U937 cells were treated with phorbol-12-myristate 13-acetate (PMA; 50 ng/ml). After 2-day treatment with PMA, U937 cells adherence and acquire characteristics of macrophage^143^ at which time the cells were used. BTICs were established within the Brain Tumor Stem Cell Core at the University of Calgary^12,47^. BT25 and 147 cells were maintained in serum-free culture medium (SFM) containing DMEM/Ham’s F12 (1:1) with 5 mM HEPES buffer, 0.6% glucose, 3 mM sodium bicarbonate, 2 mM glutamine, 25 μg/ml insulin, 100 μg/ml transferrin, 20 nM progesterone, 10 μM putrescine, and 30 nM selenite supplemented with epidermal growth factor (EGF; 20 ng/ml), fibroblast growth factor 2 (FGF2) (20 ng/ml), and heparin sulfate (2 μg/ml) at 37 °C in a humidified 5% CO_2_ incubator. BT53 and 73 cells were maintained in identical SFM without EGF and FGF2. BTIC spheres were grown until they reached a certain size (~100–200 μm) for passaging. Spheres were then dissociated into a single-cell suspension using Accumax resuspended in the adequate media. Mouse BMDM were isolated from the bone marrow of the femoral and tibial bones of C57BL/6 mice^144^. BMDM were differentiated in complete medium supplemented with 1% nonessential amino acids (NEAA; Invitrogen) and 10% L929 cell CM (L929 medium) for 7 days. Fresh media was replaced on day 5 and day 7. After differentiation for 7 days, the differentiated BMDM were confirmed by flow cytometry using F4/80 antibody. All BMDM used in this study were over 95% F4/80 positive. Human adult or fetal microglia (>95% purity) were obtained from resected brain specimens from patients and fetal tissue respectively. Briefly, human adult microglia (HAM) were derived from adult human brain tissue obtained at surgical resection to treat intractable epilepsy^39,145^. In previous work, it was determined that the human microglia obtained for this study were similar to microglia derived from other neural pathologies or from the normal brain^146^. Human fetal microglia were isolated from brain tissue from 14–18-week human fetus^143^. Brain tissue was cut into 1 mm pieces and incubated in 0.25% trypsin, and 200 μg/ml DNase I in phosphate-buffered saline (PBS) for 15 min at 37 °C. Tissue suspension was washed through a 130 μm pore filter and centrifuged at 200 × *g* for 10 min. Pellet was washed twice in PBS and then plated in a T-75 flask coated with 10 μg/ml poly-ornithine in minimum essential medium supplemented with 10% FBS, 20 μg/ml gentamicin, 0.1% dextrose, 1× NEAA, 10 μM glutamine, and 1 mM sodium pyruvate. After 1 week, during which two medium changes occurred, floating cells were collected, and these were predominantly microglia (purity over 95%, as assessed using CD14 and Iba1 immunohistochemistry). Microglia were plated in a 96-well plate at a density of 1.5 × 10^4^ cells per well. All established cell lines used within this study were validated for identity by short tandem repeat analysis performed by Calgary Laboratory Services (CLS). DF 1 cells were maintained at 39 °C according to manufacturer instructions, used in early passages, and tested for mycoplasma.

### Human tissue samples

GBM TMA and primary GBM specimens were obtained from the Clark H. Smith Neurologic and Pediatric Tumor Bank at the University of Calgary. TMAs containing triplicate 0.6 mm cores from surgically resected, formalin fixed, paraffin-embedded glioma, and TMAs cores represented 64 GBM specimens. Human brain tissue removed during surgical resection to treat drug-intractable epilepsy was used to obtain HAM. Human fetal microglia were obtained from human fetus following therapeutic abortion. Informed consent for use of brain material for research was obtained from the patients. All human tissues were used with approval and in concordance with the policies of CLS and the Conjoint Health Research Ethics Board at the University of Calgary. Study protocol HREBA.CC-16-0762 was reviewed and approved by provincial ethics board HREBA-CC (and was formerly approved as protocol #2975 by the CHREB board at the University of Calgary). Associated specific ethics approval for culture and molecular characterization of the de-identified samples are approved under protocols HREBA.CC-16-0763 and HREBA.CC-16-0154.

### Animals

Six- to 8-week-old female SCID or C57BL/6J mice were housed in groups of three to five, and maintained on a 12-h light/dark schedule with a temperature of 22 °C ± 1 °C and a relative humidity of 50 ± 5%. Food and water were available ad libitum. All procedures were reviewed and approved by the University of Calgary Animal Care Committee (Animal Protocol #AC18-0073). PDGFA-, PDGFB-, and shNF-1+Cre-induced immunocompetent mouse glioma were generated in *N/tv-a;Cdkn2a*^*−/−*^*;Ptenfl/fl* mice using the RCAS/tv-a system^147,148^. These animal experiments were done in accordance with protocols approved by the Institutional Animal Care and Use Committees of FHCRC, and followed NIH guidelines for animal welfare (Animal Protocol #50842). *Cx3cr1*^*GFP/WT*^; *Ccr2*^*RFP/W*T^; *Nestin-tv-a*; *Pten*^*fl/fl*^ mice were generated by crossing *Cx3Cr1*^*GFP/GFP*^; *Ccr2*^*RFP/RFP*^ mice^89^ into *Nestin-tva*; *Pten*^*fl/fl*^ mice^90^. *Cx3cr1*^*GFP/WT*^; *Ccr2*^*RFP/W*T^; *Nestin-tv-a*; *Pten*^*fl/fl*^ mice are in C57BL/6J background. *Cx3cr1*^*GFP/WT*^; *Ccr2*^*RFP/W*T^; *Nestin-tv-a*; *Pten*^*fl/fl*^ mice were housed in Emory University, Division of Animal Resources. Experimental procedures were approved by the Institutional Animal Care and Use Committee of Emory University (Animal Protocol #2013-1029).

### Orthotopic glioma generation

Patient-derived BTICs from newly diagnosed (BT73, BT63, BT67, BT127, BT140, and BT53) and recurrent (BT25, BT119, BT143, and BT147) tumors, U87 and U25 human glioma cells (5 × 10^4^ cells/mouse), and K1492 mouse glioma cells (1 × 10^5^ cells/mouse) were implanted into the brain of SCID or C57BL/6 mice by first anaesthetizing the mice, using an intraperitoneal injection of ketamine (85 mg/kg) plus xylazine (15 mg/kg; MTC Pharmaceuticals, Cambridge ON, Canada). Cells were injected into the right putamen of the brain at a depth of 3 mm through a scalp incision and a 0.5-mm burr hole made 1.5–2 mm right of the midline and 0.5–1 mm posterior to the coronal suture. The stereotactic injection was performed using a 5-µl syringe (Hamilton Co., www.hamiltoncompany.com) with a 30-gauge needle mounted on a Kopf stereotactic apparatus (Kopf Instruments, Tujanga, CA). Following the withdrawal of the needle, the incision was sutured and mice were monitored weekly^12,47,72,74^. Whole brain tissue sections from all animals in each group were examined by immunohistochemistry (IHC) at designated time points. Animals assessed for survival were monitored until they lost ≥20% of body weight or had trouble ambulating, feeding, or grooming, or until the experiment was terminated.

### Generation *PDGFA-*, *PDGFB*-, and *shNF-1*-induced murine glioma

To generate PDGFA-, PDGFB-, and shNF-1-induced glioma, double-transgenic neonatal *N/tv-a*; *Cdkn2a*^−/−^; *Ptenfl/fl* mice (Xfm) were injected with DF1 chicken fibroblasts, producing RCAS retroviruses expressing either human PDGFB, human PDGFA, or shNF1 + Cre^80^. Mice were monitored carefully for symptoms of tumor development (hydrocephalus, lethargy).

### Generation of murine glioma in *Cx3cr1*^*GFP/WT*^; *Ccr2*^*RFP/WT*^; *Nestin-tv-a*; *Pten*^*fl/fl*^ mice

The same procedure described above was used to generate these mice with the exception that DF 1 cells were injected into *Cx3cr1*^*GFP/WT*^; *Ccr2*^*RFP/W*T^; *Nestin-tv-a*; *Pten*^*fl/fl*^ transgenic mouse. The same injection of DF1 chicken fibroblasts, producing RCAS retroviruses expressing a short hairpin against murine p53 (RCAS-shp53) and either human PDGFB or PDGFA was used to generate murine glioma into *Cx3cr1*^*GFP/WT*^; *Ccr2*^*RFP/W*T^; *Nestin-tv-a*; *Pten*^*fl/fl*^ mice. Mice were monitored carefully and were sacrificed if they display lethargy due to tumor burden. Postnatal 0–3-day-old mice were used in all experiments.

### Immunohistochemistry and immunofluorescence staining

IHC and IF staining of brain tissue sections were performed following formalin fixation, paraffin embedding, and sectioning of the brains using antibodies against IL-33 (1/100), h-nucleolin (1/500), Iba1 (1/500), Olig-2 (1/500), Arg1 (1/100), CD163 (1/200), CD4 (1/500), FOXP3 (1/200), pSTAT3 (1/50), RFP (1/500), and GFP (1/500) for 1 h at room temperature (RT). For IHC, a DAKO Envision and System-HRP Kit (Agilent Technologies) was used. Slides were counterstained with hematoxylin (Sigma-Aldrich), mounted, and imaged using a Zeiss inverted microscope (Axiovert 200 M) and camera (AxioCam MRc), or scanned using Aperio Scanscope® XT (Aperio Inc.) slide scanner at 20× or 40× resolution and images were acquired, using Imagescope v12.2.2.5015 software. To quantify macrophage levels in the brains, the total number of Iba1^+^ cells was quantified in five random fields of view on Iba1-stained brain tissue sections. To quantify the number of CD4 or FOXP3-positive cells in the brain tissue sections, the total number of CD4^+^ or FOXP3^+^ cells were counted in the whole brain tissue section using Qpath software. In GBM TMA and primary GBM specimens, IL-33, CD163, and Iba1 staining was scored on a scale from 0 to 3 by three independent observers. For IF, slides were incubated with fluorescence-conjugated secondary antibodies (Invitrogen) at RT for 1 h. Nuclei were counterstained with 5 µg/ml of DAPI. Multiplex tissue fluorescence staining was performed with the Opal staining system (Perkin Elmer). IF images were acquired using an In Cell Analyzer system (GE Healthcare) and stitched whole brain images were generated, using the In Cell developer toolbox 1.9.1 software. Antibodies are listed in the “Reagent and resource table” (Supplementary Table 6).

### Cytokine measurements

Cytokine levels were measured in TIF from intracranial xenografts and cell culture medium, using the Human 64-Plex Cytokine/Chemokine Panel and the Mouse 31-Plex Cytokine/Chemokine panel (Eve Technologies, Calgary, AB, Canada). TIF was prepared^149^ by removing the tumor-containing hemisphere that was weighted, dipped in ethanol, cut into small pieces (3 mm^3^), rinsed with PBS, and placed in a 10-ml conical plastic tube containing 1.0 ml of PBS per 250 mg of tissue. Samples were then incubated for 1 h at 37 °C in a humidified CO_2_ incubator. Thereafter, the samples were centrifuged at 150 × *g* for 3 min; supernatants were collected and further centrifuged at 2300 × *g* for 20 min in a refrigerated centrifuge (4 °C). The final supernatant was collected, aliquotted, stored at −80 °C, and then sent for multiplex analysis. In order to measure cytokine levels in cell culture medium from glioma and BTIC cells, cells were plated at density of 1 × 10^6^ cells/10 ml in complete media or BTIC media, respectively, and incubated for 48 h. The media was collected and sent for Luminex analysis. In order to measure cytokine levels in the cell culture media from human fetal microglia or BMDM following rIL-33 treatment, cells were plated at a density of 1 × 10^4^ cells/well in a 96-well plate. After 24 h, the media was replaced with rIL-33-containing media. Twenty four hours later the CM was collected and cytokine levels were measured by Luminex analysis.

### Western blot analysis and immunocytochemistry

Equivalent number of cells was plated in a six-well tissue culture plate. Forty eight hours later, 100 μl of 2× sodium dodecyl sulfate (SDS) sample buffer was added directly to the cells and the cell lysates were boiled for 15 min at 95 °C. To prepare the concentrated serum-free CM, the same number of cells were plated in a six-well plate and incubated for 12 h. The media was then replaced with serum-free media. After 48 h, 4 ml of CM was concentrated using 3 K Amicon to ~200 μl. For western blot analysis of the TIF or concentrated serum-free CM, 50 μl was mixed with the same volume of 2× SDS sample buffer and boiled for 15 min at 95 °C. Proteins were resolved by SDS–polyacrylamide gel electrophoresis, and western blot analysis was performed using the appropriate primary and secondary antibodies. For immunocytochemistry, cells were grown on coverslips, fixed in 4% paraformaldehyde, and permeabilized in 0.25% Triton X-100 prior to immunostaining. Primary mouse monoclonal anti-human IL-33 (1/100) or goat polyclonal anti-mouse IL-33 (1:100), and secondary Alexa Fluor 488–conjugated goat anti-mouse lgG were sequentially applied. Nuclei were counterstained with DAPI and coverslips mounted onto the glass slides. Images were acquired with a Zeiss LSM 880 confocal microscope equipped with Airyscan super-resolution imaging module, using a 63×/1.40 NA Plan-Apochromat Oil DIC M27 objective lens (Zeiss MicroImaging). Antibodies are listed in Supplementary Table 6.

### Cerebral immune cell isolation and characterization of CD11b^+^ GR-1^−^ cells

Cerebral immune cells were isolated from the tumor-bearing cerebral right hemispheres using mechanical dissociation by passaging the tissue through a 70-μm filter and then a 40-μm filter. Red blood cells in the tumor homogenate were lysed using hemolysis buffer (1 min, 4 °C) and isolated cells were incubated with Fc receptor blocking reagent (30 min, 22 °C) to block nonspecific antibody binding. To characterize myeloid cells, the following antibodies were used at a dilution of 1:100: PE anti-mouse CD11b and Alexa Fluor 647 anti-mouse GR-1. The Zombie Violet™ Fixable Viability Kit was used to exclude dead cells from analysis. Cells were then passed through an additional 40-μm filter to remove any large aggregates prior to running on the flow cytometer. Quantification of the populations of interest was normalized to the total number of events recorded by the flow cytometer such that the end number is the amount of population per 1 × 10^6^ events. To normalize the amounts of CD11b^+^ GR-1^−^ cells across all samples to events, first, the number of ungated events was recorded (e.g., IL-33 brain 1 = 2 × 10^6^ events), next a normalization factor (NF) was computed. NF = 1 × 10^6^/(number of ungated events). In this case, the NF = 0.5 and is used to normalize 2000 CD11b^+^ GR-1^−^ cells in IL-33 brain 1 to a number of events recorded, e.g., NF × 2000 = 1000 CD11b^+^ GR-1^−^ cells per 1 × 10^6^ events.

### Cerebral immune cell isolation and characterization by flow cytometry

Cells were isolated from the tumor-bearing right hemisphere^150^ as described above. Once a single-cell suspension of immune cells was obtained, cells were washed in PBS/1% FBS. To block Fc receptors, cells were incubated with CD16/CD32 antibody (22 °C, 10 min). For staining of surface markers, all antibody incubation was performed in 100 μL PBS/FBS (22 °C, 15 min). The following cocktail of antibodies was used to identify the different immune cell populations described in this study: CD45 (BV786), CD11b (AF700), CCR2 (APC), Ly6C (PerCP), Ly6G (BV510), CD3 (BV421), NK1.1 (APC), NKp46 (BV421), and CD19 (PeCy7). To determine proportion of live vs. dead cells, propidium iodide (5 μL) was added to the cell suspension prior to running on a flow cytometer. For sorting of CD45^+^ cells: cells from the tumor-bearing right hemisphere were isolated as above and labeled with CD45 (BV786) antibody. Cells were sorted with the BD FACSARIA III sorter. Samples were gated for CD45^+^ after exclusion of doublets. Sorted CD45^+^ cells were collected in tubes containing 1 ml PBS/1% FBS. Cell suspensions were kept on ice until ready to be loaded on Chromium Single Cell controller chip (10× Genomics).

### Mutagenesis and transfection

Human (NM_033439; hIL-33 pCMV6-XL5) IL-33 cDNA was from Origene. Human IL-33 cDNA cloned into pcDNA 3.1 vector to generate pcDNA3.1 hIL-33 plasmid. Human IL-33 ΔNLS deletion mutant was amplified by PCR using the pCMV6-XL5 human IL-33 cDNA as templates; sense primer contained a NotI site, and an ATG start codon, and an antisense primer contained a stop codon, and a NotI site. Primer sequences used to generate the IL-33 ΔNLS deletion mutant are F5′-GCGGCGGCCGCATGAAAAGGCCTTCACTGAAAACA-3′ (forward) and R5′-CGCGCGGCCGCAATCTAGAGTCGAGT-3′ (reverse). The PCR fragments, thus obtained, were cloned into plasmid pcDNA3.1 (Invitrogen). The sequence of all expression plasmids was validated prior to stable transfection. Glioma cells were stably transfected with pcDNA3.1 (Vector) or pcDNA3.1 human IL-33 using Lipofectamine 2000 (Invitrogen) following the manufacturer’s instructions. Stable transfectants were selected in media containing 400 μg/ml G418 (Invitrogen; concentration determined by toxicity curve for each cell line). Stable mouse glioma cell line (K1492) expressing mouse IL-33 (NM_001164724; mIL-33 pLenti-GIII-EF1α from Abm Inc) was generated using a lentiviral vector system^47^. Stable-infected K1492-IL-33 glioma cells were selected in media containing 1 μg/ml puromycin (Invitrogen; concentration determined by toxicity curve for each cell line). Transfections with RCAS PDGFA, PDGFB, or shNF1 + Cre1 (refs. ^80,89,90,151^) were performed using the Fugene 6 transfection kit (Roche, 11814443001) according to the manufacturer’s protocol.

### RNA sequencing

RNA was isolated from BTICs and their parent GBM tumors using the RNEasy extraction kit (Qiagen). High-throughput RNA sequencing was performed using Illumina HiSeq2000 and 2500, and analyzed RNA-Seq with Jaguar^152^ to include alignments to a database of exon junction sequences and subsequent repositioning onto the genomic reference. Normalized gene expression levels were evaluated by RPKM using GSC’s in-house WTSS pipeline coverage analysis (v0.4.6.9). The raw datasets are deposited in the EGA (European Genome-phenome Archive).

### Gene expression analysis

For microarray analysis of U87pcDNA, IL-33, and ΔNLS cells, RNA was extracted from 500,000 cells using mirVana miRNA Isolation Kit (Ambion) according to the manufacturer’s protocol. Total RNA was purified with RNeasy Plus Micro Kit (Qiagen) and RNA integrity number (RIN) was measured using the Agilent RNA 6000 NanoChip on 2100 Bioanalyzer (Agilent Technologies). Quantity was measured using NanoDrop 1000 (NanoDrop Technologies, Inc) and 100 ng of RNA with a RIN higher than nine was labeled with 3′ IVT Express Kit (Ambion) and hybridized to Affymetrix GeneChip Human PrimeView Arrays at 45 °C for 16 h. Arrays were stained using Affymetrix GeneChip Fluidics_450 following manufacturer’s protocol and scanned using the Affymetrix GeneChip Scanner 3000 7 G System. The raw datasets for array comparisons are deposited in the Gene Expression Omnibus (GEO) website (http://www.ncbi.nlm.nih.gov.geo/; accession number). Affymetrix GeneChip array data files were generated using GeneChip Command Console Software (AGCC) and statistical analysis was carried out using Partek Genomics Suite 6.0 (Partek Incorporated). Of the 20,000 genes represented on the array, the fold change was calculated as compared with control. Differential expression analysis was performed using DESeq2 software using fold change of greater than or equal to 2.0 and a FDR of 0.01. To categorize biologic functions related to gene expression altered by ectopic IL-33 expression, fold change files were uploaded into DAVID Bioinformatics Resources 6.7 (National Institute of Allergy and Infectious Diseases, NIH, Bethesda, MD). For gene expression data of PDGFA-, PDGFB-, and shNF-1-induced murine glioma, Vst Transformed and Quantile normalized data was downloaded from https://www.ncbi.nlm.nih.gov/geo/query/acc.cgi?acc=GSE45874 (refs. ^80,153^). The probe identification was mapped to gene symbols using R/Bioconductor package, illuminaMousev1p1.db^154^.

### NanoString nCounter gene expression analysis

Total RNA was isolated from intracranial xenografts derived from U87pcDNA, IL-33, and ΔNLS cells 1 week after tumor implantation. Tumor tissue was isolated and kept in RNAlater solution at −80 °C (Thermo Scientific). RNA was isolated using mirVana miRNA isolation kit (Ambion) according to the manufacturer’s protocol. Total RNA purification to remove genomic DNA was carried out with Qiagen kit of RNeasy® Plus Micro following the manufacturer’s protocols. RIN was assessed with RNA 6000 NanoChip on Agilent 2100 Bioanalyzer (Agilent Technologies). RNA samples with RIN 8 or higher were selected for gene expression analysis. RNA was quantified using the NanoDrop ND1000 spectrophotometer (Thermo Fisher Scientific). Gene expression analysis was conducted on the NanoString nCounter gene expression platform (NanoString Technologies). A code set of nCounter Inflammation Panel (Mouse v2) consisting of a 254-gene panel related to apoptosis, EGF, interleukin signaling, Ras, T cell receptor, and Toll-like receptor signaling was used. Per sample, 100 ng of total RNA in a final volume of 5 μl was mixed with a 3′ biotinylated capture probe and a 5′ reporter probe tagged with a fluorescent barcode from the nCounter Inflammation Panel (Mouse v2) gene expression code set. Probes and target transcripts were hybridized overnight at 65 °C for 16 h per the manufacturer’s protocol. Hybridized samples were purified on the NanoString nCounter Prep Station using the high-sensitivity protocol, in which excess capture and reporter probes were removed and transcript-specific ternary complexes were immobilized on a streptavidin-coated cartridge. The samples were scanned at maximum scan resolution on the nCounter Digital Analyzer. Data analysis was performed with nSolver software (NanoString Technologies). Samples that passed QC were further analyzed for fold change and normalized expression levels. The expression levels of each gene were normalized, to both positive control probes and housekeeping genes built-in each probe set, by the nSolver Analysis Software.

### Comparative IL-33 gene expression profiles of GBM and healthy brain and patient survival

The Oncomine database (www.oncomine.org) and Repository of Molecular Brain Neoplasia Data (REMBRANDT) of the National Cancer Institute (http://www.betastasis.com/) were used to analyze mRNA expression of IL-33 and its prognostic value in glioma. Heatmaps for the visualization of differentially expressed genes between IL-33 overexpressing and control glioma cells were prepared using pheatmap package in R. TCGA GBM expression and clinical data was downloaded from Broad Firehose GDAC (gdac.broadinstitute.org). The expression of M2 markers (MSR1, VEGFA TLR8, CD163, TGFB1, IL-10, and TLR1), M1 markers (CSF2, IL23A, TNF, and IFNG), cytotoxic T cell markers (CD8A, CD3E, CD8B, and IFNG), and T-regulatory cell markers (FOXP3, CD3E, and CD4) were summarized using ssGSEA^155^. Correlation matrices for visualizing associations between IL-33 expression and immune cells were constructed using the gcorrplot package in R. The association between IL-33 expression and survival was assessed using Univariate Kaplan–Meier analysis and logrank test. ssGSEA scores summarizing the expression of IL-33-associated genes (genes upregulated by IL-33 overexpression) were used for stratification of TCGA patients. The Wilcoxon rank-sum test was used to assess significance of correlations observed in box plots.

### Sample preparation for mass spectrometry

Phosphoproteomics sample acquisition and analysis was performed by SPARC BioCentre (Molecular Analysis), The Hospital for Sick Children, Toronto, Canada and the protocol is based on one developed by Rush and colleagues^156^. Rapidly frozen xenografts were lysed in urea lysis buffer (20 mM HEPES pH 8.0, 8 M urea, 1 mM sodium orthovanadate, 2.5 mM sodium pyrophosphate, and 1 mM β-glycerophosphate) via sonication. The lysates were then cleared by centrifuging at 60,000 × *g* for 15 min, protein concentration was determined in the resulting supernatant using the Bradford assay, and protein amount was normalized in all samples to 4.5 mg. A total of 4.5 mM DTT was added and lysates were placed at 55 °C for 20 min followed by the addition of 10 mM iodoacetamide followed by incubation at RT in the dark for 15 min. Lysates were digested with trypsin by diluting four times with HEPES buffer to a concentration of 2 mM urea and with the addition of trypsin-TPCK solution (Worthington Biochemical) to a final concentration of 10 μg/ml. Lysates were left at RT overnight. Salt was removed using Sep-Pak C18 column (Waters Corp), peptides were lyophilized for 2 days, and suspended in immunnoaffinity purification (IAP) buffer (50 mM MOPS pH 7.2, 10 mM sodium phosphate, and 50 mM NaCl). A total of 40 μl of pY antibody-tagged resin (Cell Signaling Technology) was then added and IAP was incubated at 4 °C for 2 h. Beads were washed three times with IAP buffer and peptides were eluted with 0.15% trifluoroacetic acid. Further purification was performed using ZipTips C18 (Millipore Corp).

### Mass spectrometry

Linear ion trap-Orbitrap Elite hybrid analyzer (LTQ-Orbitrap, ThermoFisher) with a nanospray source and the EASY-nLC split-free nano-LC system (ThermoFisher) was used to analyze samples. The lyophilized peptides were dissolved in formic acid (0.1%) and placed onto a 75 μm × 2 cm PepMap 100 Easy-Spray pre-column filled with 3 μm C18 beads. The pre-column was followed by an in-line 75 μm × 50 cm PepMax RSLC EASY-Spray column (600 Bar pressure) filled with 2 μm C18 beads (ThermoFisher San). Peptides were eluted at a rate of 250 nl/min for 60 min with a 0 to 35% acetonitrile gradient in formic acid (0.1%). The nano electrospray was used to introduce the peptides into an LTQ-Orbitrap hybrid mass spectrometer (ThermoFisher). The instrument method included a MS full scan (400–1500 *m*/*z*) using the Orbitrap mass analyzer, an automatic gain control target of 1e6 with a maximum ion injection of 120 ms, a microscan, and a resolution of 240,000. Ten data-dependent MS/MS scans were performed in the linear ion trap with the ten most intense ions having a 35% normalized collision energy. MS and MS/MS scans were obtained in parallel. When the MS/MS mode was being used, the automatic gain control targets were 1e5 with a maximum ion injection time of 50 ms and a minimum ion intensity of 5000. The normalized collision energy was set at 35 and the dynamic exclusion was used with a maximum exclusion list of 500 with a repeat count with a repeat duration of 30 s and exclusion duration of 8 s. The raw MS files were processed using the standard workflow^157^ with the MaxQuant software (version 1.5.3.30). MS/MS spectra were searched against the Human Uniprot database with the Andromeda search engine built into MaxQuant^158^ using a false discovery rate of 0.01. Bioinformatics analysis was completed using the Perseus tools within MaxQuant.

### Single-cell RNA sequencing

CD45^+^ cells from three pcDNA and three IL-33 xenografts were used for scRNAseq studies. Appropriate volume of cells, as determined from the Chromium Single Cell 3′ reagent kits User Guide user guide (v2 chemistry) for recovery of 5000 cells, was loaded on the Chromium Single Cell Controller chip. The Chromium Single Cell 3′ v2 library and gel bead kit was used to prepare scRNAseq libraries. Post cDNA amplification QC and quantification, as well as library Construction QC was done using an Agilent Bioanalyzer high sensitivity DNA chip for use with the Agilent 2100 Bioanalyzer. Prepared libraries were quantified using Kapa library quantification kit. For sequencing, the libraries were loaded at 1.3 pM on the Illumina NextSeq 500 system using the NextSeq 500/550 high output v2 sequencing reagent kit. Three libraries were sequenced with one sequencing reagent kit. A 26 base-pair read 1 was used to sequence the cell barcode and UMI, an 8 bp i7 index read was used to sequence the sample index and a 98 bp read 2 was used to sequence the transcript using paired-end sequencing.

### Analysis of scRNAseq data

A 10× genomics single-cell transcriptome sequencing data was processed using the Cell Ranger version 3.1 pipeline. Briefly, the FASTQ files were processed with the Cell Ranger Count pipeline, which uses STAR to align the reads to the mm10 mouse reference genome. The Cell Aggregate pipeline was run to generate an expression matrix with the combined libraries for that study. The three IL-33 and three pcDNA libraries were sequenced in three batches. The raw sequencing reads from the six samples were then combined and analyzed using the R package Seurat version 3.1.5 (refs. ^159,160^). All efforts were made to control batch effects, including using identical protocols, equipment, and the same personnel for all experiments. The extent of batch effects was evaluated by comparing clustering results based on (i) 10× aggregate output, or batch-corrected data by (ii) Seurat’s IntegrateData function, or (iii) Harmony batch correction^161^. Since <0.98% of cells changed cluster assignment upon batch correction, we opted to perform all downstream analyses on the 10× aggregate output to avoid unnecessary transformation of the data. The data were filtered for the following parameters: gene present in >3 cells, cells with >200 genes, and percent of mitochondrial genes <5%. Cells expressing >2500 genes were considered doublets and excluded (additional quality control metrics can be found in Supplementary Tables 3–5 and Supplementary Data 1). After filtering the combined object contained 38,471 cells and 16,520 genes. Data were log normalized and scaled using the respective Seurat functions. A PCA reduction was performed and only the first 15 PCA dimensions (determined using an elbow plot) were taken into account. An initial round of clustering revealed a population of choroid plexus epithelial cells defined by high expression of Ttr, Enpp2, Clu, and Igfbp2 (ref. ^162^). As this was inconsistent with the CD45 antibody sort and not an immune cell, it was excluded from further analysis. Differentially expressed genes for one cluster (vs. all cells in other clusters) were determined by a nonparametric Wilcoxon ranked-sum test. Cluster annotation was performed based on the expression of lineage-specific hallmark genes (Supplementary Table 1) and corroborated by inspection of the top differentially expressed genes in each cluster ranked by Bonferroni-adjusted *p*-value. The Python package, SCANPY version 1.4.7 was used to make the matrixplot visualizations^163^. For analysis of gene enrichment, the R package genesorteR was used to determine the top 200 marker genes of each cluster and clusterProfiler was used to identify enriched GO terms^164,165^. Trajectory inference was conducted using the R package SCORPIUS, using normalized data from Seurat^166^.

### Cell proliferation assay

To measure the growth rate of control, IL-33, and ΔNLS of U87 or U251, the cells were seeded at 1000 cells per well (100 µl of media) in a 96-well plate and incubated. Following for 3 days, 10 µl of Alamar Blue was added to each well of the 96-well plate and incubated for 4 h at 37 °C in 5% CO_2_. For assessment of BMDM cell proliferation, BMDM were plated in triplicate in 96-well, flat-bottom culture plates at a density of 2 × 10^4^ per well in 0.2 ml DMEM mixed with CM as indicated, and incubated for 72 h. Cell viability was determined using Alamar Blue. Cellular fluorescence in each well was measured at 570 nm using a microtitre plate reader. Data were converted into the relative cell viability (%) from the absorbance of control cells or media control (set as 100%).

### Transwell migration assay

Transwells (8 μm pore size, Millipore) were placed in 24-well plates and 1 × 10^5^ BMDM suspended in 200 μl serum-free DMEM were plated in the top of each chamber. A total of 500 μl of CM from glioma cells or CM containing IL-33 or CCL2 Abs (1 μg/ml, 1 h) was added to the lower compartment. After 4 h of incubation, chambers were washed and cells were removed from the upper side of the chamber with a cotton swab. Migrated cells were fixed and stained with 1% crystal violet in 95% ethanol. The average number of migrated cells from five representative fields was counted under a phase contrast microscope.

### Quantification and statistical analyses

Statistical tests were two-sided. *P* ≤ 0.05 was considered statistically significant. For comparison analyses limited to two groups, two-sided unpaired Student’s *t*-test was utilized (cytokine levels, macrophage quantifications by IHC, and phosphoproteomics). To study differences between three or more groups, one-way ANOVA analysis with Tukey’s post hoc test was used (flow cytometry, gene expression levels in RCAS-induced glioma, U87pcDNA, or IL-33 migration assay). For correlation analyses, *χ*^2^ test (frequency tables of immunohistochemistry TMA data), Pearson’s correlation analysis (correlation between IL-33 expression in BTIC lines and corresponding primary tumors), and Wilcoxon rank-sum test (correlations between IL-33 expression and M1 macrophage markers, or IL-33 expression and M2 macrophage markers in TCGA GBM patients in box plots) were used. For all Kaplan–Meier survival curves, the log-rank Mantel–Cox test was used.

### Reporting summary

Further information on research design is available in the Nature Research Reporting Summary linked to this article.

## Supplementary information

Supplementary Information

Descriptions of Additional Supplementary Files

Supplementary Data 1

Reporting Summary

## Data Availability

The raw RNA sequencing data of BTIC cultures and primary GBM specimens were obtained from the EGA under the study accession number EGAS00001002709. Microarray gene expression data of IL-33-expressing cell clones are deposited at the GEO under accession number GSE153486. Raw mRNA expression data and GBM survival outcomes were obtained from the Oncomine database (https://www.oncomine.org/) and Repository of Molecular Brain Neoplasia Data (REMBRANDT) of the National Cancer Institute (http://www.betastasis.com/). The raw NanoString nCounter gene expression data of cytokines are deposited at the GEO under accession number GSE153488. Microarray gene expression data of PDGFA-, PDGFB-, and shNF-1-induced murine glioma were obtained from GEO under the accession number GSE45874. The phosphoproteomic mass spectrometry data for IL-33^−^ and IL-33^+^ xenografts is deposited at the ProteomeXchange Consortium^167^ under accession number PXD021100. The raw and processed scRNAseq data of immune cells from IL-33^−^ and IL-33^+^ xenografts are deposited at the GEO under accession number GSE153487. Source data underlying Figs. 1c, d, 2c, 3d, e, g, 4c, 5c, e, g, 6d, 7a–c, 8f, 9b, c and 10c, and Supplementary Figs. 1a, 2, 3d–g, 4a, 5, 6b, 6c and 7b is provided in the Source data file. All remaining relevant data are available in the article, Supplementary Information, or from the corresponding authors upon reasonable request.

## Source data

Source Data

## Code availability

Computer code for analysis of immune cells from IL-33^−^ and IL-33^+^ xenografts can be found at https://github.com/menon-s/De-Boeck-Ahn-et-al-IL-33.
