# Supplementary material for: Glioma-derived IL-33 orchestrates an inflammatory brain tumor microenvironment that accelerates glioma progression
Source: Nat Commun. 2020 Oct 5;11:4997. doi: 10.1038/s41467-020-18569-4 (PMC7536425; doi:10.1038/s41467-020-18569-4)
Supplement: Supplementary file 1 — Supplementary Information [file 41467_2020_18569_MOESM1_ESM.pdf]

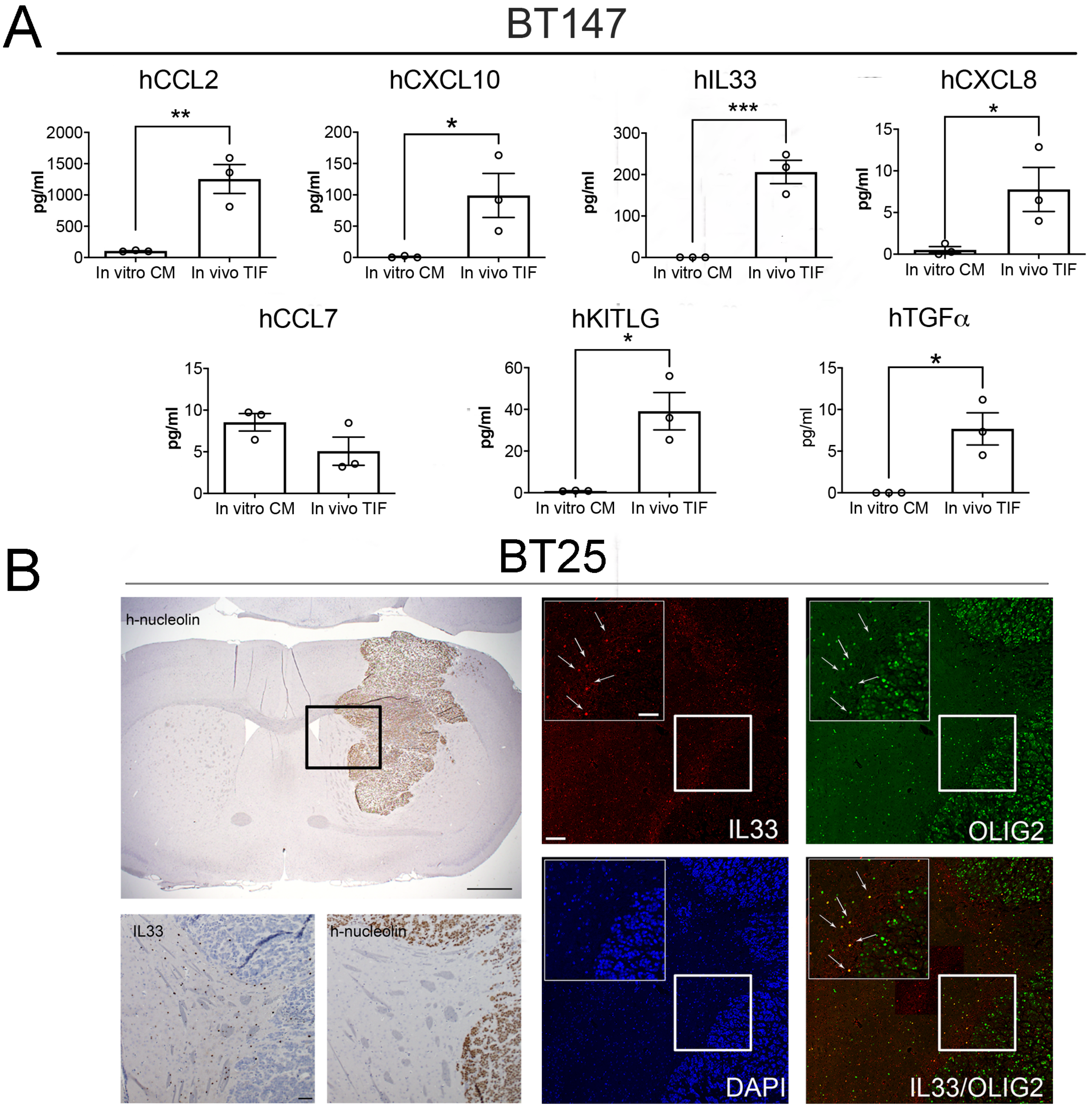

Supplementary Figure 1; Related to Figure 1.
IL-33 is a component of the inflammatory secretome within glioblastoma.

(A) Cytokine levels were measured in conditioned media (CM) from BT147 cells grown *in vitro* and the tumor interstitial fluid (TIF) from BT147 intracranial xenografts using human 65-plex and mouse 32-plex Luminex arrays. Only cytokines that were significantly upregulated in BT147 as compared to BT25 xenografts (see Figure 1C) are shown. Data are the mean ± SEM from three replicates in two independent experiments. $*p \leq .05$, $**p \leq .01$, $***p \leq .001$ by two-sided unpaired Student's t-test (hCCL2 p=0.007; hCXCL10 p=0.049; hIL33 p=0.001; hCXCL8 p=0.053; hKITLG p=0.013; hTGFα p=0.016). (B) Immunohistochemical analysis (IHC) of IL-33 (brown) and human nucleolin (h-nucleolin, brown) in BT25 xenografts. Sections were counterstained with hematoxylin (blue). IL-33 expressing cells in normal brain tissue surrounding the tumor region are visible. Upper left panel scale bar: 2 mm; lower left panel scale bar: 30 µm (N=5). BT25 xenografts were stained using multiplex immunofluorescence for IL-33 (red), the oligodendrocyte transcription factor Olig-2 (green) and DAPI (blue) to visualize all nuclei (right panel). Shown are representative images showing co-localization of IL-33 with oligodendrocytes in the normal brain tissue surrounding the tumor region (white arrows), scale bar: 120 µm; inset scale bar: 60 µm.

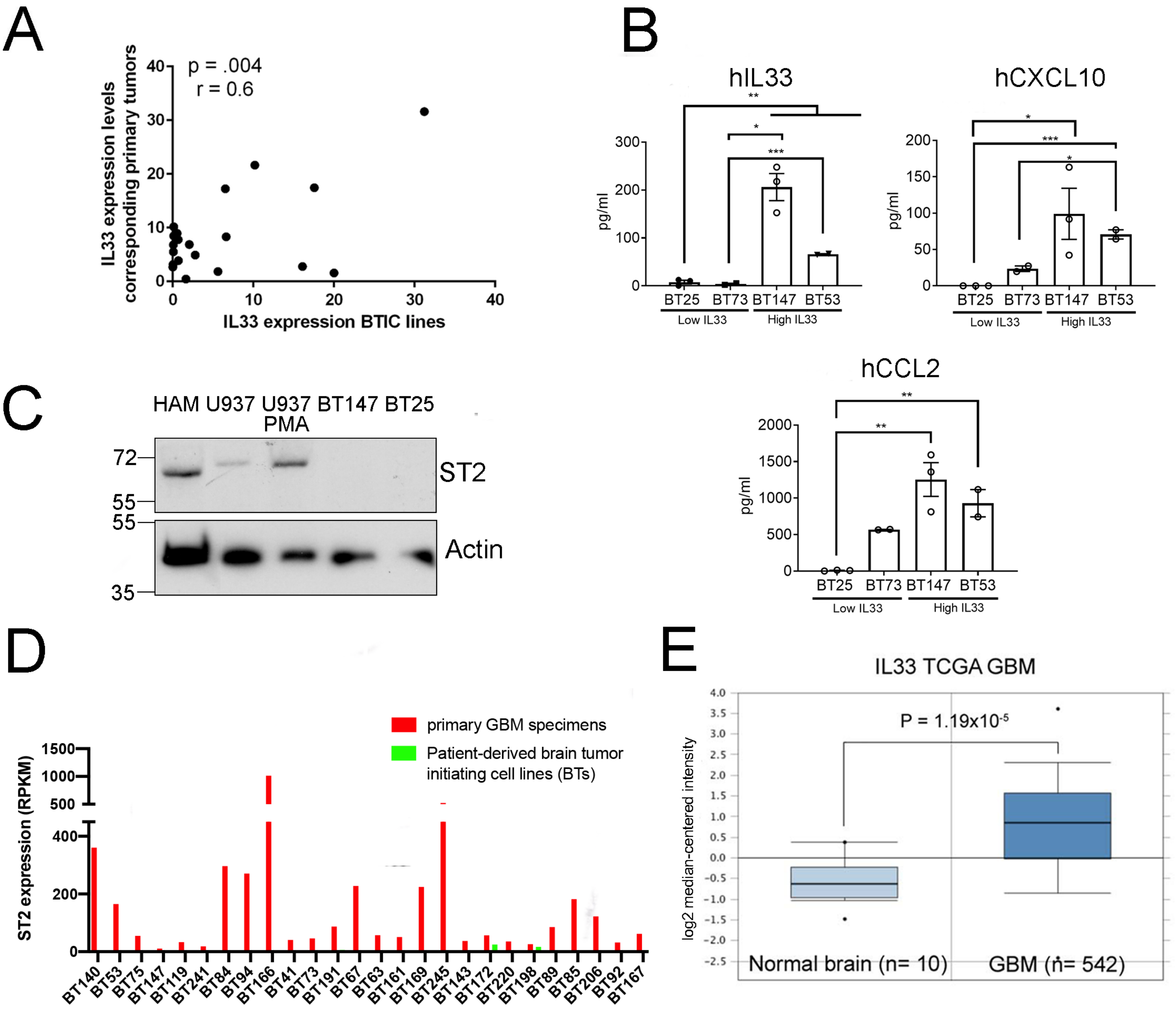

Supplementary Figure 2; Related to Figure 2.

IL-33 is expressed in a subset of GBMs and correlates with TAM density.

(A) Graph shows correlation between IL-33 expression in patient-derived brain tumor initiating lines and corresponding primary tumor specimens. Pearson's correlation coefficient R and correlation *p*-value are shown. (B) Expression of human IL-33, CXCL10 and CCL2 in the interstitial fluid of non-inflammatory BT25 and BT73, and inflammatory BT53 and BT147 intracranial xenografts. Data are mean ±SEM in four replicates from three independent experiments. **p* ≤ .05, ***p* ≤ .01, ****p* ≤ .001, *****p* ≤ .0001 by one-way ANOVA with Tukey's post-hoc test. hIL33 (BT147 vs BT25 *p*=0.002; BT53 vs BT25, *p*=0.001; BT53 vs BT73, *p*=0.001; BT147 vs BT73, *p*=0.011) hCXCL10 (BT147 vs BT25, *p*=0.047; BT53 vs BT25, *p*< 0.001; BT53 vs BT73, *p*=0.023); hCCL2 (BT147 vs BT25, *p*=0.005; BT53 vs BT25, *p*=0.007). (C-D) Macrophage but not patient-derived BTICs express the IL-33-receptor ST2. (C) Human adult microglia (HAM), U937, U937 macrophage treated with phorbol 12-myristate 13-acetate (PMA) and BTICs BT25 and BT147 were assessed for ST2 by Western. N=3 experiments with comparable results. (D) RNA-seq was performed on 35 patient-derived BTICs and 26 primary GBM specimens. Graph shows RNA expression (per million mapped reads; RPKM) of ST2 in BTICs and corresponding primary GBM tissue specimens from which the BTICs were isolated. Although ST2 expression was observed in some primary patient samples, little or no expression of ST2 was detected in the patient-derived glioma cells, an observation consistent with ST2-expressing macrophage/ microglia within the tumor microenvironment. (E) Analysis of TCGA data indicates that IL-33 is expressed at significantly higher levels in GBM tissue compared to normal brain tissue (TCGA database at https:// www.oncomine.org). The *p* value was based on a two-sided unpaired Student's t-test. Boxplots indicate the median (center line), 25th and 75th percentiles (boundary of box), 10th and 90th percentiles (whiskers) and minimum and maximum values (dots).

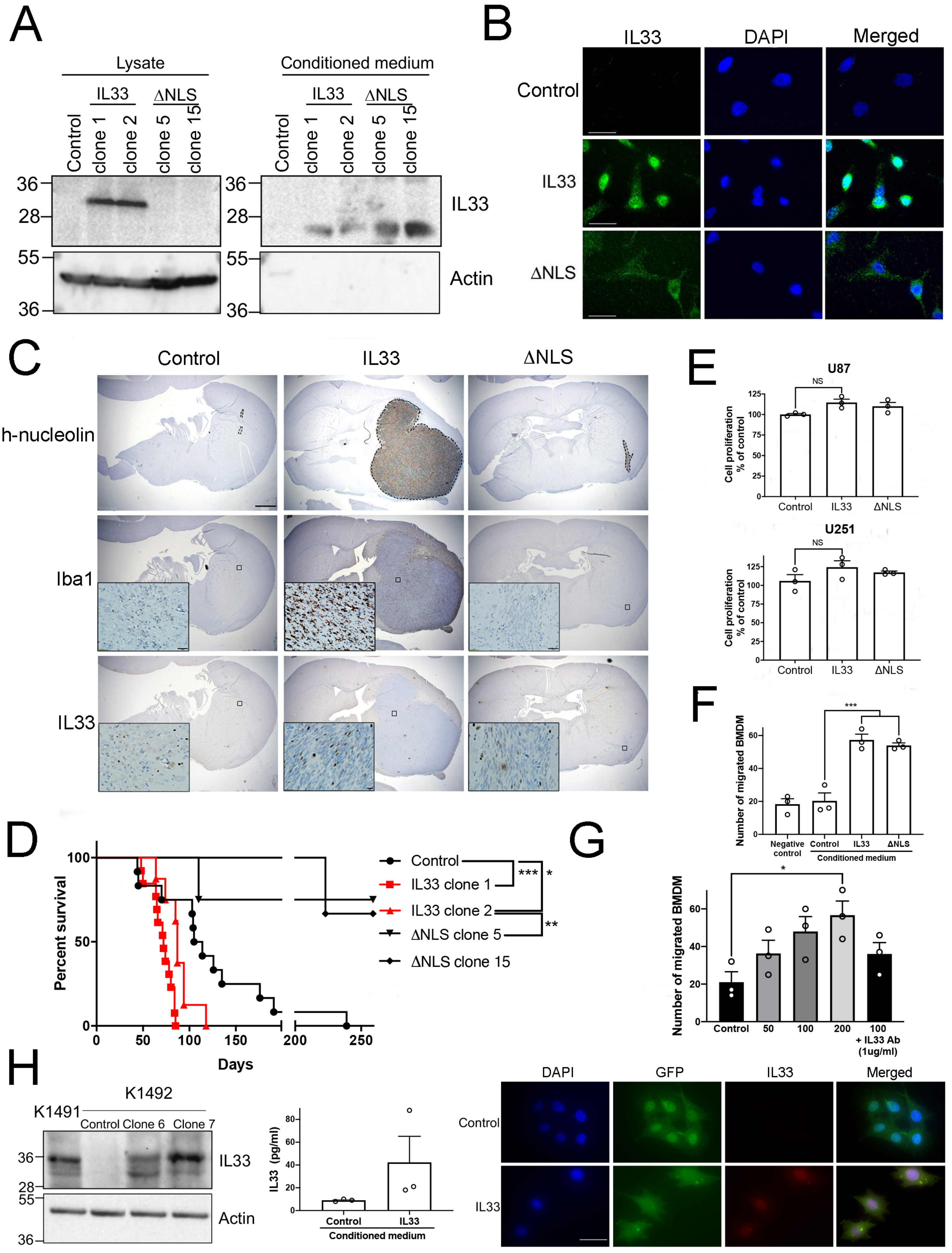
A
Lysate
Conditioned medium
IL33
ΔNLS
Control
clone 1
clone 2
clone 5
clone 15
IL33
Actin
B
IL33
DAPI
Merged
Control
IL33
ΔNLS
C
Control
IL33
ΔNLS
h-nucleolin
Iba1
IL33
D
Percent survival
Days
Control
IL33 clone 1
IL33 clone 2
ΔNLS clone 5
ΔNLS clone 15
E
U87
U251
NS
Cell proliferation % of control
Control
IL33
ΔNLS
F
Number of migrated BMDM
Negative control
Control
IL33
ΔNLS
Conditioned medium
G
Number of migrated BMDM
Control
50
100
200
100 + IL33 Ab (1ug/ml)
H
K1491
K1492
Control
Clone 6
Clone 7
IL33
Actin
IL33 (pg/ml)
Control
IL33
Conditioned medium
DAPI
GFP
IL33
Merged
Control
IL33

Supplementary Figure 3; Related to Figure 3.
Nuclear IL-33 is required to promote tumor growth and macrophage infiltration.
(A) Representative IL-33 Western of U251 IL-33 (clones 1 and 2) and ΔNLS (clones 5 and 15) detect full-length IL-33 (33kDa) in the cell lysates and a 25 kDa secreted form in the conditioned medium. U251 pcDNA vector (Control) was used as a control. Actin was used as a loading control. N=3 experiments. **(B)** Representative immunofluorescent images of U251 pcDNA (Control), IL-33 (IL33) or ΔNLS 24 hr following treatment with Leptomycin B (50 nM). DAPI (blue) was used to visualize cell nuclei. Scale bar, 20 μm. **(C)** Mice bearing IL-33^{-} (pcDNA; Control), IL-33^{+} (IL33) and ΔNLS U251 were sacrificed at 5 weeks, when mice bearing IL-33 tumors became symptomatic. FFPE-sections were stained (brown) for human nucleolin (h-nucleolin), Iba1 or IL-33 and counterstained with hematoxylin (blue). Scale bar: 2mm; inset scale bar: 25μm. **(D)** Kaplan-Meier survival curve of animals bearing U251 Control (N=12), IL-33 (clone 1 N=13; clone 2 N=8) and ΔNLS (clone 5 N=4; clone 15 N=3) tumors from three independent experiments. **p* ≤ .05, ***p* ≤ .01, ****p* ≤ .001 was calculated using log-rank Mantel-Cox test (Control vs IL-33 clone 1, *p*=0.0009; vs clone 2, *p*=0.0298; IL-33 clone 2 vs ΔNLS clone 5, ***p*=0.0013). **(E)** Representative Alamar Blue cell proliferation assay (72 hr) for U87 (upper graph) and U251 (lower graph). Data are mean ±SEM from 3 replicates. N=3 experiments with comparable results. The *p* value was calculated using one-way ANOVA with Tukey's post-hoc test. **(F)** BMDM were treated with CM from U87 control (pcDNA), IL-33 (IL33) or ΔNLS and assessed for migration at 4 hours. Media was added as a negative control. Data are the mean ±SEM of 3 replicates. N=3 experiments with comparable results. ****p* ≤ .001 by one-way ANOVA with Tukey's post-hoc test **(**Control CM vs IL-33 CM, *p*=0.0003; vs ΔNLS CM, *p*=0.0006). **(G)** Graphs show migration of BMDM treated with recombinant-IL-33 (rIL33, ng) in the presence or absence of anti-IL-33. PBS was used as a negative control (control). Data are the mean ±SEM of 3 replicates. N=3 experiments with comparable results. **p* ≤ .05 by one-way ANOVA with Tukey's post-hoc test (Control vs 200 ng/ml of rIL-33, *p*=0.028). **(H)** Representative IF (right) for nuclear IL-33 (green) in K1492 Control or IL-33 cells 24 hr after treatment with Leptomycin B (50 nM). Scale bar: 20 μm. IL-33 Western detected full-length IL-33 (33kDa) in the cell lysates of IL-33 transfected K1492 (clones 6 and 7). IL-33 positive K1491 was used for comparison. Actin was used as a loading control. Secreted IL-33 was detected in conditioned medium from K1492 IL-33 using Luminex. N=3 experiments.

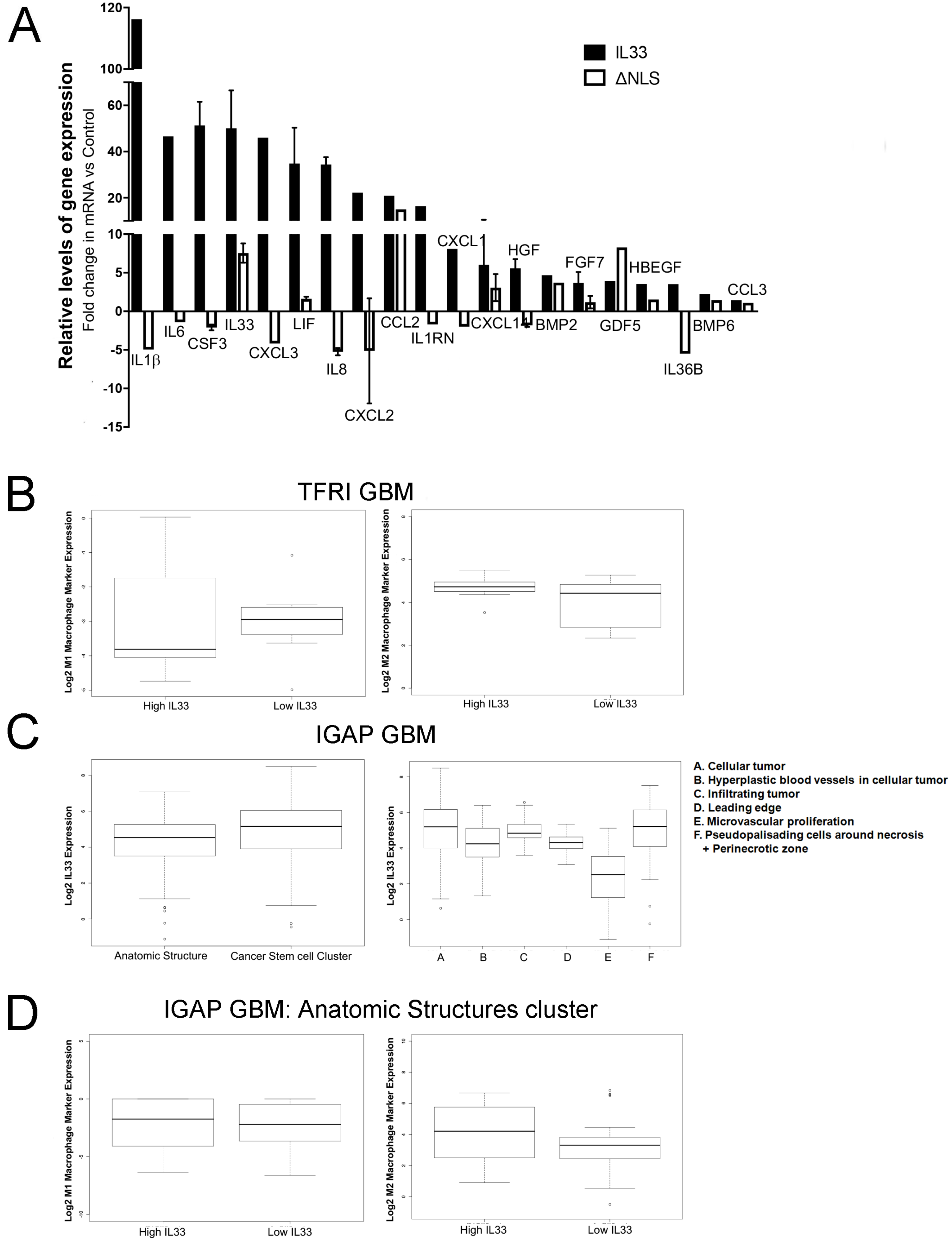
A
IL33
ΔNLS
Relative levels of gene expression
Fold change in mRNA vs Control
120
100
60
40
20
10
5
0
-5
-10
-15
IL1β
IL6
CSF3
IL33
CXCL3
LIF
IL8
CXCL2
CCL2
IL1RN
CXCL1
CXCL14
HGF
BMP2
FGF7
GDF5
HBEGF
IL36B
BMP6
CCL3
B
TFRI GBM
Log2 M1 Macrophage Marker Expression
Log2 M2 Macrophage Marker Expression
High IL33
Low IL33
C
IGAP GBM
Log2 IL33 Expression
Anatomic Structure
Cancer Stem cell Cluster
A
B
C
D
E
F
A. Cellular tumor
B. Hyperplastic blood vessels in cellular tumor
C. Infiltrating tumor
D. Leading edge
E. Microvascular proliferation
F. Pseudopalisading cells around necrosis
+ Perinecrotic zone
D
IGAP GBM: Anatomic Structures cluster
Log2 M1 Macrophage Marker Expression
Log2 M2 Macrophage Marker Expression
High IL33
Low IL33

Supplementary Figure 4; Related to Figure 5.
Nuclear IL-33 mediates the release of inflammatory cytokines from glioma cells.
(**A**) Graph shows the fold change of inflammatory genes in IL-33 or ΔNLS-expressing U87 glioma cells as compared to the U87 control. Data are mean ±SEM when 3 or more probe sets for a given gene are detected. (**B**) Box plots show correlation between either *IL-33* expression and M1 macrophage markers or IL-33 expression and M2 macrophage markers from the TFRI GBM patient cohort. M1 macrophage high *IL-33* expression is defined as top 25% of sample size (minima: -4.74; maxima: 0.03; centre: -3.81; first quartile: -4.26; third quartile: -1.04). M1 macrophage low *IL-33* expression defined by bottom 25% of sample size (minima: -4.99; maxima: -1.08; centre: -2.94; first quartile: -3.63; third quartile:-2.52). M2 high *IL-33* expression is defined as top 25% of sample size (minima: 3.53; maxima: 5.51; centre: 4.73; first quartile: 4.37; third quartile: 5.15). M2 macrophage low *IL-33* expression is defined as bottom 25% of sample size (minima: 2.33; maxima: 5.28; centre: 4.43; first quartile: 2.50; third quartile: 4.96). (**C**) Boxplots show the relative expression of *IL-33* in anatomic structures and stem cell clusters of the IVY GBM Atlas cohort (IGAP). Anatomic structures (minima: -1.11; maxima: 7.08; centre: 4.54; first quartile: 3.50; third quartile: 5.27. Cancer Stem Cell cluster defined by minima: -0.45; maxima: 8.49; centre: 5.15; first quartile: 3.90; third quartile: 6.05); Cellular tumor cluster (minima: 0.62; maxima: 8.49; centre: 5.20; first quartile: 3.91; third quartile: 6.17); Hyperplastic blood vessels (minima: 1.32; maxima: 6.40; centre: 4.2; first quartile: 3.48; third quartile: 5.17); Infiltrating tumor (minima: 3.60; maxima: 6.56; centre: 4.84; first quartile: 4.57; third quartile: 5.36. Leading edge cluster defined by minima: 3.08; maxima: 5.34; centre: 4.31; first quartile: 3.78; third quartile: 4.63); Microvascular proliferation (minima: -1.12; maxima: 5.12; centre: 2.51; first quartile: 1.22; third quartile: 3.55); Pseudopalisading/perinecrotic (minima: -0.25; maxima: 7.51; centre: 5.21; first quartile: 4.09; third quartile: 6.14). (**D**) Box plots show a correlation between either *IL-33* expression and M1 macrophage markers or *IL-33* expression and M2 macrophage markers in non-stem cell anatomic structures of the IVY GBM Atlas program cohort (IGAP). M1 macrophage high *IL-33* expression is defined as top 25% of sample size (minima: -6.35; maxima: 0; centre: -1.74; first quartile: -4.14; third quartile: 0). M1 macrophage low *IL-33* expression is defined as bottom 25% of sample size (minima: -6.61; maxima: 0; centre: -2.20; first quartile: -3.72; third quartile: -0.35). M2 high *IL-33* expression defined as top 25% of sample size (minima: 0.91; maxima: 6.67; centre: 4.21; first quartile: 2.38; third quartile: 5.78). M2 macrophage low *IL-33* expression is defined as bottom 25% of sample size (minima: -0.50; maxima: 6.83; centre: 3.31; first quartile: 2.38; third quartile: 3.87).

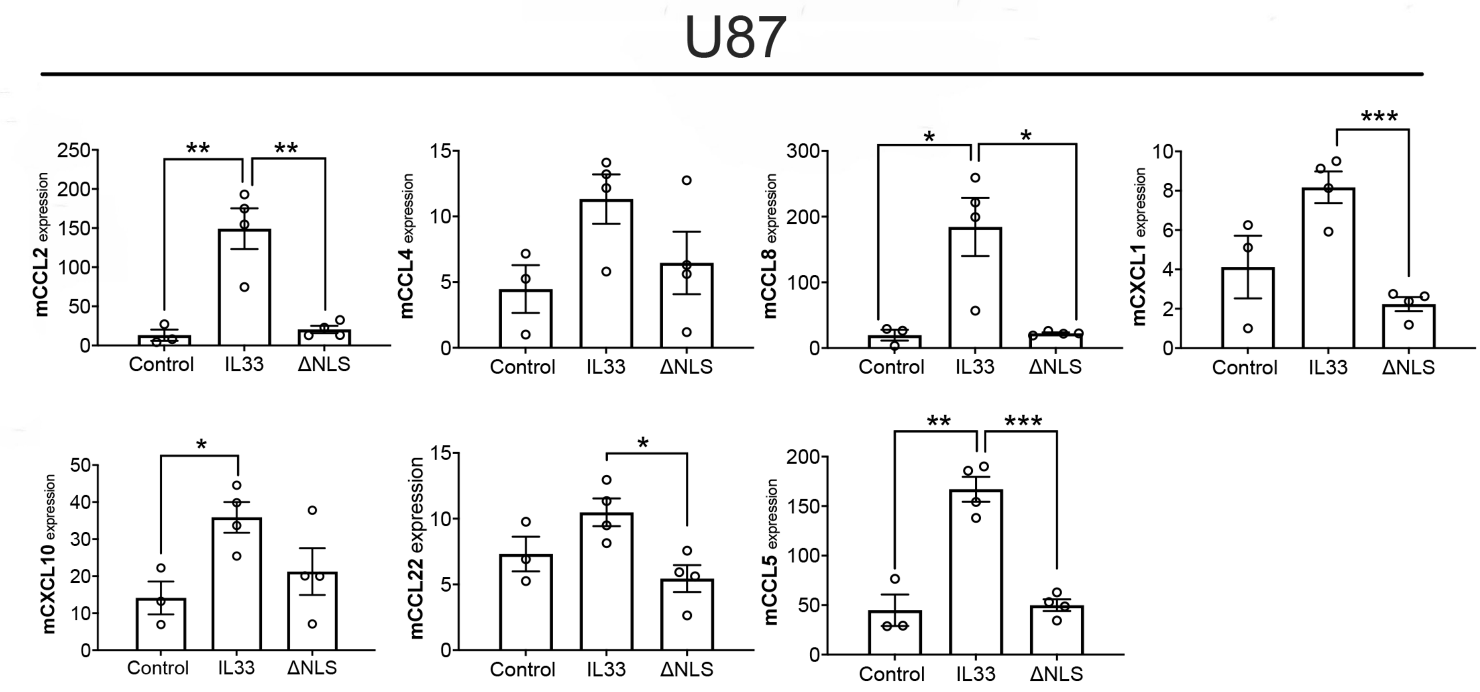

Supplementary Figure 5; Related to Figure 5.

Nuclear IL-33 is required for the recruitment of M2 pro-tumorigenic macrophages.

NanoString nCounter gene expression analysis using the nCounter Mouse v2 Inflammation Panel showing increased expression of inflammatory cytokines (CCL2, CCL4, CCL8, CXCL1, CXCL10, CCL22, CCL5) in xenografts from IL-33− (Control, N=3), IL-33+ (IL33, N=4) or ΔNLS (N=4) U87-tumor bearing mice, 1 week after tumor implantation. Data are mean ± SEM from three or four replicates. *$p \leq .05$, **$p \leq .01$, ***$p \leq .001$, by one-way ANOVA with Tukey's post-hoc test. mCCCL2 (IL-33 vs pcDNA, p=0.007; ΔNLS vs IL-33, p=0.002) mCCL8 (IL-33 vs pcDNA, p=0.026; ΔNLS vs IL-33, p=0.010) mCXCL1 (IL-33 vs pcDNA, p=0.056; ΔNLS vs IL-33, p<0.001) mCXCL10 (IL-33 vs pcDNA, p=0.016) mCCL22 (ΔNLS vs IL-33, p=0.014) mCCL5 (IL-33 vs pcDNA, p=0.001; ΔNLS vs IL-33 p<0.001).

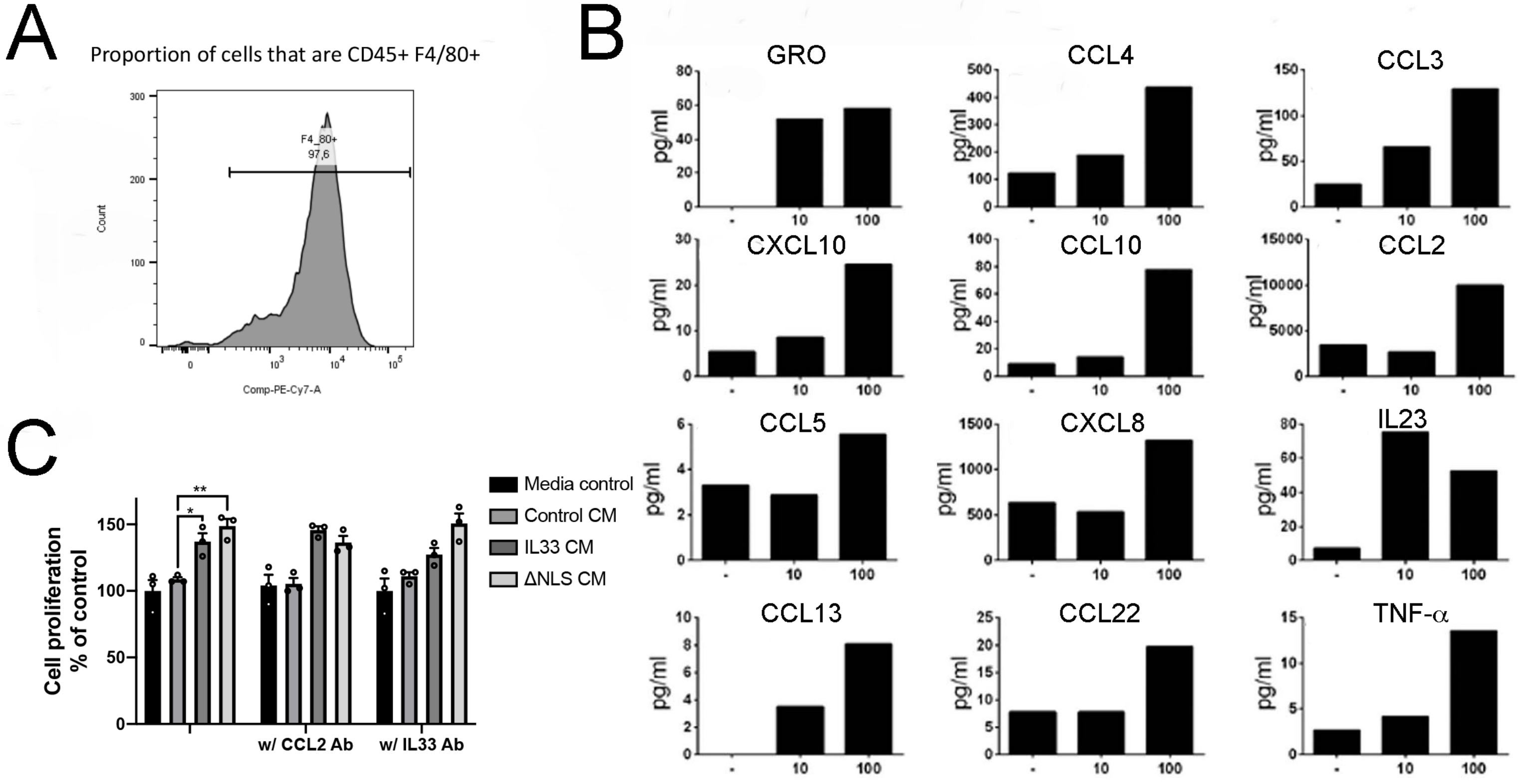

Supplementary Figure 6; Related to Figure 7.
IL-33 mediates direct effects on BMDM and microglia.
(A) F4/80 histogram shows over 97% enrichment of differentiated BMDM. (B) Graphs show a dose-dependent increase in the secretion of inflammatory cytokines from human fetal microglia in response to rIL-33 (0-100 pg/ml) as measured using a human 65-plex Luminex array. (C) Graphs show representative cell proliferation of BMDM treated with CM from control, IL-33 or ΔNLS expressing U87 glioma cells in the absence or presence of CCL2 or IL-33 neutralizing antibodies. Data are the mean ±SEM from three replicates. The experiment was repeated three times with comparable results. *$p \leq .05$, **$p \leq .01$ by one-way ANOVA with Tukey's post-hoc test (Control CM vs IL-33 CM, p=0.038; vs ΔNLS CM, p= 0.006).

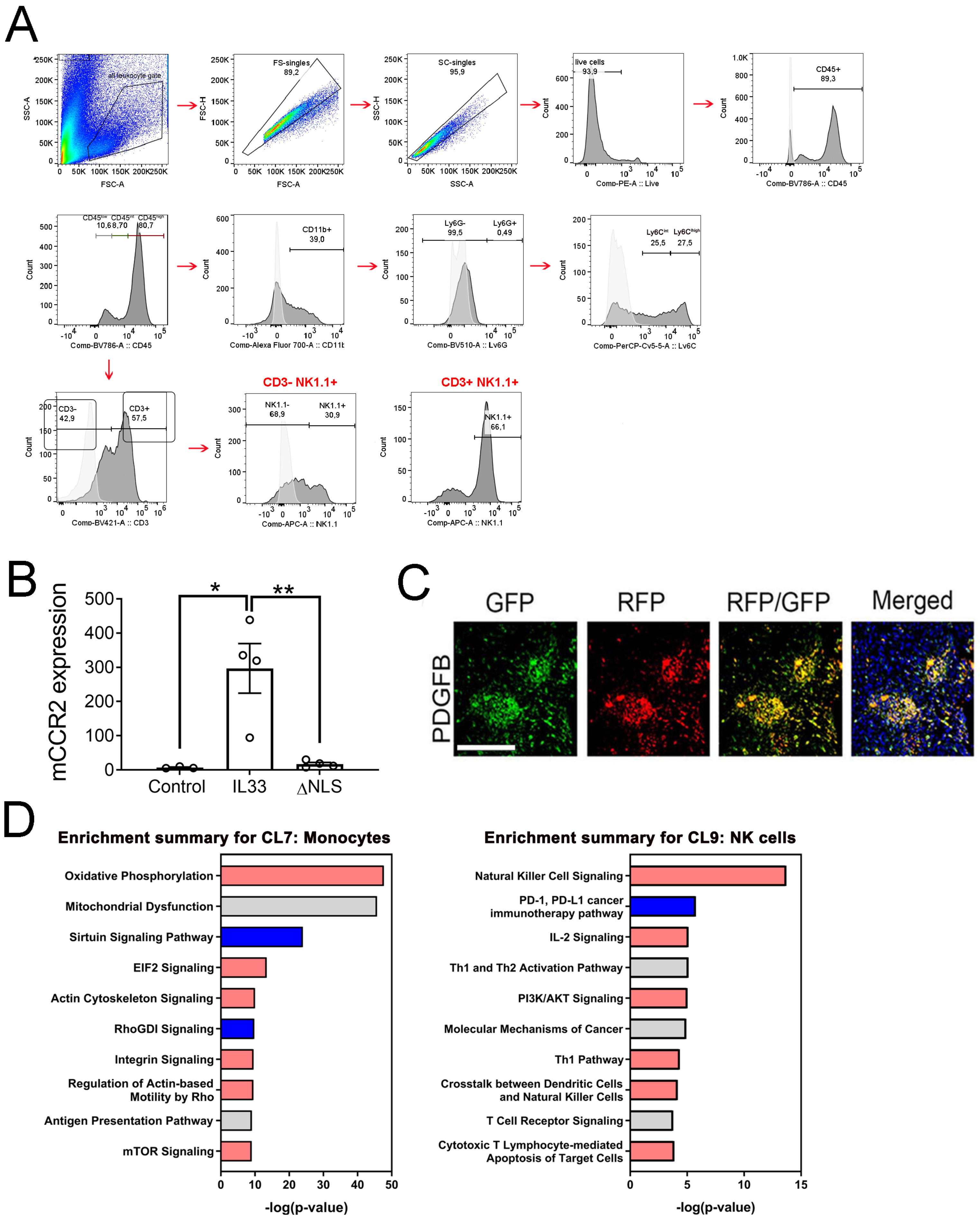
A
all leukocyte gate
FS-singles 89,2
SC-singles 95,9
live cells 93,9
CD45+ 89,3
SSC-A
FSC-A
FSC-H
SSC-H
Count
Comp-PE-A :: Live
Comp-BV786-A :: CD45
CD11b+ 39,0
Comp-Alexa Fluor 700-A :: CD11b
Ly6G- 99,5
Ly6G+ 0,49
Comp-BV510-A :: Ly6G
25,5
27,5
Comp-PerCP-Cy5-5-A :: Ly6C
CD3- 42,9
CD3+ 57,5
Comp-BV421-A :: CD3
CD3- NK1.1+
NK1.1- 68,9
NK1.1+ 30,9
Comp-APC-A :: NK1.1
CD3+ NK1.1+
NK1.1+ 66,1
B
mCCR2 expression
Control
IL33
ΔNLS
C
GFP
RFP
RFP/GFP
Merged
PDGFB
D
Enrichment summary for CL7: Monocytes
Oxidative Phosphorylation
Mitochondrial Dysfunction
Sirtuin Signaling Pathway
EIF2 Signaling
Actin Cytoskeleton Signaling
RhoGDI Signaling
Integrin Signaling
Regulation of Actin-based Motility by Rho
Antigen Presentation Pathway
mTOR Signaling
-log(p-value)
Enrichment summary for CL9: NK cells
Natural Killer Cell Signaling
PD-1, PD-L1 cancer immunotherapy pathway
IL-2 Signaling
Th1 and Th2 Activation Pathway
PI3K/AKT Signaling
Molecular Mechanisms of Cancer
Th1 Pathway
Crosstalk between Dendritic Cells and Natural Killer Cells
T Cell Receptor Signaling
Cytotoxic T Lymphocyte-mediated Apoptosis of Target Cells
-log(p-value)

Supplementary Figure 7; Related to Figure 9.

Flow cytometry gating strategy for microglia and infiltrating immune cells.

(A) Plots shown are representative data from K1492-IL-33 tumor-bearing mice. Similar gating strategy was used to assess immune cells in all Sham and glioma models described in this study. Upper top left plot shows the leukocyte gate used. The red arrows show the progression of the gating strategy used with description of the population of interest indicated in the dot profile or histogram. Doublets were excluded using forward and side scatter profiles. Live vs. dead cells were determined using propidium iodide (PI) stain. The histogram shows the proportion of cells within the leukocyte gate that are PI negative (i.e. live). The histogram on the right shows the proportion of live cells that are $CD45^{+}$. In subsequent histogram plots, light grey represents the isotype control for the respective fluorochrome. The histogram on the far left second row shows the distribution of $CD45^{low}$, $CD45^{int}$ and $CD45^{high}$ cells. Next on the right, the histogram shows the gating of $CD11b^{+}$ cells within the $CD45^{high}$ population (i.e. infiltrating myeloid cells), followed by the distribution of $Ly6G^{+}$ neutrophils within the $CD45^{high}CD11b^{+}$ gate and the distribution of $Ly6C^{high}$ and $Ly6C^{int}$ monocytes within the $Ly6G^{-}CD11b^{+}CD45^{high}$ population. Far left histogram third row shows distribution of $CD3^{+}$ cells within the $CD45^{high}$ population, followed by the NK1.1 expression within the $CD3^{+}$ population. $CD3^{+}NK1.1^{+}$ cells were identified as NKT cells, $CD3^{+}NK1.1^{-}$ cells were identified as $CD3^{+}$ T cells and $CD3^{-}$ $NK1.1^{+}$ cells were identified as NK cells. Far right histogram shows distribution of $CD19^{+}$ B cells within the $CD45^{high}$ population. **(B)** Bar graph shows the mean ±SEM of CCR2 expression as determined by NanoString nCounter platform in IL-33^{-} (Control; N=3), IL-33^{+} (IL33; N=4) and ΔNLS (N=4) U87 xenografts. *p <0.05, **p < 0.01 by one-way ANOVA with Tukey's post-hoc test (Con vs IL- 33, p=0.0197; ΔNLS vs IL-33, p=0.0085). **(C)** Immunofluorescent images of brain sections from PDGFB driven tumors in $CX3CR1^{GFP/wt}/CCR2^{RFP/wt}$ reporter mice where microglia ($CX3CR1^{high,}$ $CCR2^{-}$; green) are distinguished from monocyte-derived macrophages ($CX3CR1^{+}CCR2^{+}$; green and red). (N=3 mice/group). (**D**) Canonical pathways enriched in cluster 7 monocytes (right panel) and cluster 9 NK cells (left panel) from the IL-33+ xenograftsas determined using Ingenuity Pathway Analysis (IPA) which predicts pathways as activated (red, positive z-score), inactivated (blue, negative z-score), or no activity pattern available (grey).

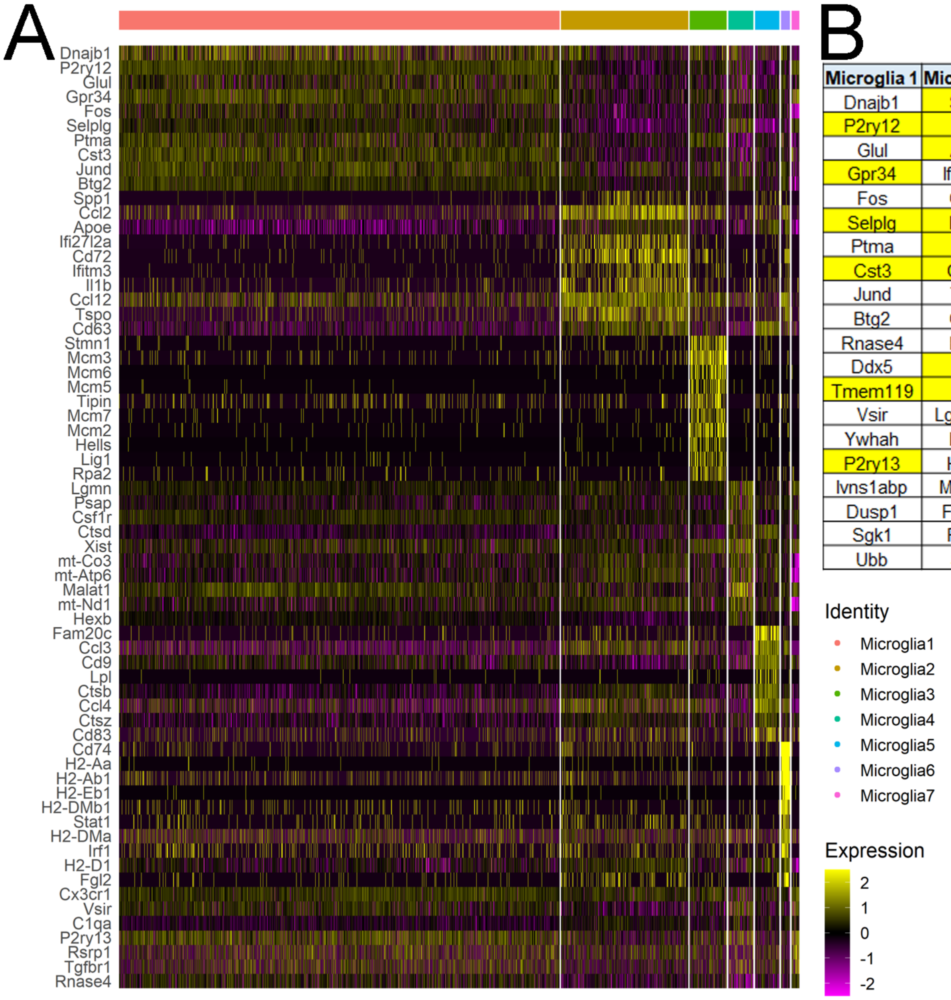

B

Microglia 1	Microglia 2	Microglia 3	Microglia 4	Microglia 5	Microglia 6	Microglia 7
Dnajb1	Spp1	Stmn1	Lgmn	Fam20c	Cd74	P2ry12
P2ry12	Ccl2	Mcm3	Psap	Ccl3	H2-Aa	Gpr34
Glul	Apoe	Mcm6	Csf1r	Cd9	H2-Ab1	Cx3cr1
Gpr34	Ifi27l2a	Mcm5	Ctsd	Lpl	H2-Eb1	Vsir
Fos	Cd72	Tipin	Xist	Ctsb	H2-DMb1	C1qa
Selplg	Ifitm3	Mcm7	mt-Co3	Cd63	Stat1	Selplg
Ptma	Il1b	Mcm2	mt-Atp6	Ccl4	H2-DMa	P2ry13
Cst3	Ccl12	Hells	Malat1	Ctsd	Irf1	Rsrp1
Jund	Tspo	Lig1	mt-Nd1	Ctsz	H2-D1	Tgfbr1
Btg2	Cd63	Rpa2	Hexb	Cd83	Fgl2	Rnase4
Rnase4	Pim1	Mcm4	mt-Cytb	Lyz2	Ly6e	Rrbp1
Ddx5	Ccl3	Ezh2	mt-Nd4	Cadm1	B2m	Arhgap45
Tmem119	Lyz2	Fen1	mt-Co1	Nceh1	H2-DMb2	Ifngr1
Vsir	Lgals3bp	Lmnb1	Vsir	Lgals1	H2-K1	P2ry6
Ywhah	Naaa	Rrm1	Unc93b1	Bcl2a1b	H2-T23	Cyth4
P2ry13	Hcar2	Fam111a	Pld4	Cd68	H2-Q7	Hist1h1e
Ivns1abp	Ms4a6c	Dnajc9	Selplg	Gadd45b	Fcgr4	Ccnt2
Dusp1	Fcgr2b	Cdca7	Tmem119	Apoe	Tap1	Ccr5
Sgk1	Rpl32	Tmpo	Ctsa	Fth1	C1qb	Xist
Ubb	Slfn2	Tubb5	Grn	Axl	Psmb9	Mef2c

*Yellow highlights indicate genes that have previously been identified in the literature as part of a microglia cell state signature (see Suplementary Table 7)

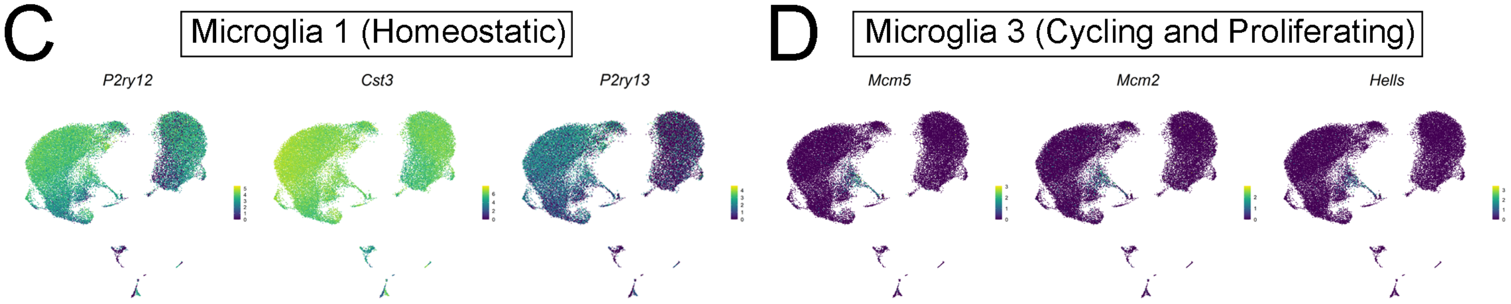

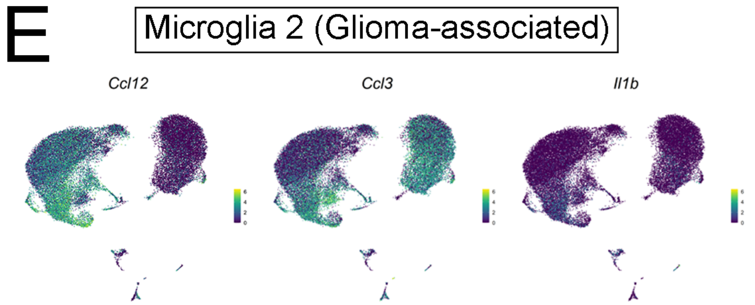

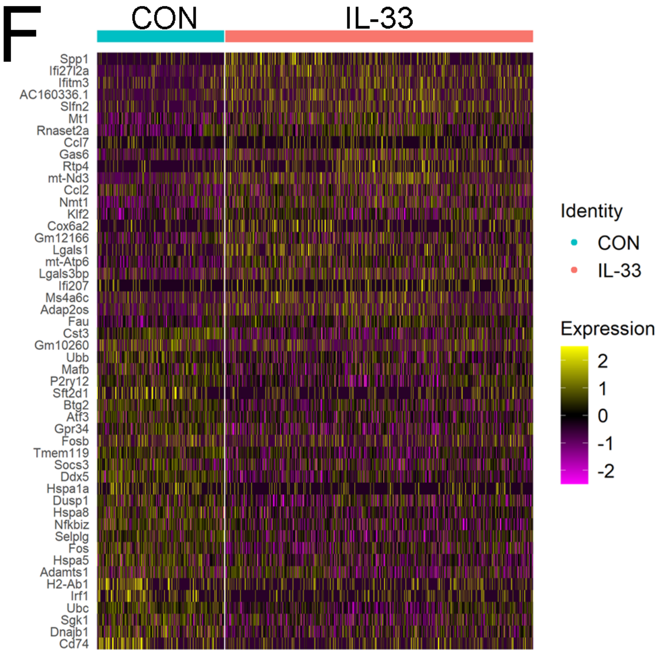

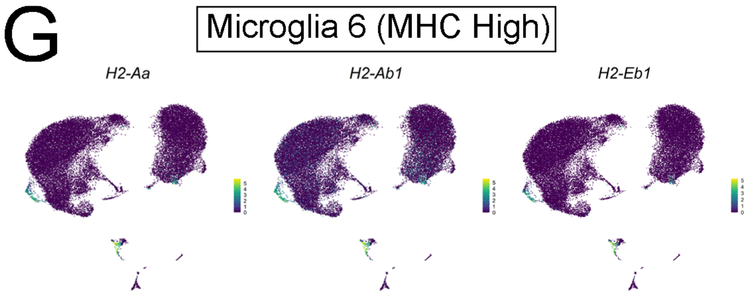

Supplementary Figure 8; Related to Figure 10. Microglial cell states by single cell sequencing.
(A) Heatmap shows the top 10 differentially expressed genes in Microglia 1-7. Differentially expressed genes were determined using a Wilcoxon ranked sum test comparing each cluster to all other microglial clusters. Genes were ordered by Bonferroni adjusted *p*-value. **(B)** Table shows the top 20 differentially expressed genes assessed in A. **(C-E, G)** UMAP plots show the expression of top differentially expressed genes in Microglia 1 (homeostatic microglia), Microglia 3 (cycling and proliferating microglia), Microglia 2 (glioma-associated microglia), and Microglia 6 (MHC high microglia), respectively. **(F)** Heatmap depicts differentially expressed genes in Microglia 2 from control (CON; blue) and IL-33-expressing (red) glioma xenografts. Differentially expressed genes were determined using a Wilcoxon ranked sum test and ordered by average log fold-change.

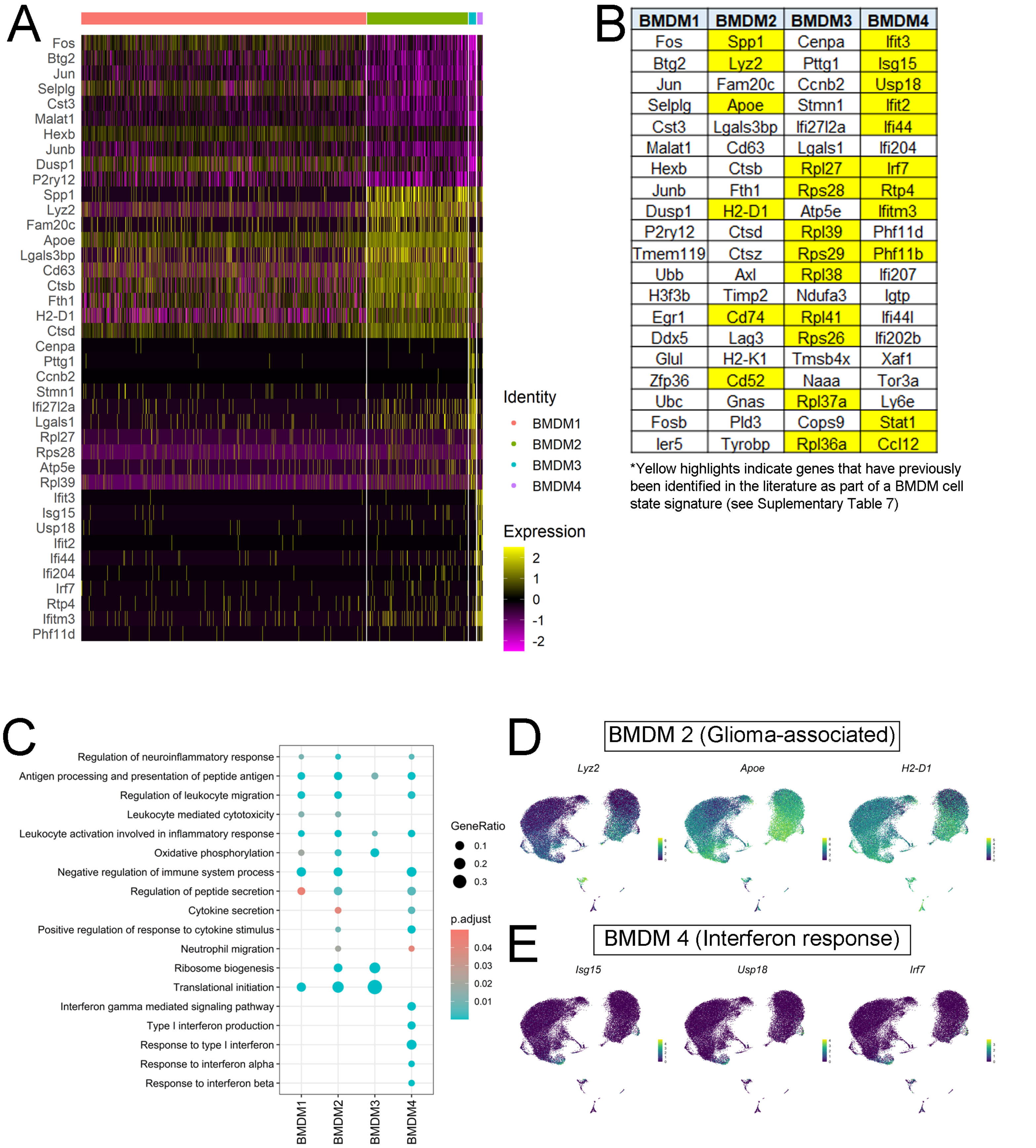

BMDM1	BMDM2	BMDM3	BMDM4
Fos	Spp1	Cenpa	Ifit3
Btg2	Lyz2	Pttg1	Isg15
Jun	Fam20c	Ccnb2	Usp18
Selplg	Apoe	Stmn1	Ifit2
Cst3	Lgals3bp	Ifi27l2a	Ifi44
Malat1	Cd63	Lgals1	Ifi204
Hexb	Ctsb	Rpl27	Irf7
Junb	Fth1	Rps28	Rtp4
Dusp1	H2-D1	Atp5e	Ifitm3
P2ry12	Ctsd	Rpl39	Phf11d
Tmem119	Ctsz	Rps29	Phf11b
Ubb	Axl	Rpl38	Ifi207
H3f3b	Timp2	Ndufa3	Igtp
Egr1	Cd74	Rpl41	Ifi44l
Ddx5	Lag3	Rps26	Ifi202b
Glul	H2-K1	Tmsb4x	Xaf1
Zfp36	Cd52	Naaa	Tor3a
Ubc	Gnas	Rpl37a	Ly6e
Fosb	Pld3	Cops9	Stat1
Ier5	Tyrobp	Rpl36a	Ccl12

*Yellow highlights indicate genes that have previously been identified in the literature as part of a BMDM cell state signature (see Suplementary Table 7)

Supplementary Figure 9; Related to Figure 10. BMDM cell states by single cell sequencing. (A) Heatmap depicts the top 10 differentially expressed genes in BMDM 1-4 determined using a Wilcoxon ranked sum test comparing each BMDM cluster to all other BMDM clusters. Genes were ordered by Bonferroni adjusted *p*-value. (B) Table shows the top 20 differentially expressed genes assessed in A. (C) Dot plot shows selected enriched gene ontology (GO) terms in BMDM Clusters 1-4 determined using the R package clusterProfiler. Top differentially expressed genes (up to 200) were considered using the genesorteR package with default parameters. Size of dot corresponds to the ratio of genes expressed in the cluster to the total number of genes related to the gene ontology term. Color corresponds to the Benjamini-Hochberg adjusted *p*-value for multiple comparisons for the specific term in each cluster. (D-E) UMAP plots show expression of top differentially expressed genes in BMDM 2 (glioma-associated BMDM) and BMDM 4 (interferon responsive BMDM).

Supplementary Table 1

Gene	Cell Type	Reference
Tmem119	Microglia	[1]Bennet et al., 2016; [2]Haage et al., 2019; [3]Sousa et al., 2018
Gpr34	Microglia	[2]Haage et al., 2019; [3]Sousa et al., 2018
Olfml3	Microglia	[2]Haage et al., 2019; [3]Sousa et al., 2018
P2ry12	Microglia	[2]Haage et al., 2019; [3]Sousa et al., 2018
Cx3cr1	Microglia	[2]Haage et al., 2019; [3]Sousa et al., 2018
Selplg	Microglia	[2]Haage et al., 2019; [3]Sousa et al., 2018
Ly6c2	Monocytes	[4]Ziegler-Heitbrock et al., 2010
Ccr2	Monocytes	[5]Tsou et al., 2007
Gzma	NK Cells	[6]Cursons et al., 2019
Nkg7	NK Cells	[6]Cursons et al., 2019
Ncr1	NK Cells	[6]Cursons et al., 2019; [7]Walzer et al., 2007
Ngp	Granulocytes	[8]Lakschevitz et al., 2015
Camp	Granulocytes	[8]Lakschevitz et al., 2015
S100a9	Granulocytes	[9]Ryckman et al., 2003

Supplementary Table 1: Canonical immune cell markers used for cluster annotation were derived from published data (references indicated in table and Supplementary References).

Supplementary Table 2

Cluster Name	Annotated Signature	Among top 20 DEGs (As compared to the same cell type)	Reference
Microglia1, Microglia7	Homeostatic Microglia	P2ry12, Gpr34, Selplg, Cst3, Tmem119, P2ry13, Cx3cr1	[10]Masuda et al., 2020; [11]Sankowski et al., 2019; [3]Sousa et al., 2018
Microglia2, Microglia5	Glioma Associated Microglia	Spp1, Ccl2, Apoe, Ifitm3, Ilib, Ccl12, Ccl3, Lyz2, Ccl4	[11]Sankowski et al., 2019; [12]Ochocka et al., 2020; [13]Szulzewsky et al., 2015
Microglia3	Cycling and Proliferating Microglia	Mcm5, Mcm2, Hells, Tubb5	[14]Sala Frigerio et al., 2019; [15]Sierksma et al., 2020
Microglia6	MHC High Microglia	Cd74, H2-Aa, H2-Ab1, H2-Eb1, H2-DMb1, H2-D1, B2m, H2-Dmb2, H2-K1, H2-T23, H2-Q7	[14]Sala Frigerio et al., 2019; [15]Sierksma et al., 2020
BMDM2	Glioma Associated BMDM	Spp1, Lyz2, Apoe, H2-D1, Cd74, Cd52	[11]Sankowski et al., 2019; [12]Ochocka et al., 2020; [13]Szulzewsky et al., 2015
BMDM3	Differentiating BMDM	Rpl27, Rps28, Rpl39, Rps29, Rpl38, Rpl41, Rps26, Rpl37a, Rpl36a	[16]Van Hove et al., 2019
BMDM4	Interferon Response BMDM	Ifit3, Isg15, Usp18, Ifit2, Ifi44, Irf7, Rtp4, Ifitm3, Phf11b, Stat1, Ccl12	[14]Sala Frigerio et al., 2019; [15]Sierksma et al., 2020; [17]Hammond et al., 2019

Supplementary Table 2: Gene signatures used to ascribe cell states were derived from published brain immune cell sequencing (references indicated in table and Supplementary References). Shown are genes among the top 20 differentially expressed genes between each cluster as compared to clusters containing the same cell type.

Supplementary Table 3

U87 Human Glioma	Control (pcDNA)	Control (pcDNA)	Control (pcDNA)	IL-33	IL-33	IL-33
Estimated Number of Cells	7,907	10,217	2,879	6,389	6,701	6,318
Mean Reads per Cell	17,668	12,240	55,194	19,993	21,798	20,890
Median Genes per Cell	1,081	1,056	1,434	1,143	783	1,314
Number of Reads	139,707,978	125,061,008	158,904,169	127,739,384	146,072,641	131,989,320
Valid Barcodes	96.60%	96.50%	96.50%	96.70%	96.80%	96.50%
Sequencing Saturation	71.80%	58.70%	85.00%	69.20%	78.70%	67.40%
RM to Genome	87.30%	85.80%	86.60%	86.10%	87.20%	86.10%
RM to Genome	85.60%	84.00%	84.70%	83.90%	84.80%	84.00%
RM to Intergenic Regions	4.60%	4.90%	5.30%	5.00%	4.80%	4.40%
RM to Intronic Regions	21.40%	22.20%	22.40%	22.10%	22.10%	21.70%
RM to Exonic Regions	59.70%	56.90%	56.90%	56.80%	58.00%	57.90%
RM to Transcriptome	56.70%	53.60%	53.90%	53.70%	55.00%	55.00%
RM Antisense to Gene	1.60%	1.80%	1.60%	1.70%	1.60%	1.50%
Fraction Reads in Cells	92.10%	92.60%	89.80%	92.00%	92.40%	88.00%
Total Genes Detected	16,021	16,138	14,991	16,080	15,805	16,444
Median UMI Counts per Cell	2,429	2,321	3,503	2,491	1,536	3,017

Supplementary Table 3: Sequencing metrics for U87 human glioma cells expressing empty pcDNA plasmid (Control) or pcDNA containing IL-33 (IL-33). cDNA libraries were sequenced on an Illumina NextSeq 500. RM: Reads Mapped.

Supplementary Table 4

**Initial Clustering**	
Percentage of mitochondrial genes expressed	5%
Minimum number of cells a feature is expressed in	3
Minimum number of genes expressed in a single cell	200
Maximum number of genes expressed in a single cell	2500
Dimensions	15
Resolution	0.3
**Re-clustering following removal of CD45- cells**	
Dimensions	15
Resolution	0.375

Supplementary Table 4: Analysis parameters used for quality control, dimensional reduction, and clustering on the R package Seurat (Seurat Version 3.1.5).

Supplementary Table 5

Cluster 10			
**Gene name**	**avg_logFC**	**pct.1**	**pct.2**
Ttr	7.449643618	0.942	0.112
Enpp2	4.808131647	0.925	0.002
Sostdc1	3.056210517	0.752	0
Clu	3.049991814	0.735	0.001
Ptgds	2.923785008	0.728	0.001
Chchd10	2.897573853	0.718	0.008
1500015O10Rik	2.711842485	0.667	0.001
Igfbp2	2.550056267	0.643	0.001
Fxyd1	2.437960821	0.588	0.001
Spint2	2.200095369	0.544	0.004
Mt3	2.187668351	0.503	0.001
Car2	2.161379052	0.558	0.002
Rbp1	2.083716586	0.48	0.001
Folr1	2.076977499	0.51	0.001
Kcnj13	1.947176712	0.466	0
Calml4	1.861563834	0.449	0
Hopx	1.85177159	0.469	0.004
2900040C04Rik	1.849288348	0.422	0
Hemk1	1.770582802	0.442	0.034
Atp1b1	1.760805573	0.432	0.003
Ppp1r1b	1.735046995	0.425	0.001
Cdkn1c	1.667613253	0.435	0.028
Clic6	1.591328932	0.378	0
Cryab	1.559129796	0.395	0.015
Cpe	1.53641519	0.374	0.001
Pcp4l1	1.453558855	0.344	0
Pcp4	1.359717183	0.272	0.002
Rdh5	1.350591308	0.347	0.005
Gsta4	1.34639403	0.357	0.006
Stk39	1.329551004	0.32	0.001

Supplementary Table 5: Top 30 differentially expressed genes in the non-immune cell (CD45-) population that was identified in the initial round of clustering and filtered out from subsequent analyses. The expression-signature showed considerable overlap with choroid plexus epithelial cells described previously by [18]Lun et al., 2015 (see Supplementary References).

Supplementary Table 6

REAGENT or RESOURCE	SOURCE	IDENTIFIER (Dilution)
Antibodies		
Mouse monoclonal anti-a-actin (Clone C4)	Millipore	Cat# MAB1501 (1:500)
Goat polyclonal anti-arginase 1 (Clone V-20)	Santa Cruz	Cat# sc-18354 (1:100)
Rabbit polyclonal anti-CD4 (Clone EPR6855)	Abcam	Cat# ab133616 (1:500)
Rat monoclonal anti-FOXP3 (FJK-16s)	Invitrogen	Cat#14-5773-82 (1:200)
Mouse monoclonal anti-CD163	Leica Biosystems	Cat# NCL-L-CD163 (1:200)
Mouse monoclonal anti-GFP (LGB-1)	Abcam	Cat# ab42560 (1:500)
Rabbit polyclonal anti- Iba1	Wako Laboratory Chemicals	Cat# 019-19741 (1:500)
Mouse monoclonal anti-human IL-33 (Clone Messy 1)	Enzo Life Sciences	Cat# ALX-804-840 (1:100)
Goat polyclonal anti-mouse IL-33	R & D Systems	Cat#AF3626 (1:100)
Mouse monoclonal anti-human nucleolin (Clone 4E2)	Abcam	Cat# ab13541 (1:500)
Rabbit polyclonal anti-Olig-2	Millipore	Cat# AB9610 (1:500)
Rabbit polyclonal anti-RFP	Abcam	Cat#ab34764 (1:500)
Rabbit monoclonal anti-pSTAT3 (Y705) (Clone D3A7)	Cell Signalling Technologies	Cat# 9145s (1:50)
Mouse monoclonal PTMScan® Phospho-Tyrosine	Cell Signalling Technologies	Cat # 5636 (40μl)
APC Rat anti-F4/80 (clone BM8)	eBioscience	Cat# 17-4801-80 (1:100)
Mouse monoclonal anti-ST2/IL-33R	R & D Systems	Cat#MAB523 (1:100)
APC anti-mouse CCR2 (Clone #475301)	R & D Systems	FAB5538A-025 (1:50)
APC Rat IgG2b	R & D Systems	IC013A (1:50)
AF700 anti-mouse CD11b (Clone M1/70)	BioLegend	Cat# 101222 (1:150)
AF700 Rat IgG2b, κ	BioLegend	Cat#400628 (1:150)
Purified anti-mouse CD16/32	BioLegend	Cat# 101302 (1:100)
BV785 anti-mouse CD45 (Clone 30-F11)	BioLegend	Cat# 103149 (1:600)
BV785 Rat IgG2b, κ	BioLegend	Cat# 400647 (1:600)
BV421 anti-mouse CD3 (Clone 17A2)	BioLegend	Cat#100228 (1:100)
BV421 Rat IgG2b, κ	BioLegend	Cat#400655 (1:100)
BV510 anti-mouse Ly6G (Clone 1A8)	BioLegend	Cat# 127633 (1:50)
BV510 Rat IgG2a, κ	BioLegend	Cat#400553 (1:50)
PerCP anti-mouse Ly6C (Clone HK1.4)	BioLegend	Cat# 128028 (1:200)
BV421 anti-mouse NKp46 (Clone 29A1.4)	BioLegend	Cat#137612 (1:100)
BV421 Rat IgG2a, κ	BioLegend	Cat#400549 (1:100)
APC anti-mouse NK1.1 (Clone PK136)	BioLegend	Cat#108710 (1:1000)
APC mouse IgG2a, κ	BioLegend	Cat#400219 (1:1000)
PeCy7 anti-mouse CD19	BioLegend	Cat#115520 (1:200)
PeCy7 Rat IgG2a, κ	BioLegend	Cat#400522 (1:200)
Bacterial and Virus Strains		
Biological Samples		
GBM tissue microarray paraffin sections and primary human GBM blocks	Clark H. Smith Neurologic and Pediatric Tumor Bank at the University of Calgary	N/A
Human adult or fetal brain tissue	University of Calgary	N/A
Chemicals, Peptides, and Recombinant Proteins		
Dulbecco's Modified Eagle's Medium (DMEM)	Invitrogen	Cat# 11960-044
Epidermal growth factor (EGF)	Peprotech	Cat#100-15
Fc-Block	Innovex Biosciences	Cat# NB309
Fetal bovine serum	Invitrogen	Cat# 12483-020
Fibroblast growth factor (FGF)	R&D systems	Cat#233-FB
G418	Invitrogen	Cat# 10131035

HBSS 1X	Gibco	14175-095
HBSS 10X	Gibco	14185-052
Hematoxylin	Millipore Sigma	Cat# GHS232-1L
Heparin sulfate	Sigma	Cat#H7640
Leptomycin B	Invitrogen	Cat# tlrl-lep; CAS 87081-35-4
L-glutamine	Invitrogen	Cat# 25030-081
NEAA	Invitrogen	Cat# 11140-050
Penicillin/streptomycin	Invitrogen	Cat# 15140-122
Percoll	Millipore Sigma	GE17-0891-01
Phorbol-12-myristate 13-acetate (PMA)	Sigma	Cat#P8139
Propidine iodide (PI)	Invitrogen	Cat#00-6990
RNAlater solution	Thermo Fisher Scientific	Cat# AM7020
Sep Pak C18 Column	Waters Corporation	Cat# WAT054945
Sodium pyruvate	Invitrogen	Cat# 11360-070
Trizol	Invitrogen	Cat# 15596026
Trypsin, TPCK	Worthington Biochemical Corporation	Cat# LS003740
Critical Commercial Assays		
Zombie Violet™ Fixable Viability Kit	Biolegend	Cat. # 423113
mirVana miRNA Isolation Kit	Thermo Fisher Scientific	Cat# AM1560
RNeasy Plus Micro kit	Qiagen	Cat. # 74034
3' IVT Express Kit	Ambion	Cat# 90122
Lipofectamine 2000 Transfection Reagent	Invitrogen	Cat# 11668027
PrimeView Human Gene Expression Array	Thermo Fisher Scientific	Cat# 901837
Envision and System-HRP Kit	DAKO	Cat# K4007
Alamar Blue	Invitrogen	Cat# Dal1100
Qiagen kit of RNeasy® Plus Micro	Qiagen	Cat# 74034
Opal 4-Color Automation IHC Kit	Perkin-Elmer	Cat# NEL800001KT
DNA-free DNA removal kit	Invitrogen	Cat# AM1906
Superscript III	Invitrogen	Cat# 18080093
nCounter Inflammation Panel (Mouse v2) gene expression code set	Nanostring Technologies	Cat# XT-CSO-MIN2-12
Chromium™ Single Cell 3' Library & Gel Bead Kit v2, 16 rxns	10x Genomics	120237
Chromium™ Single Cell A Chip Kit, 48 rxns	10x Genomics	120236
Chromium™ i7 Multiplex Kit, 96 rxns	10x Genomics	120262
High Sensitivity DNA Kit	Agilent Technologies	5067-4626
Kapa Library Quantification Kit	Roche	KK4824
NextSeq 500/550 High Output Kit v2 (150 cycles)	Illumina	FC-404-2002
Experimental Models: Cell Lines		
Human: U87MG cells	ATCC	HTB-14
Human: U251N cells	ATCC	
U87 pcDNA	[19]Ahn et al., 2016	
U87 IL-33	In this manuscript	
U87 IL-33 ΔNLS	In this manuscript	
U251pcDNA	[19]Ahn et al., 2016	
U251 IL-33	In this manuscript	
U251 IL-33 ΔNLS	In this manuscript	
Human: patient-derived brain tumor initiating cells	University of Calgary BTIC core facility	N/A

K1491	[20]Reilly et al., 2000, [21]Gursel et al., 2011	
K1492	[20]Reilly et al., 2000, [21]Gursel et al., 2011	
K1492 empty vector	In this manuscript	
K1492 mouse IL-33	In this manuscript	
U937	ATCC	CRL-1593.2
DF-1	ATCC	CRL-12203
Experimental Models: Organisms/Strains		
PDGFA-, PDGFB and shNF-1-induced glioma in *N/tv-a;Cdkn2a−/−;Ptenfl/fl mice (Xfm)*	[22]Ozawa et al., 2014	N/A
PDGFB -induced glioma in Cx3cr1GFP/WT;Ccr2^{RFP}/WT mice	[23]Chen et al., 2017	N/A
CB17 SCID mice (6 to 8-week-old female)	Charles River Laboratory	Strain code 236
CB57/BL6 mice (6 to 8 week old female)	Jackson	Strain code 0067
Oligonucleotides		
Recombinant DNA		
Human IL-33 pCMV6-XL5 vector	Origene	NM_033439; Cat# SC100114
pcDNA3.1 vector	Invitrogen	V79020
Human IL-33 ΔNLS mutant	In this manuscript	N/A
pLenti-EF1a-blank vector	Abm Inc.	Cat# LV588
mIL-33 pLenti-GIII-EF1a	Abm Inc	NM_001164724; Cat# LV503792
RCAS-hPDGFA-myc/6xHis	[22]Ozawa et al., 2014	N/A
RCAS-hPDGFB-HA	[22]Ozawa et al., 2014	N/A
RCAS-shNf1	[22]Ozawa et al., 2014	N/A
RCAS-Cre	[22]Ozawa et al., 2014	N/A
Software and Algorithms		
Imagescope v12.2.2.5015	Leica Biosystems	N/A
GraphPad Prism 8	Graphpad software	N/A
GeneChip Command Console Software (AGCC)	Affymetrix	N/A
Partek Genomics Suite 6.0	Partek Incorporated, USA	N/A
In Cell developer toolbox 1.9.1	GE healthcare	N/A
nSolver Analysis Software v3	NanoString Technologies	N/A
NanoString nCounter gene expression platform	NanoString Technologies	N/A
QuPath (0.1.2.)	[https://qupath.github.io/]	N/A
Cell Ranger v3.1	10X Genomics	[http://10xgenomics.com]
R v3.6.3	The R project	[https://www.r-project.org/]
Seurat v3.1.5	[24]Butler et al., 2018	[https://satijalab.org/seurat/]
Harmony v1.0	[25]Korsunsky et al., 2019	[https://github.com/immunogenomics/harmony]
SCANPY v1.4.6	[26]Wolf et al., 2018	[https://github.com/theislab/scanpy]
SCORPIUS v1.0.6	[27]Cannoodt et al., 2016	[https://github.com/rcannood/SCORPIUS]

Ingenuity Pathway Analysis (IPA)	QIAGEN	N/A
genesorteR v0.4.3	[28]Ibrahim and Kramann, 2019	[https://github.com/mahmoudibrahim/genesorteR]
clusterProfiler v3.14.3	[29]Yu et al., 2012	[https://github.com/YuLab-SMU/clusterProfiler]

Supplementary Table 6: Table provides the list of all reagent and resource including relevant source, references and websites. See also Supplementary References.

Shown are Western blots for Figures 1E (IL33); 2A (IL33 and actin); Supplementary Figure 2C (ST2 and actin).

Figure 1E – (IL33)

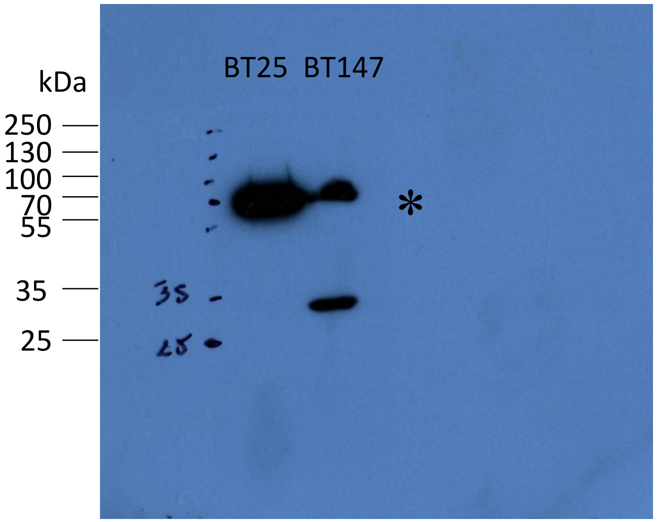

Asterisk * indicates non-specific binding of the antibody to mouse albumin present in large amounts in the TIF.

Figure 2A – (IL33)

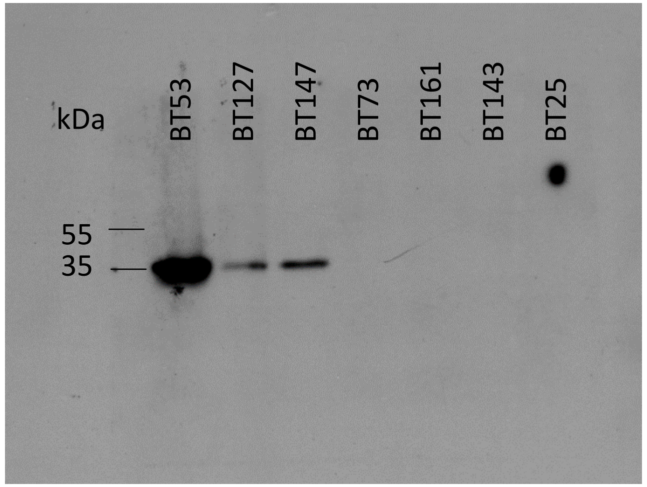

Figure 2A – (Actin)

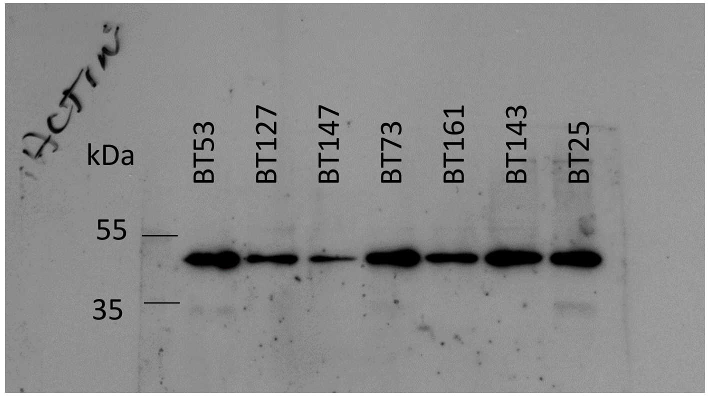

Supplementary Figure 2C – (ST2)

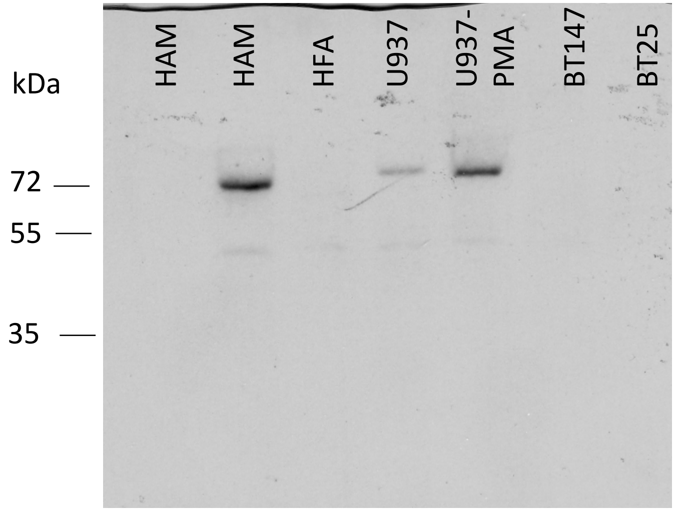

Supplementary Figure 2C – Actin

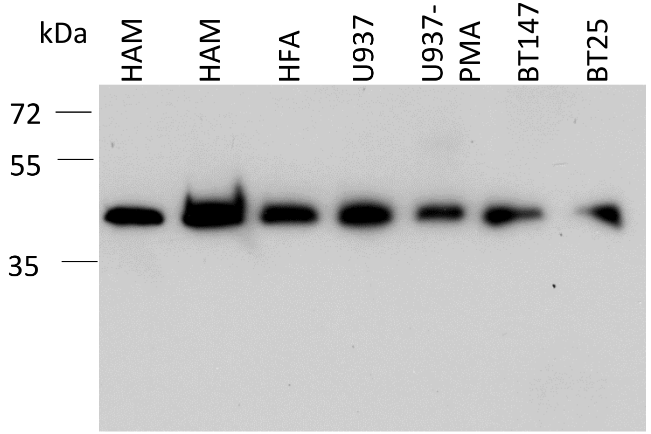

Figure 3A (Upper Panel) shows IL33 and Actin Western blots for U87 human glioma expressing IL33 and ΔNLS cell clones cell lysates and condition media.

Supplementary Figure 3A (Lower Panel) shows Western blots for U251 human glioma expressing IL33 and ΔNLS cell clones cell lysates and condition media.

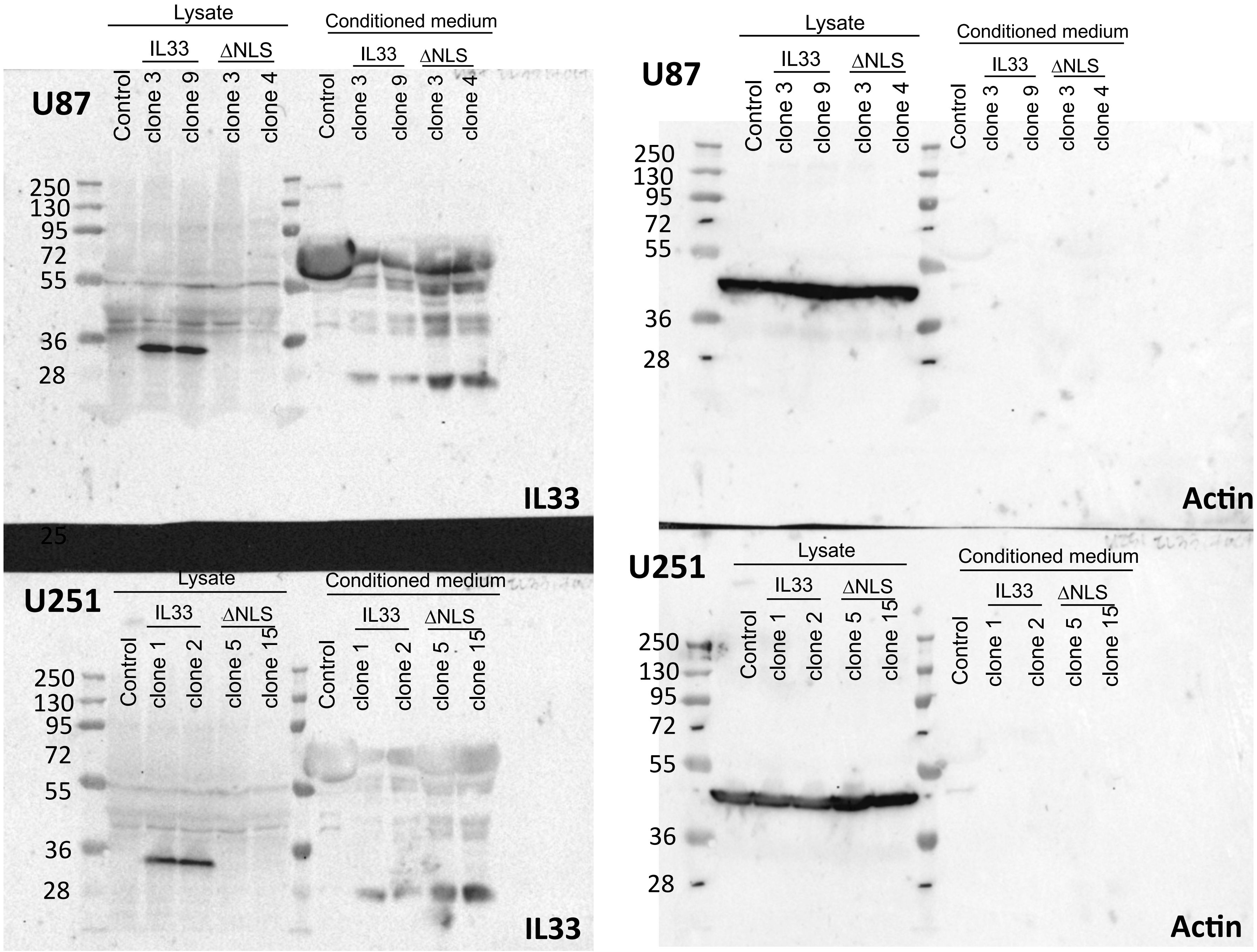

Supplementary Figure 3H – Image shows Western blot for IL33 (left) and Actin (right).

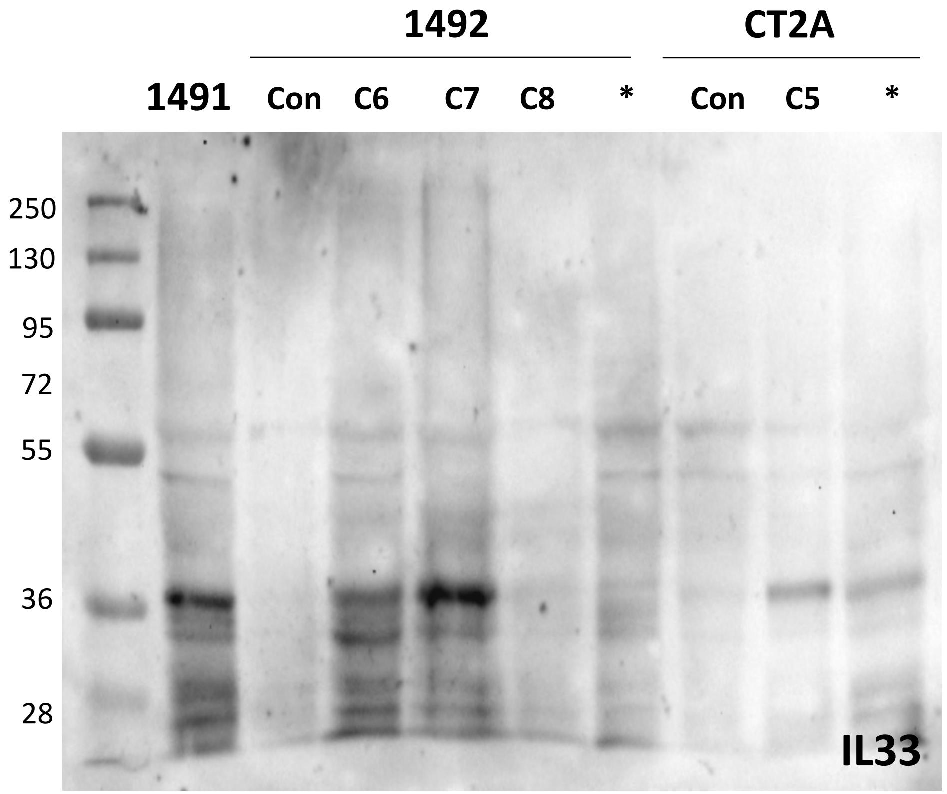

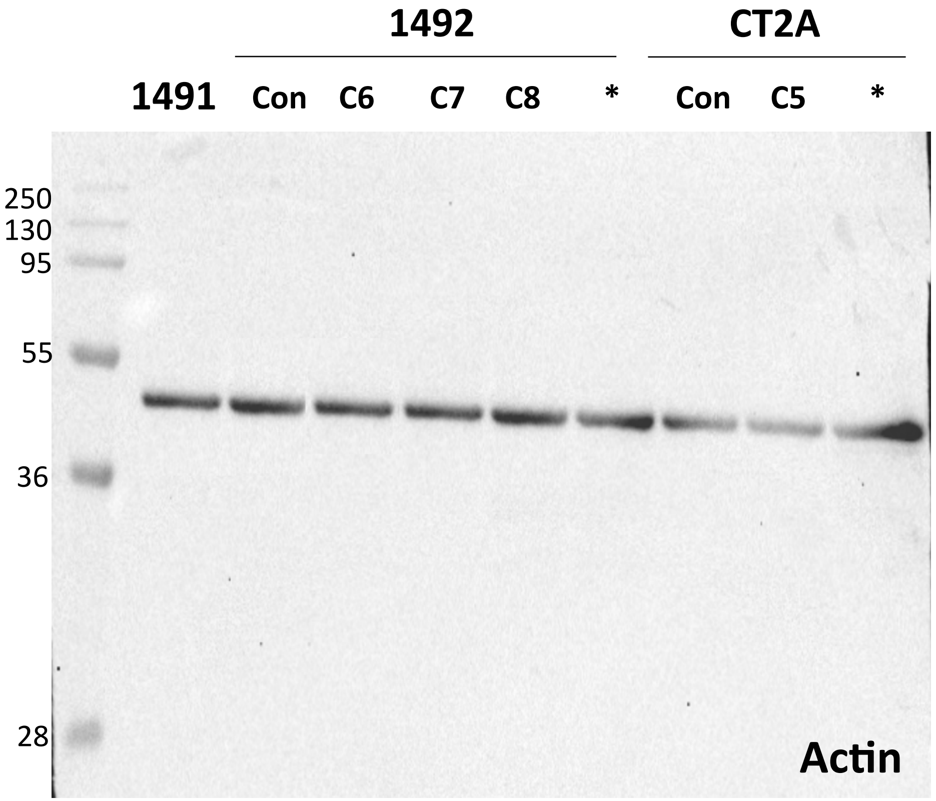

Western blot shows K1491, K1492 vector control (Con) and IL33 expressing cell clones 6, 7, 8 (C6, C7, C8), CT2A Control (Con) and Clone 5 (C5).
Asterisk (*) indicates K1492 cell pool prior to cloning.
For completeness the entire Western Blot is shown however the CT2A data was not discussed in the manuscript.

Supplementary References

1. Bennett ML*, et al.* New tools for studying microglia in the mouse and human CNS. *Proc Natl Acad Sci U S A* **113**, E1738-1746 (2016).

2. Haage V*, et al.* Comprehensive gene expression meta-analysis identifies signature genes that distinguish microglia from peripheral monocytes/macrophages in health and glioma. *Acta Neuropathol Commun* **7**, 20 (2019).

3. Sousa C*, et al.* Single-cell transcriptomics reveals distinct inflammation-induced microglia signatures. *EMBO Rep* **19**, (2018).

4. Ziegler-Heitbrock L*, et al.* Nomenclature of monocytes and dendritic cells in blood. *Blood* **116**, e74-80 (2010).

5. Tsou CL*, et al.* Critical roles for CCR2 and MCP-3 in monocyte mobilization from bone marrow and recruitment to inflammatory sites. *J Clin Invest* **117**, 902-909 (2007).

6. Cursons J*, et al.* A Gene Signature Predicting Natural Killer Cell Infiltration and Improved Survival in Melanoma Patients. *Cancer Immunol Res* **7**, 1162-1174 (2019).

7. Walzer T*, et al.* Identification, activation, and selective in vivo ablation of mouse NK cells via NKp46. *Proc Natl Acad Sci U S A* **104**, 3384-3389 (2007).

8. Lakschevitz FS, Visser MB, Sun C, Glogauer M. Neutrophil transcriptional profile changes during transit from bone marrow to sites of inflammation. *Cell Mol Immunol* **12**, 53-65 (2015).

9. Ryckman C, Vandal K, Rouleau P, Talbot M, Tessier PA. Proinflammatory activities of S100: proteins S100A8, S100A9, and S100A8/A9 induce neutrophil chemotaxis and adhesion. *J Immunol* **170**, 3233-3242 (2003).

10. Masuda T, Sankowski R, Staszewski O, Prinz M. Microglia Heterogeneity in the Single-Cell Era. *Cell Rep* **30**, 1271-1281 (2020).

11. Sankowski R*, et al.* Mapping microglia states in the human brain through the integration of high-dimensional techniques. *Nat Neurosci* **22**, 2098-2110 (2019).

12. Ochocka N*, et al.* Single-cell RNA sequencing reveals functional heterogeneity and sex differences of glioma-associated brain macrophages. *bioRxiv*, (2020).

13. Szulzewsky F*, et al.* Glioma-associated microglia/macrophages display an expression profile different from M1 and M2 polarization and highly express Gpnmb and Spp1. *PLoS One* **10**, e0116644 (2015).

14. Sala Frigerio C*, et al.* The Major Risk Factors for Alzheimer's Disease: Age, Sex, and Genes Modulate the Microglia Response to Abeta Plaques. *Cell Rep* **27**, 1293-1306 e1296 (2019).

15. Sierksma A*, et al.* Novel Alzheimer risk genes determine the microglia response to amyloid-beta but not to TAU pathology. *EMBO Mol Med* **12**, e10606 (2020).

16. Van Hove H*, et al.* A single-cell atlas of mouse brain macrophages reveals unique transcriptional identities shaped by ontogeny and tissue environment. *Nat Neurosci* **22**, 1021-1035 (2019).

17. Hammond TR*, et al.* Single-Cell RNA Sequencing of Microglia throughout the Mouse Lifespan and in the Injured Brain Reveals Complex Cell-State Changes. *Immunity* **50**, 253-271 e256 (2019).

18. Lun MP*, et al.* Spatially heterogeneous choroid plexus transcriptomes encode positional identity and contribute to regional CSF production. *J Neurosci* **35**, 4903-4916 (2015).

19. Ahn BY*, et al.* Glioma invasion mediated by the p75 neurotrophin receptor (p75(NTR)/CD271) requires regulated interaction with PDLIM1. *Oncogene* **35**, 1411-1422 (2016).

20. Reilly KM, Loisel DA, Bronson RT, McLaughlin ME, Jacks T. Nf1;Trp53 mutant mice develop glioblastoma with evidence of strain-specific effects. *Nat Genet* **26**, 109-113 (2000).

21. Gursel DB*, et al.* Control of proliferation in astrocytoma cells by the receptor tyrosine kinase/PI3K/AKT signaling axis and the use of PI-103 and TCN as potential anti-astrocytoma therapies. *Neuro Oncol* **13**, 610-621 (2011).

22. Ozawa T*, et al.* Most human non-GCIMP glioblastoma subtypes evolve from a common proneural-like precursor glioma. *Cancer Cell* **26**, 288-300 (2014).

23. Chen Z*, et al.* Cellular and Molecular Identity of Tumor-Associated Macrophages in Glioblastoma. *Cancer Res* **77**, 2266-2278 (2017).

24. Butler A, Hoffman P, Smibert P, Papalexi E, Satija R. Integrating single-cell transcriptomic data across different conditions, technologies, and species. *Nat Biotechnol* **36**, 411-420 (2018).

25. Korsunsky I, *et al.* Fast, sensitive and accurate integration of single-cell data with Harmony. *Nat Methods* **16**, 1289-1296 (2019).

26. Wolf FA, Angerer P, Theis FJ. SCANPY: large-scale single-cell gene expression data analysis. *Genome Biol* **19**, 15 (2018).

27. Cannoodt R, Saelens W, Saeys Y. Computational methods for trajectory inference from single-cell transcriptomics. *Eur J Immunol* **46**, 2496-2506 (2016).

28. Ibrahim MM, Karmann R. GenesorteR: feature ranking in clustered single cell data. *bioRxiv* https://www.biorxiv.org/content/10.1101/ **676379v2**, (2019).

29. Yu G, Wang LG, Han Y, He QY. clusterProfiler: an R package for comparing biological themes among gene clusters. *OMICS* **16**, 284-287 (2012).
