# Supplementary material for: Glioma-derived IL-33 orchestrates an inflammatory brain tumor microenvironment that accelerates glioma progression
Source: Nat Commun. 2020 Oct 5;11:4997. doi: 10.1038/s41467-020-18569-4 (PMC7536425; doi:10.1038/s41467-020-18569-4)
Supplement: Supplementary file 2 — Descriptions of Additional Supplementary Files [file 41467_2020_18569_MOESM2_ESM.pdf]

Descriptions of Additional Supplementary Files

Supplementary data 1

Description: Cluster information and quality control metric. Table contains characterization and quality control metrics for all 14 clusters including the number of cells in each cluster, the minimum, maximum, and average number of genes detected, number of UMI's, and percent mitochondrial content, and up to the top 100 differentially expressed genes between each cluster and all other clusters.
